# Photocyclization of 8‑Aryloxybenzo[*e*][1,2,4]triazines Revisited: Unambiguous Structural Assignment of Planar Blatter Radicals by Correlation NMR Spectroscopy

**DOI:** 10.1021/acs.joc.5c00741

**Published:** 2025-06-27

**Authors:** Hemant K. Singh, Sławomir Kaźmierski, Piotr Kaszyński

**Affiliations:** † 86897 Centre of Molecular and Macromolecular Studies, Polish Academy of Sciences, 90-363 Łódź, Poland; ‡ Faculty of Chemistry, University of Łódź, 91-403 Łódź, Poland; § Department of Chemistry, Middle Tennessee State University, Murfreesboro, Tennessee 37130, United States

## Abstract

The recent discovery of a photo-Smiles rearrangement and the development of effective analytical methods prompted structural investigation of the previously reported planar Blatter radicals obtained by photocyclization of 8-aryloxy-3-phenylbenzo­[*e*]­[1,2,4]­triazines. Thus, seven freshly prepared radicals were cleanly converted to their *leuco* forms and analyzed by 2D correlation ^1^H NMR methods (COSY, TOCSY, and ROSEY). Results demonstrate that all investigated 8-aryloxy-3-phenylbenzo­[*e*]­[1,2,4]­triazines undergo photocyclization with Smiles rearrangement and exclusive formation of a single rearranged product. This work corrects the previously reported structural assignment and DFT data of planar Blatter radicals and further demonstrates the generality of this new variation of the photo-Smiles rearrangement.

## Graphical abstract

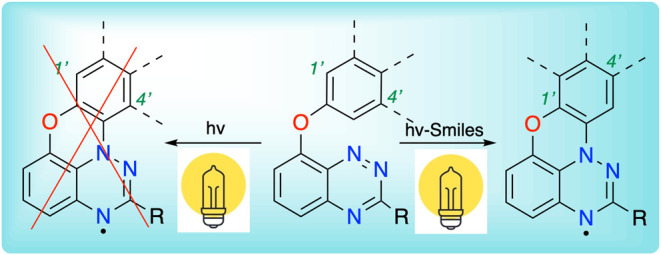

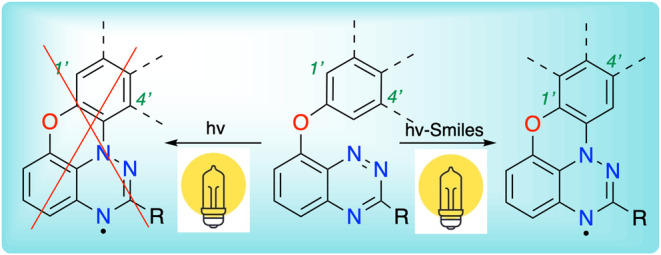

## Introduction

Stable radicals^1^ are of importance in contemporary science and for the development of new materials^2,3^ with controlled properties for emerging technologies.^4–6^ Among them are the prototypical Blatter radical^7^ (Figure 1) and its derivatives,^8^ which generally exhibit high stabilities and electronic properties attractive for a range of applications.^9^ Recently, we demonstrated the formation of planar Blatter radical^10^ (Figure 1) and its derivatives^11^ containing the phenoxazine fragment by photocyclization of appropriate 8-aryloxybenzo­[*e*]­[1,2,4]­triazines.^12,13^ This method was used to obtain a series of Blatter helicene radicals.^14^

**Figure 1:**
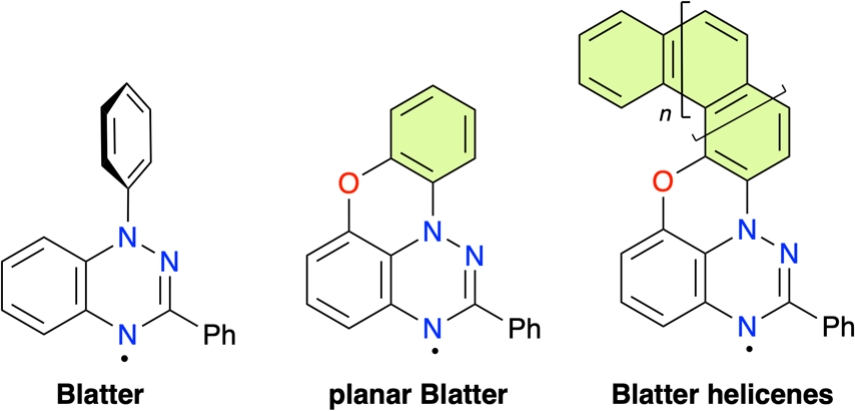
Structures of the prototypical Blatter, planar Blatter, and Blatter helicene radicals.

With the limited number of single-crystal XRD structures and unavailable NMR analysis, the structures of Blatter radical photoproducts were assigned based on experimental results and DFT calculations. Thus, it was observed that substituents in 8-(2-nitrophenoxy) and 8-(2-bromophenoxy) substrates of the general structure **S** (Figure 2) undergo a addition–elimination process during photocyclization, giving rise to the same planar Blatter radical, as obtained by photocyclization of the unsubstituted 8-phenoxy derivative and other methods.^12^ The structure of the product was confirmed with XRD analysis.^10^ Therefore, it was assumed that the formation of other planar Blatter radicals from substrate **S** containing larger aryloxy groups follows the same simple addition–elimination process taking place in the exited state. The regioselectivity and formation of product either **A** or **B** (Figure 2) was decided based on DFT reaction path analysis in the S_1_ state, which clearly preferred one of the two possible products.^12,13^ A rearrangement was not considered at that time, since such a mechanism was not known in the literature.

**Figure 2:**
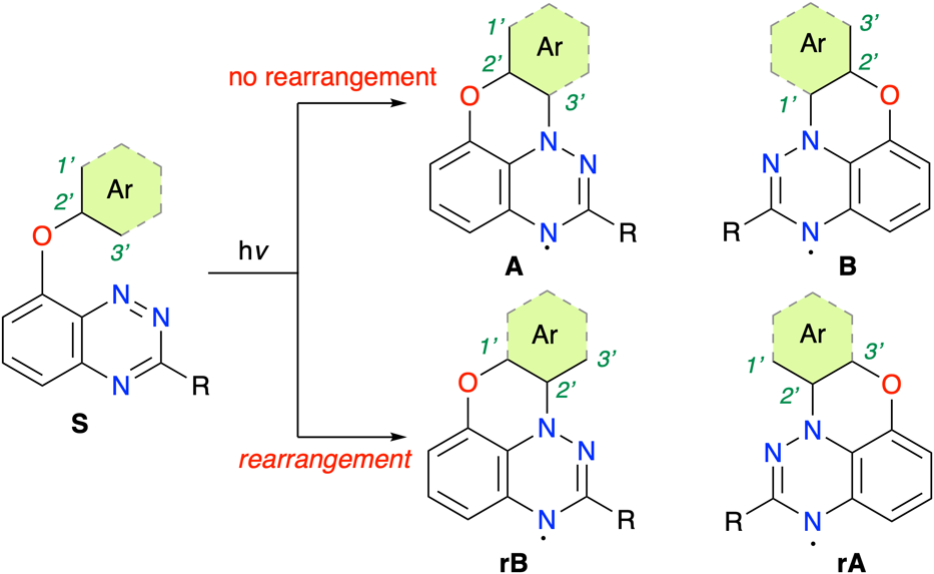
A general scheme for photocyclization of 8-aryloxybenzo­[*e*]­[1,2,4]­triazines giving regioisomeric radicals **A** and **B** without oxygen migration or **rA** and **rB** with rearrangement.

A recent success with obtaining XRD structures^14^ and detailed 2D NMR analysis of the *leuco* forms^15^ revealed that the structures of Blatter helicenes (Figure 1) are not as expected and their fully regioselective formation involves a formal migration of the oxygen atom. Extensive DFT calculations prompted by this discovery revealed a new photo-Smiles rearrangement mechanism,^15^ which prefers the formation of one of the regio-isomers **rA** or **rB** (Figure 2) on the basis of the number of Clar’s sextets in the intermediates. This finding suggested that other 8-aryloxy substrates **S** can also undergo the newly discovered photo-Smiles rearrangement, and the structure of the products may be different than those originally assigned.

Herein, we revisit the previously reported planar Blatter radicals **1** obtained by the photocyclization of aryloxy precursors **2** with the goal of determining the scope of the photo-Smiles rearrangement and correcting the structural and analytical data assignments. To accomplish this, we take advantage of the recently developed 2D NMR correlation analysis of *leuco* forms of Blatter radicals^15^ and provide their unambiguous structural characterization.

## Results and Discussion

For the structural assignment of the photocyclization products, Blatter radicals **1** were converted to their *leuco* forms **1-H** by treatment with ascorbic acid in DMSO-*d*_6_ containing a drop of CD_2_Cl_2_ and D_2_O (Scheme 1). For the purpose of this analysis, most radicals **1** were freshly obtained by photocyclization of appropriate 8-aryloxybenzo­[*e*]­[1,2,4]­triazines **2**, as described previously.^12,13^

**Scheme 1:**
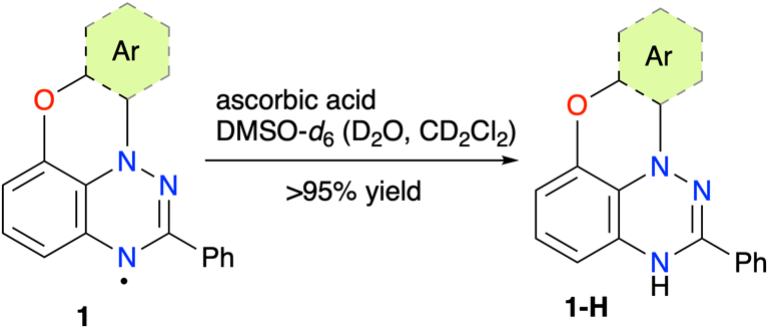
Preparation of the *leuco* Derivatives **1-H**

### NMR Structural Analysis of **1-H**

^1^H NMR spectra of freshly prepared samples of **1-H** revealed a single species with typically well-resolved multiplets in the aromatic region. This indicates a complete regioselectivity of the cyclization process. In each compound, there are three distinct spin systems associated with rings *Ph*, *B*, and *T*, which were identified by H–H correlation spectroscopy (COSY and TOCSY^16^) on the basis of their characteristic number of interacting hydrogen nuclei and coupling patterns (see Tables 1, 2, and 3 and the ). In addition, there are isolated spins (singlets) or two-spin systems (pairs of doublets), depending on the structure. The subsequent analysis using the ROSEY^17^ method identified key intersystem interactions, which allowed for differentiation of the isomers and unambiguous structural assignments.

**Table 1:**
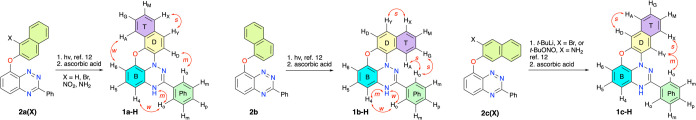
Preparation of Naphthalene-Fused Radicals and Chemical Shifts of Characteristic Protons in Their *leuco* **1-H** ^a^

	H_4_	H_5_	H_6_		H_A_	H_G_	H_M_	H_X_		H_D_	H_Y_		H_o_	H_m_ and H_p_		N–H
*leuco*	*d*	*t*	*d*		*d*	*t*	*t*	*d*					*d*	*m*		br s
**1a-H** ^b^	6.39	6.76	6.53		7.89	7.46	7.35	7.77		7.59 *d*	7.56 *d*		7.85	7.53–7.46		8.87
**1b-H**	6.62	6.89	6.54		8.52	7.45	7.39	7.80		7.08 *d*	7.57 *d*		7.83	7.53–7.46		9.33
**1c-H**	6.24	6.60	6.34		7.62	7.24	7.18	7.56		7.35 *s*	7.13 *s*		7.85	7.53–7.46		8.80

a Recorded at 500 MHz in DMSO-*d*_6_. The red arrows in the structures indicate the observed through-space H···H interactions assigned as weak (*w*), medium (*m*), and strong (*s*) NOE. The principal multiplicity of the signal is designated as singlet (*s*), doublet (*d*), triplet (*t*), or unresolved multiplet (*m*).

b Data taken from ref 15.

**Table 2:**
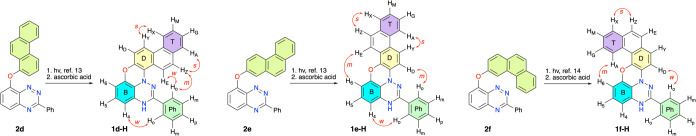
Preparation of Phenanthrene-Fused Radicals and Chemical Shifts of Characteristic Protons in Their *leuco* **1-H** ^a^

	H_4_	H_5_	H_6_		H_A_	H_G_	H_M_	H_X_		H_D_	H_Y_	H_E_	H_Z_		H_o_	H_m_ and H_p_		N–H
*leuco*	*d*	*t*	*d*		*d*	*t*	*t*	*d*		*d*	*d*	*d*	*d*		*d*	*m*		br s
**1d-H**	6.64	6.91	6.58		7.93	7.60	7.65	8.69		7.22	8.46	8.41	7.79		7.85	7.54–7.46		9.39
**1e-H**	6.38	6.75	6.54		8.64	7.63	7.56	7.91		7.65	8.40	7.91	7.79		7.86	7.53–7.46		8.87
**1f-H** ^b^	6.37	6.75	6.71		9.42	7.66–7.58 *m*		7.88			7.66–7.58 *m*				7.85	7.53–7.46		^c^

a Recorded at 500 MHz in DMSO-*d*_6_. The red arrows in the structures indicate the observed through-space H···H interactions assigned as weak (*w*), medium (*m*), and strong (*s*) NOE. The principal multiplicity of the signal is designated as singlet (*s*), doublet (*d*), triplet (*t*), or unresolved multiplet (*m*).

b Data taken from ref 15.

c Not observed.

**Table 3:**
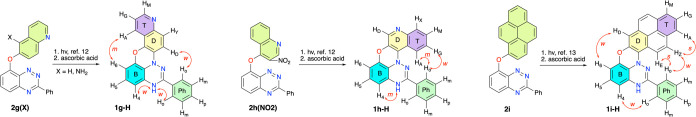
Preparation of Quinoline- and Pyrene-Fused Radicals and Chemical Shifts of Characteristic Protons in Their *leuco* **1-H** ^a^

	H_4_	H_5_	H_6_		H_A_	H_G_	H_M_	H_X_		H_D_	H_Y_		H_E_	H_Z_		H_o_	H_m_ and H_p_		N–H
*leuco*	*d*	*t*	*d*		*d*	*t*		*d*			*d*		*d*	*d*		*d*	*m*		br s
**1g-H**	6.41	6.77	6.51		8.28	7.45	8.70 *d*	–		7.67 *d*	7.81		–	–		7.85	7.54–7.46		8.92
**1h-H**	6.46	6.80	6.58		7.92	7.54	7.59 *t*	7.86		8.89 *s*	–		–	–		7.86	7.53–7.46		9.03
**1i-H**	6.61	6.91	6.62		8.15	7.94	8.15 *d*	–		7.73 *s*	–		8.76	8.07		7.90	7.54–7.46		–

a Recorded at 500 MHz in DMSO-*d*_6_. The red arrows in the structures indicate the observed through-space H···H interactions assigned as weak (*w*), medium (*m*), and strong (*s*) NOE. The principal multiplicity of the signal is designated as singlet (*s*), doublet (*d*), triplet (*t*), or unresolved multiplet (*m*).

In all derivatives, the pseudo doublet of *Ph* spin system (H_o_ at ∼7.85 ppm) interacts with three-spin system *B* indicating the position of the H_4_ proton. In addition, in many analyzed compounds, there are observable NOE signals due to the H_o_···HN and H_4_···HN interactions. The most diagnostic interactions for distinguishing isomers are between spin system *T* and H_o_ (*Ph*) or H_6_ (*B*).

NMR analysis of the product of photocyclization of 2-naphthyloxy derivative **2a­(H)** (Table 1) revealed significant interactions of the *T* and *B* spin systems (between the H_A_ and H_6_ protons), while H_o_ (*Ph*) interacts with the two-spin system *D*. These results are consistent with the structure of **1a-H** resulting from the photocyclization of **2a­(H)** with a Smiles rearrangement. The product that would be expected from **2a­(H)** without rearrangement is **1b-H**, in which H_o_ interacts with H_A_ and H_G_ protons (system *T*). This product is obtained by photocyclization of 1-naphthyloxy derivative **2b**, indicating that it also undergoes a Smiles rearrangement. For comparative purposes, the third regioisomer **1c** was obtained using either the intramolecular aryllithium addition (from **2c­(Br)** precursor) or by the aza-Pschorr cyclization (using **2c­(NH**_**2**_ **)** precursor).^12^ In the case of **1c-H** spin system *T* did not interact either with *B* or with *Ph*. Instead, the latter showed NOE signals with the two characteristic singlets, and the structure was confirmed with a single-crystal XRD analysis.^12^

All three radicals (**1a**, **1b**, and **1c**) show distinct behavior in TLC analysis, which was used to determine that other precursors (**2a­(Br)**, **2a­(NH**_**2**_ **)**, and **2a­(NO**_**2**_ **)**) also form the same rearrangement product **1a**.^12^

A similar analysis of products of photocyclization of phenanthrenyloxy derivatives **2d**–**2f** also indicated Smiles rearrangement in all three cases. Thus, irradiation of **2f** results in migration of the oxygen atom from the C(3′) position to C(4′) in the bay area of the phenanthrene system in **1f**.^14^ This migration is apparent from a strong NOE signal between *B* and *T* spin systems in **1f-H**, instead of *T* and *Ph* expected for the nonrearranged product.^13^ In 1-phenanthrenyloxy derivative **2d**, oxygen atom migration from the C(1′) to C(2′) position leads to product **1d-H**, with weak to moderate H_o_···H_E_ and H_o_···H_Z_ interactions. Further analysis shows that H_Z_ has strong interactions with the *T* spin system (H_Z_···H_A_), while the other two-spin system *D* shows a strong NOE signal with *T* (H_X_···H_Y_ correlation) and a very weak correlation with *B* (H_D_···H_6_). This connectivity path is consistent with the structure **1d-H**, hence, the formation of **1d** in the photocyclization of **2d**.

In the product of the photocyclization of **2e**, the situation is similar, although spin system *D* strongly correlates with *T* (H_Y_···H_A_) and interacts with *Ph* (H_D_···H_o_). The other two-spin system shows moderate interactions with *B* (H_E_···H_6_) and *T* (H_Z_···H_X_). This is consistent with the structure of **1e-H** and the formation of **1e** in the photocyclization of **2e**.

Analysis of the *leuco* derivative **1g-H** indicates that both precursors, unsubstituted 6-quinolinoxy **2g­(H)** and containing the C(5′)-amino group **2g­(NH**_**2**_ **)**, give the same product **1g** with the migrated oxygen atom. Thus, two-spin system *D* in **1g-H** shows weak NOE with *Ph* (H_D_···H_o_), while the three-spin system *T* exhibits moderate interactions with *B* (H_A_···H_6_). Similarly, oxygen migrates from C(4′) in **2h­(NO**_**2**_ **)** to C(3′) position in **1h**, as evident from moderate interactions between *T* and *Ph* (H_A_···H_o_), and complete magnetic isolation of the unique singlet H_D_.

Finally, analysis of photocyclization of 1-pyrenyloxy derivative **2i** also revealed migration of the oxygen atom from C(1′) to the C(2′) position. This is evident from NOE correlation of the unique singlet H_D_ with H_6_ of *B* and a two-spin system with *Ph* in **1i-H**, while in the unrearranged product, these interactions would be reversed.

### Mechanistic Analysis

The above NMR analysis demonstrates that the photocyclization of 8-aryloxybenzo­[*e*]­[1,2,4]­triazines **2** and the formation of radicals **1** occur with a rearrangement and full regioselectivity. The structures of the resulting radicals are consistent with the mechanism recently proposed^15^ for unsubstituted aryloxy derivatives **2** (X = H) shown in Figure 3. According to this mechanism, photoexcitation of **2** leads to the S_1_ state 2* with ^1^(*n*,π*) character localized on the benzo­[*e*]­[1,2,4]­triazine fragment. The subsequent ISC facilitated by the ^3^(π,π*) T_2_ state leads to ^3^(*n*,π*) T_1_ also being localized on the heterocycle. A high-energy π HOMO localized solely on the aryloxy fragment donates an electron to the formally half-occupied *n* orbital of the benzo­[*e*]­[1,2,4]­triazine, resulting in charge-separated species **2** ^**Z**^, which consists of benzo­[*e*]­[1,2,4]­triazine radical anion and aryloxy radical cation fragments. The subsequent intramolecular attack of the N(1) lone pair on the most electrophilic *ipso* carbon atom of the aryloxy substituent in zwitterion **2** ^**Z**^ gives the spiro-oxazole intermediate **3**, which, upon ISC, opens up to zwitterion **4**. The anionic oxygen atom in **4** attacks the *ortho* position, either C(1′) (*path a*) or C(3′) (*path b*), of the aryloxy fragment giving rise to a tautomeric *leuco* form, which can further tautomerize to **1-H** or undergo oxidation directly to radical **1**. DFT calculations demonstrated that the regioselectivity of the oxygen shift, either in *path a* or *path b* in Figure 3, is governed by the stability of the resulting *leuco* form, which, in turn, depends on the number of Clar’s sextets. Thus, the regioisomeric radical **1-rB** observed in all these photoreactions has a larger number of Clar’s sextets in the *lecuo* form. Support for this mechanism is provided by a recently obtained XRD structure of zwitterion **4** isolated as a byproduct in photocyclization of a higher helicenyloxybenzo­[*e*]­[1,2,4]­triazine. Heating this zwitterion in AcOEt solutions leads to the formation of radical **1**.^18^

**Figure 3:**
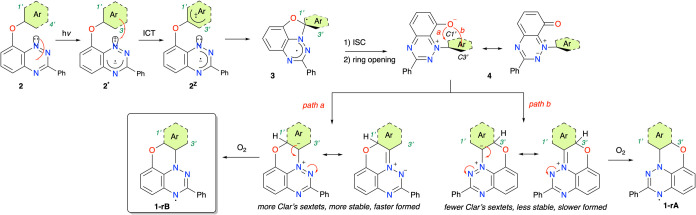
Proposed mechanism for photochemical transformation of 8-aryloxybenzo­[*e*]­[1,2,4]­triazines **2** to radicals **1**.

The role of substituent X in the proposed mechanism has not been studied computationally. Nevertheless, the structures of photoproducts obtained from **2a­(X)** indicate full consistence with this proposed mechanism, in which the key step is the electron transfer from the π HOMO, solely associated with the aryloxy, to the (*n*,π*) state localized on the benzo­[*e*]­[1,2,4]­triazine, leading to charge separated species **2** ^**Z**^. An example of such a localized HOMO is shown in Figure 4 for the 2-naphthyloxy derivative **2a­(H)**.

**Figure 4:**
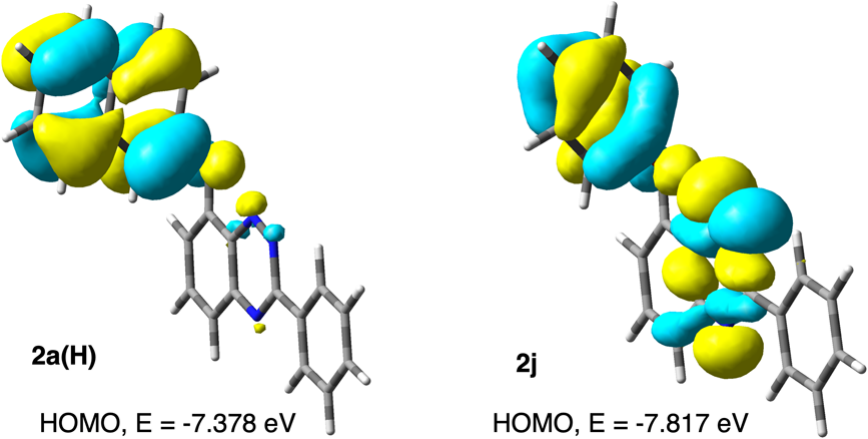
UCAM-B3LYP/6–311G­(d,p) derived contours of the HOMO for **2a­(H)** (left) and **2j** (right) for the S_0_ state at the triplet geometry. MO *isovalue* = 0.03).

DFT calculations showed that this condition is not satisfied for the phenoxy derivative **2j**: the HOMO is delocalized and encompasses the phenoxy π and lone pairs *n* (Figure 4, right), which prevents the formation of a persistent charge separated species **2** ^**Z**^ **j** that is prerequisite for spirooxazole formation and Smiles rearrangement. In the absence of the persistent zwitterion, the nonpolar triplet apparently undergoes cyclization to the nonrearranged *leuco* product in the triplet state, which upon oxidation gives radical **1j**. DFT calculations indicate that the activation enthalpy for cyclization of **2jT**, Δ*H* ^⧧^, is 8.55 kcal mol^–1^ in the AcOEt dielectric medium, while the overall cyclization process is mildly exothermic (Δ*H* = −9.26 kcal mol^–1^, Figure 5). It is possible that a similar mechanism operates for *ortho* substituted phenoxy derivatives giving the same radical **1j**. This mechanism is supported by experimental evidence.^19^

**Figure 5:**
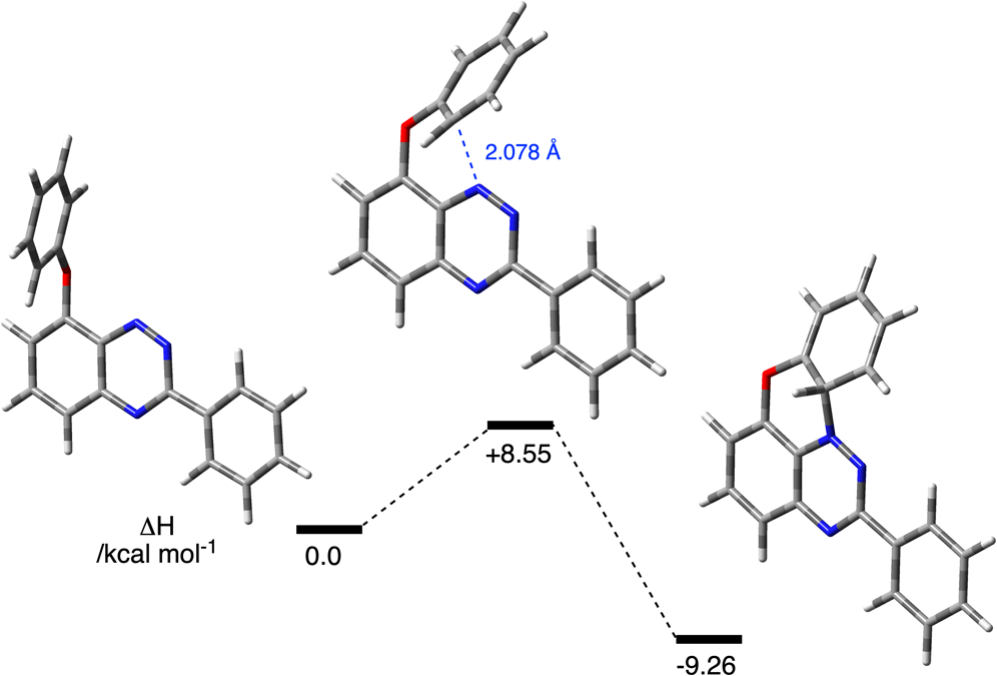
Enthalpies relative to **2j** in the T_1_ state (Δ*H* = 0 kcal mol^–1^) for cyclization of **2j** without rearrangement obtained at the UCAM-B3LYP/6–311G­(d,p) level of theory in an AcOEt dielectric medium. All species are in the triplet state.

### Corrected Electronic Properties of Radicals **1**

With the determined correct structures of radicals **1**, their experimental electronic properties, such as the absorption spectra, redox potentials, and *hfcc* are now correctly attributed and listed in Table 4. In addition, properties of these newly determined structures were re-evaluated by uniform DFT methods using geometries obtained at the UB3LYP/6–31G­(2d,p) level of theory in vacuum. Some results are shown in Table 4, while full information is provided in the .

**Table 4:** Corrected Assignment of Experimental Electronic Parameters of Radicals **1**

	λ_max_ ^a^ (nm)	λ_max_ DFT^b^ (nm)	*f*	*E*_g_^opt^^c^ (eV)	αHOMO^b^ (eV)	*E*_1/2_^–1/0^ (V)^d^	*E*_1/2_^0/+1^ (V)^d^	*E*_cell_^e^ (V)	α_N(12)_ ^f^ (G)	α_N(1)_ ^f^ (G)	α_N(3)_ ^f^ (G)	RDV^–1^ ^g^	comments: labels in previous ref.
**1a**	707.5	563.7	0.061	1.67	–6.113	–1.354	–0.212	1.14	7.60	4.10	4.24	3.835	ref 12 **1b**; ref 13 **1c**
**1b**	721	569.5	0.037	1.64	–6.108	–1.262	–0.206	1.05	7.74	3.96	4.04	3.611	ref 12 **1d**; ref 13 **1b**
**1c**	681.5	545.8	0.068	1.69	–6.238	–1.23	–0.11	1.12	6.92	4.35	4.43	3.795	ref 12 **1f**
**1d**	712	561.5	0.060	1.65	–6.156	–1.216	–0.171	1.05	7.60	3.77	4.34	3.714	ref 13 **1d**
**1e**	714	553.9	0.095	1.64	–6.128	–1.197	–0.049	1.15	7.58	3.86	4.31	3.947	ref 13 **1e**
**1f**	709	564.7	0.085	1.67	–6.095	–1.31	–0.19	1.12	7.50	4.09	4.30	3.888	ref 14 **1[5]**
**1g**	704.5	559.9	0.027	1.68	–6.199	–1.29	–0.15	1.14	7.44	4.25	4.25	3.815	ref 12 **1c**
**1h**	676.5	584.5	0.031	1.73	–6.403	–1.24	–0.10	1.14	7.43	4.35	4.35	3.510	ref 12 **1e**
**1i**	823	548.8^h^	0.172^h^	1.46	–6.033	–1.198	–0.185	1.01	7.40	3.99	4.08	3.360	ref 13 **1g**

a The lowest energy absorption band recorded in CH_2_Cl_2_.

b The D_1_ state due mainly (∼70%, expect for **1c** and **1g**, ∼50%) to the β-HOMO → β-LUMO transition, unless stated otherwise, obtained at the TD UCAM-B3LYP/6–31++G­(2d,p)//UB3LYP/6–31G­(2d,p) level of theory in CH_2_Cl_2_ dielectric medium.

c Optical bandgap as the absorption onset.

d Potentials vs Fc/Fc^+^ couple. Recorded in CH_2_Cl_2_ with [Bu_4_N]^+^ [PF_6_]^−^ (50 mM), at ca. 20 °C, 50 mV s^–1^, glassy carbon working electrode.

e *E*_cell_ = *E*_1/2_^0/+1^ – *E*_1/2_^–1/0^.

f Obtained from simulation of spectra recorded in benzene at ca. 20 °C with *Easy Spin* using at least five H atoms.

g Radical delocalization value. UCAM-B3LYP/EPR-III//UB3LYP/6–31G­(2d,p) level of theory in benzene dielectric medium.

h D_2_ state due to β-HOMO–1 → β-LUMO (36%) and β-HOMO → β-LUMO (29%) transitions. Low oscillator strength (*f* = 0.003) of the D_1_ state at 676.2 nm. For details, see the .

## SUMMARY AND CONCLUSIONS

Extensive structural analysis of radical *leuco* forms **1-H** revealed that all 8-aryloxybenzo­[*e*]­[1,2,4]­triazines **2** undergo regioselective photocyclization with a Smiles rearrangement, which is contrary to the hypothesis used in refs 12 and 13 for the original structural assignment. The only exception in the series appears to be the 8-phenoxy derivative, which does not form a persistent charge-separated zwitterion in the excited state necessary for the formation of the transient spirooxazole. In the absence of the zwitterionic T_1_ state, the phenoxy derivative undergoes cyclization into the *ortho* position with a low activation energy, as originally postulated.

The NMR method used in this work demonstrates high effectiveness in the unambiguous structural assignment of Blatter radicals.

## Supplementary Material





















## Data Availability

The data underlying this study are available in the published article and its online .

## Notes

Additional synthetic and compounds characterization details, 1D and 2D NMR spectra, and computational results (ZIP)
FAIR data, including the primary NMR FID files, for compound **1a-H** (ZIP)
FAIR data, including the primary NMR FID files, for compound **1b-H** (ZIP)
FAIR data, including the primary NMR FID files, for compound **1c-H** (ZIP)
FAIR data, including the primary NMR FID files, for compound **1d-H** (ZIP)
FAIR data, including the primary NMR FID files, for compound **1e-H** (ZIP)
FAIR data, including the primary NMR FID files, for compound **1f-H** (ZIP)
FAIR data, including the primary NMR FID files, for compound **1g-H** (ZIP)
FAIR data, including the primary NMR FID files, for compound **1h-H** (ZIP)
FAIR data, including the primary NMR FID files, for compound **1i-H** (ZIP)

## ABBREVIATIONS

XRD: X-ray diffraction
DFT: density functional theory
HOMO: highest occupied molecular orbital
LUMO: lowest unoccupied molecular orbital
CV: cyclic voltammetry
TLC: thin layer chromatography
