# Supplementary material for: Photocyclization of 8‑Aryloxybenzo[e][1,2,4]triazines Revisited: Unambiguous Structural Assignment of Planar Blatter Radicals by Correlation NMR Spectroscopy
Source: J Org Chem. 2025 Jun 27;90(27):9425–30. doi: 10.1021/acs.joc.5c00741 (PMC12261333; doi:10.1021/acs.joc.5c00741)
Supplement: Supplementary file 1 [file jo5c00741_si_001.zip › Kaszynski_PhotoRevisit_SI.pdf]

Photocyclization of 8-aryloxybenzo[e][1,2,4]triazines revisited: Unambiguous structural assignment of planar Blatter radicals by correlation NMR spectroscopy

Hemant K. Singh,[§] Sławomir Kaźmierski,[§] and Piotr Kaszyński*[§,‡,#]

[§] *Centre of Molecular and Macromolecular Studies, Polish Academy of Sciences, Sienkiewicza 112, 90-363 Łódź, Poland*

[‡] *Faculty of Chemistry, University of Łódź, 91-403 Łódź, Poland*

[#] *Department of Chemistry, Middle Tennessee State University, Murfreesboro, TN 37130, USA*

Table of contents **Page**

1. Preparation of *leuco* **1[n]-H** ……S2

2. NMR spectra ……S5

3. ^{1}H–^{1}H NMR correlation spectra of **1[n]-H** ……S9

4. Computational details ……S38

   a) *mechanistic investigation of photocyclization of **2*** ...S38

   b) *isotropic Fermi contact coupling constants (hfcc) for radicals **1*** ...S38

   c) *spin delocalization of radicals **1*** ...S41

   d) *electronic excitations* ...S41

   e) *partial output data from TD-DFT calculations for radicals **1*** ...S43

5. Archive for DFT calculations ……S57

6. References ……S64

1. Preparation of *leuco* 1[n]-H

^{1}H NMR spectra were recorded in DMSO-d_6**,** containing a drop of CD_2Cl_2 and D_2O on AV III 500 MHz Bruker NMR spectrometer. Chemical shifts are reported in δ ppm relative to DMSO-d_6 residual peak at δ_{1H} = 2.49 ppm.

Signals at 5.72, 4.75 and below 4.5 ppm in each spectrum are due to the residual protonated solvents (CD_2Cl_2, D_2O, DMSO-d_6) and ascorbic acid. NMR data for **1a-H** and **1f-H** were reported previously[1] and are provided here for completeness of this analysis.

Generation of *leuco* 1-H by reduction of radicals 1. General procedure. Radical **1** (1 equiv.) and ascorbic acid (1.2 equiv.) was taken in a 5 mL of RB flask and added D_2O (1 drop), CD_2Cl_2 (2 drops) and DMSO-d_6 (0.4 mL). After 15 min of stirring, sample the solution was placed in an NMR tube and ^{1}H NMR spectrum was recorded. In some cases, complete reduction takes up to 3 hrs.

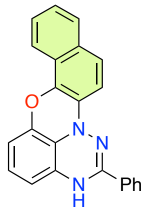

1a-H. ^{1}H NMR (500 MHz, DMSO-d_6) δ 8.87 (s, NH), 7.89 (dd, J_1 = 8.5 Hz, J_2 = 1.1 Hz, 1H), 7.85 (dd, J_1 = 7.5 Hz, J_2 = 1.6 Hz, 2H), 7.77 (d, J = 8.2 Hz, 1H), 7.59 (d, J = 8.9 Hz, 1H), 7.56 (d, J = 8.9 Hz, 1H), 7.52-7.46 (m, 3H), 7.46 (td, J_1 = 6.7 Hz, J_2 = 1.3 Hz, 1H), 7.35 (td, J_1 = 8.1 Hz, J_2 = 1.2 Hz, 1H), 6.76 (t, J = 8.1 Hz, 1H), 6.53 (dd, J_1 = 8.3 Hz, J_2 = 1.2 Hz, 1H), 6.39 (dd, J_1 = 7.9 Hz, J_2 = 1.2 Hz, 1H).

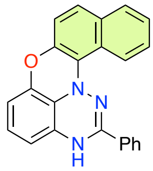

1b-H. ^{1}H NMR (500 MHz, DMSO-d_6) δ 9.33 (s, NH), 8.52 (dd, J_1 = 8.8 Hz, J_2 = 1.2 Hz, 1H), 7.83 (dd, J_1 = 7.8 Hz, J_2 = 1.5 Hz, 2H), 7.80 (d, J = 8.1 Hz, 1H), 7.57 (d, J = 8.8 Hz, 1H), 7.52-7.46 (m, 4H), 7.39 (td, J_1 = 8.1 Hz, J_2 = 1.2 Hz, 1H), 7.08 (d, J = 8.8 Hz, 1H), 6.89 (t, J = 8.0 Hz, 1H), 6.62 (dd, J_1 = 7.9 Hz, J_2 = 1.2 Hz, 1H), 6.54 (dd, J_1 = 8.2 Hz, J_2 = 1.1 Hz, 1H).

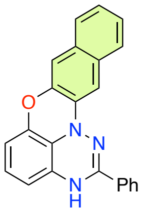

1c-H. ^{1}H NMR (500 MHz, DMSO-d_6) δ 8.80 (s, NH), 7.85 (dd, J_1 = 7.8 Hz, J_2 = 1.4 Hz, 2H), 7.62 (d, J = 8.2 Hz, 1H), 7.56 (d, J = 8.1 Hz, 1H), 7.53-7.46 (m, 3H), 7.35 (s, 1H), 7.24 (td, J_1 = 8.2 Hz, J_2 = 1.4 Hz, 1H), 7.18 (td, J_1 = 8.1 Hz, J_2 = 1.3 Hz, 1H), 7.13 (s, 1H), 6.60 (t, J = 8.2 Hz, 1H), 6.34 (dd, J_1 = 8.4 Hz, J_2 = 1.2 Hz, 1H), 6.24 (dd, J_1 = 7.8 Hz, J_2 = 1.2 Hz, 1H).

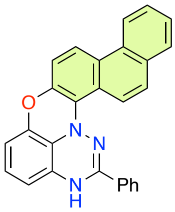

1d-H. ^{1}H NMR (500 MHz, DMSO-d_6) δ 9.39 (s, NH), 8.69 (d, J = 8.3 Hz, 1H), 8.46 (d, J = 9.1 Hz, 1H), 8.41 (d, J = 9.4 Hz, 1H), 7.93 (dd, J_1 = 7.9 Hz, J_2 = 1.5 Hz, 1H), 7.85 (dd, J_1 = 7.8 Hz, J_2 = 1.5 Hz, 2H), 7.79 (d, J = 9.4 Hz, 1H), 7.65 (td, J_1 = 8.4 Hz, J_2 = 1.5 Hz, 1H), 7.60 (td, J_1 = 7.9 Hz, J_2 = 1.2 Hz, 1H), 7.53-7.48 (m, 3H), 7.22 (d, J = 9.0 Hz, 1H), 6.91 (t, J = 8.1 Hz, 1H), 6.64 (dd, J_1 = 7.9 Hz, J_2 = 1.1 Hz, 1H), 6.58 (dd, J_1 = 8.2 Hz, J_2 = 1.1 Hz, 1H).

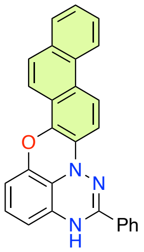

1e-H. ^{1}H NMR (500 MHz, DMSO-d_6) δ 8.87 (s, NH), 8.64 (d, J = 8.3 Hz, 1H), 8.40 (d, J = 9.0 Hz, 1H), 7.91 (d, J = 9.5 Hz, 2H), 7.86 (dd, J_1 = 7.6 Hz, J_2 = 1.8 Hz, 2H), 7.79 (d, J = 9.2 Hz, 1H), 7.65 (d, J = 8.6 Hz, 1H), 7.63 (t, J = 6.5 Hz, 1H), 7.56 (t, J = 7.5 Hz, 1H), 7.53-7.46 (m, 3H), 6.75 (t, J = 8.1 Hz, 1H), 6.54 (d, J = 8.2 Hz, 1H), 6.38 (d, J = 7.8 Hz, 1H).

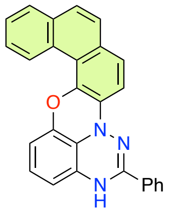

1f-H. ^{1}H NMR (500 MHz, DMSO-d_6) δ 9.42 (d, J = 7.5 Hz, 1H), 7.88 (dd, J_1 = 7.5 Hz, J_2 = 2.0 Hz, 1H), 7.85 (dd, J_1 = 7.6 Hz, J_2 = 1.5 Hz, 2H), 7.66-7.58 (m, 6H), 7.53-7.46 (m, 3H), 6.75 (t, J = 8.1 Hz, 1H), 6.71 (dd, J_1 = 8.3 Hz, J_2 = 1.4 Hz, 1H), 6.37 (dd, J_1 = 7.6 Hz, J_2 = 1.4 Hz, 1H).

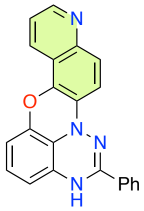

1g-H. ^{1}H NMR (500 MHz, DMSO-d_6) δ 8.92 (s, NH), 8.70 (dd, J_1 = 4.1 Hz, J_2 = 1.6 Hz, 1H), 8.28 (d, J = 8.2 Hz, 1H), 7.85 (dd, J_1 = 7.8 Hz, J_2 = 1.6 Hz, 2H), 7.81 (d, J = 9.2 Hz, 1H), 7.67 (d, J = 9.2 Hz, 1H), 7.53-7.46 (m, 4H), 6.77 (t, J = 8.1 Hz, 1H), 6.51 (dd, J_1 = 8.3 Hz, J_2 = 1.1 Hz, 1H), 6.41 (dd, J_1 = 7.9 Hz, J_2 = 0.5 Hz, 1H).

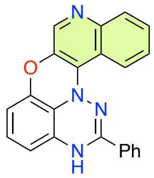

1h-H. ^{1}H NMR (500 MHz, DMSO-d_6) δ 9.03 (s, NH), 8.89 (s, 1H), 7.92 (dd, J_1 = 8.3 Hz, J_2 = 0.9 Hz, 1H), 7.86 (bd, J = 7.7 Hz, 3H), 7.59 (td, J_1 = 8.3 Hz, J_2 = 1.6 Hz, 1H), 7.54 (td, J_1 = 8.2 Hz, J_2 = 1.5 Hz, 1H), 7.53-7.46 (m, 3H), 6.80 (t, J = 8.1 Hz, 1H), 6.58 (dd, J_1 = 8.4 Hz, J_2 = 1.2 Hz, 1H), 6.46 (dd, J_1 = 7.9 Hz, J_2 = 1.1 Hz, 1H).

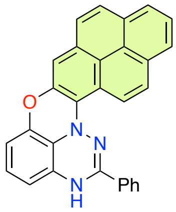

1i-H. ^{1}H NMR (500 MHz, DMSO-d_6) δ 8.76 (d, J = 9.5 Hz, 1H), 8.15 (dd, J_1 = 7.6 Hz, J_2 = 2.3 Hz, 2H), 8.07 (d, J = 9.6 Hz, 1H), 8.00 (d, J = 9.0 Hz, 1H), 7.96 (d, J = 7.6 Hz, 1H), 7.94 (t, J = 6.5 Hz, 1H), 7.92-7.90 (m, 2H), 7.73 (s, 1H), 7.54-7.46 (m, 3H), 6.91 (t, J = 8.0 Hz, 1H), 6.62 (dd, J_1 = 8.0 Hz, J_2 = 1.2 Hz, 1H), 6.61 (dd, J_1 = 8.2 Hz, J_2 = 1.2 Hz, 1H).

1. NMR spectra

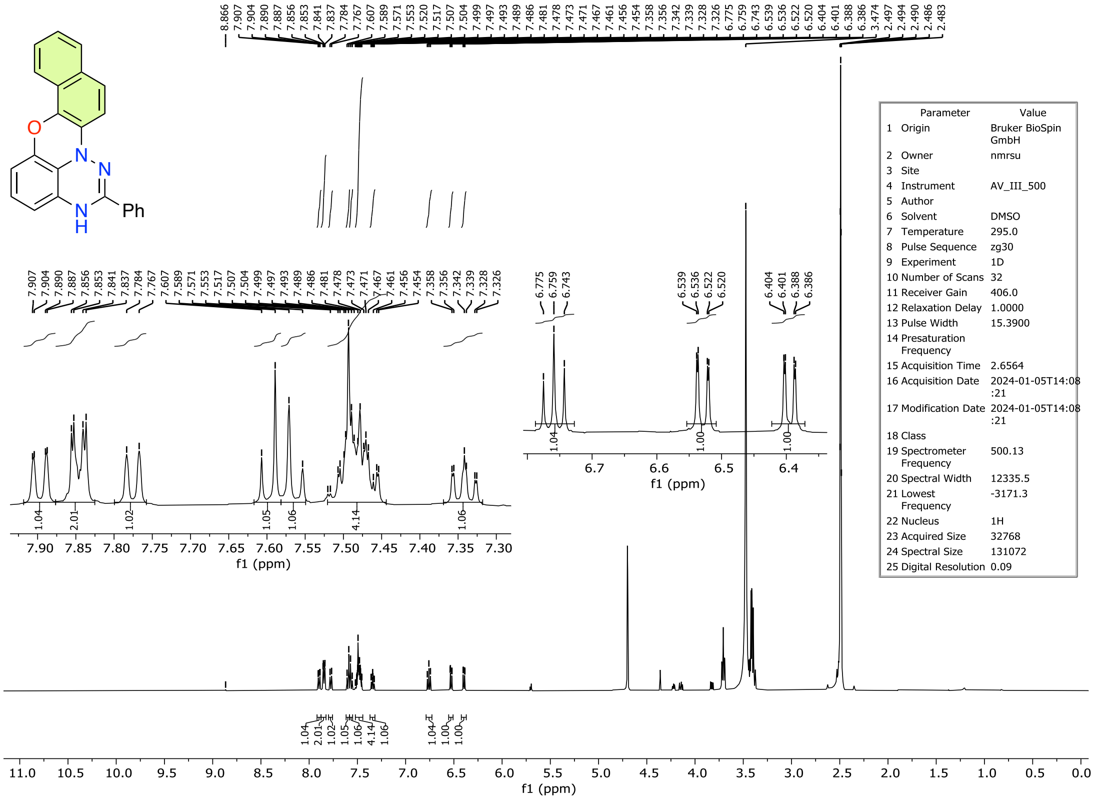

Figure S1. ^{1}H NMR of freshly generated **1a-H** recorded in DMSO-d_6 containing a drop of CD_2Cl_2 and D_2O at 500 MHz. Previously reported in ref[1].

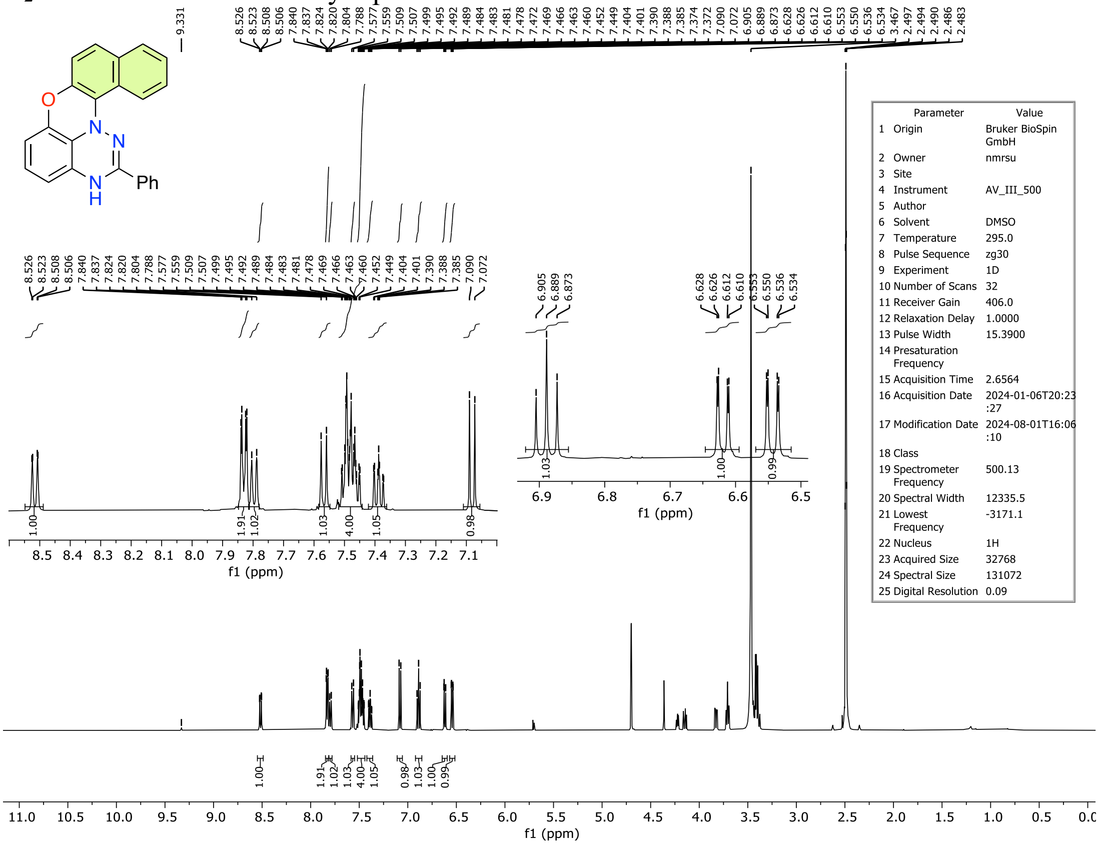

Figure S2. ^{1}H NMR of freshly generated **1b-H** recorded in DMSO-d_6 containing a drop of CD_2Cl_2 and D_2O at 500 MHz.

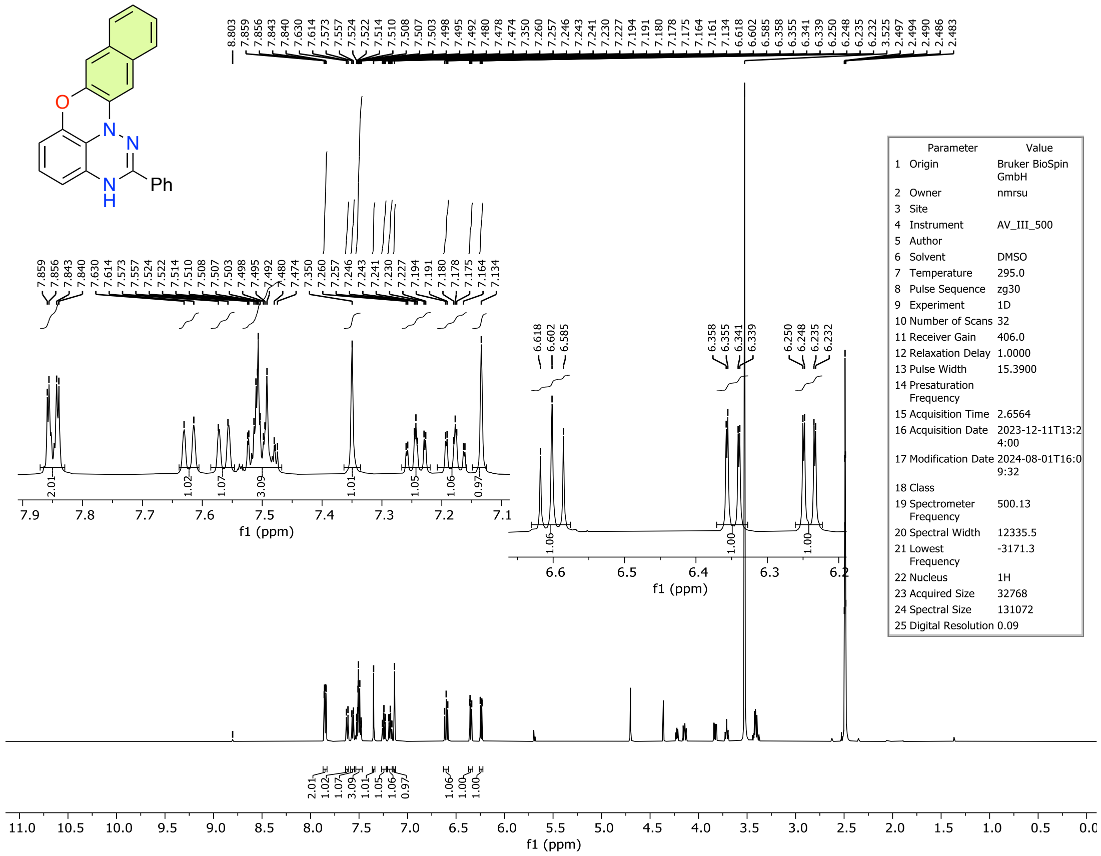

Figure S3. ^{1}H NMR of freshly generated **1c-H** recorded in DMSO-d_6 containing a drop of CD_2Cl_2 and D_2O at 500 MHz.

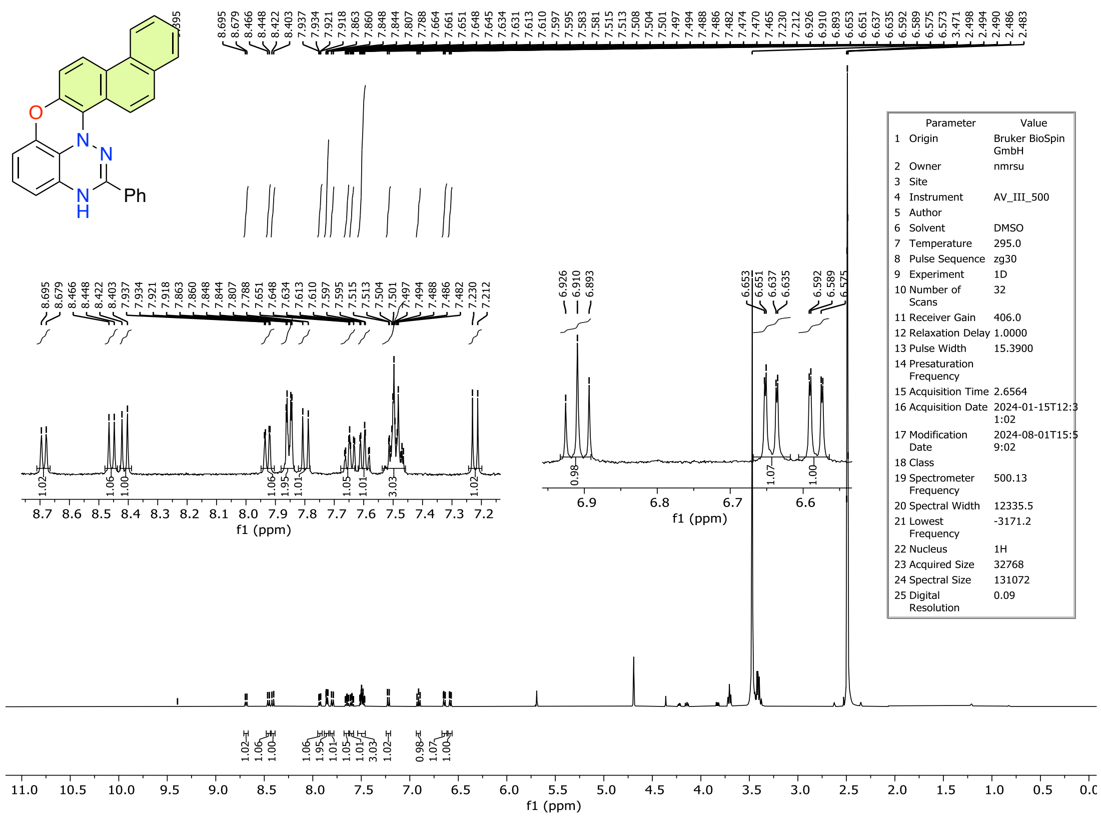

Figure S4. ^{1}H NMR of freshly generated **1d-H** recorded in DMSO-d_6 containing a drop of CD_2Cl_2 and D_2O at 500 MHz.

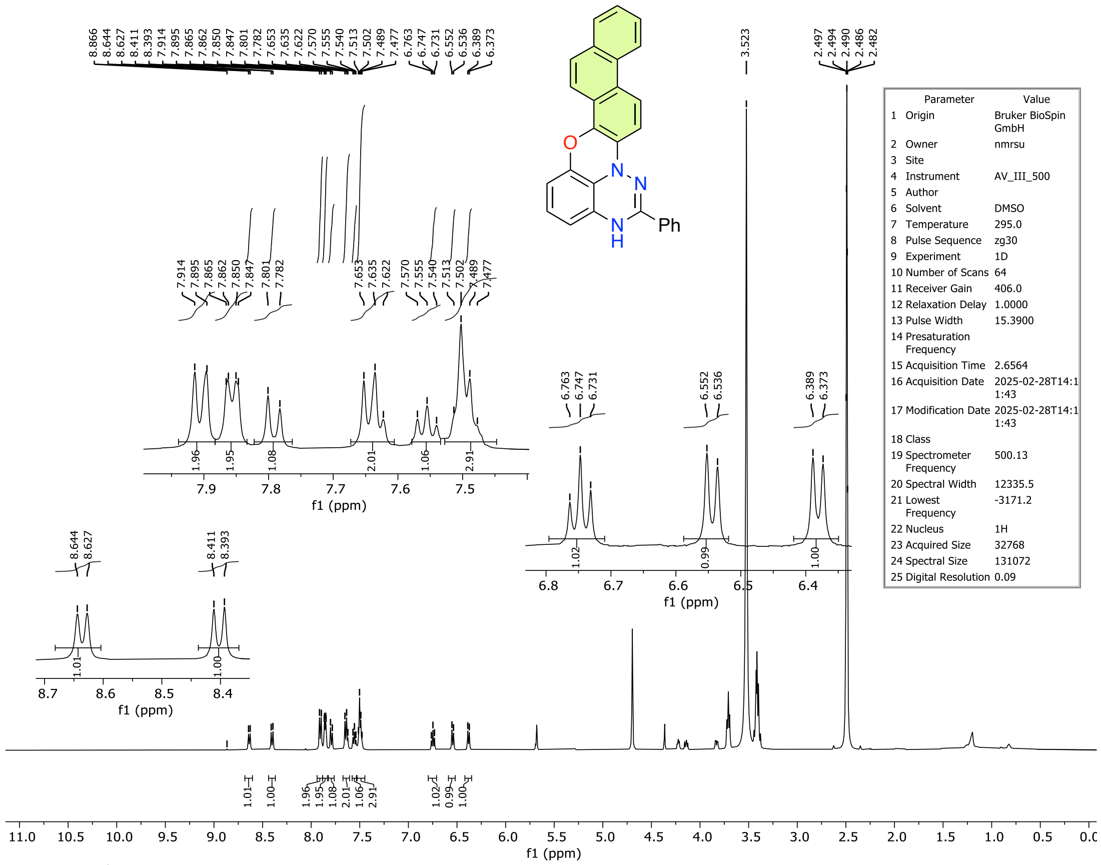

Figure S5. ^{1}H NMR of freshly generated **1e-H** recorded in DMSO-d_6 containing a drop of CD_2Cl_2 and D_2O at 500 MHz.

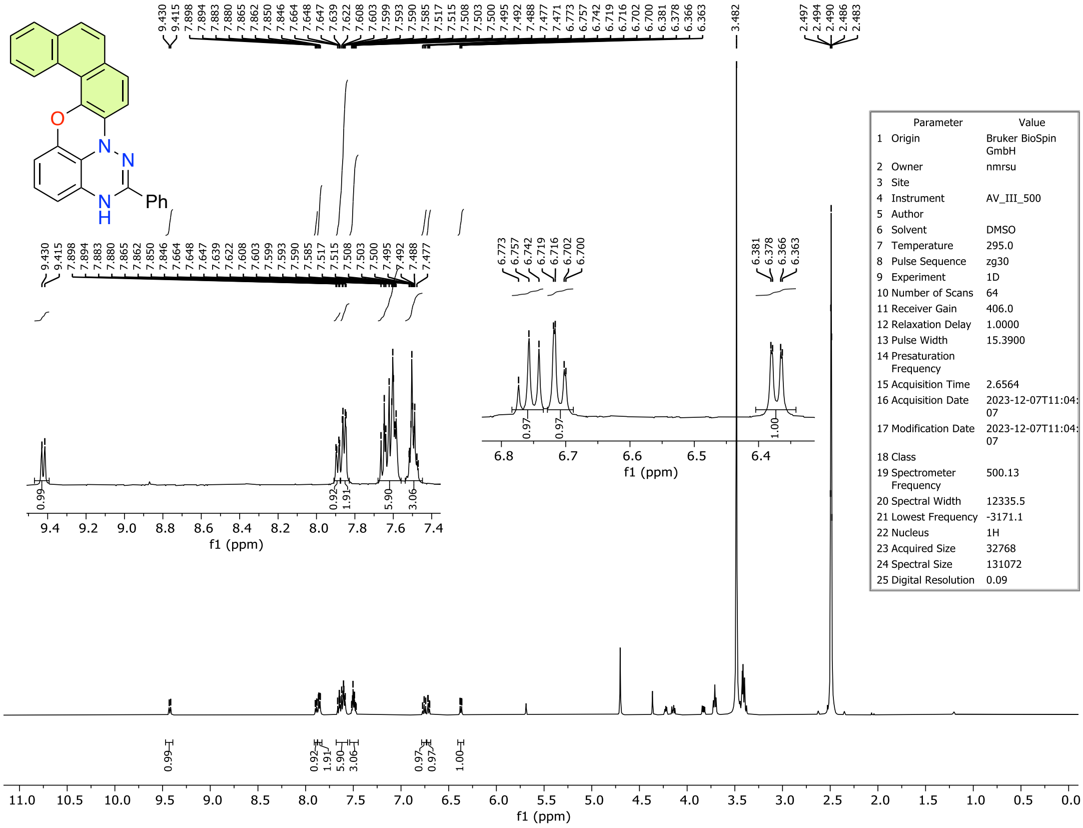

Figure S6. ^{1}H NMR of freshly generated **1f-H** recorded in DMSO-d_6 containing a drop of CD_2Cl_2 and D_2O at 500 MHz. Previously reported in ref[1].

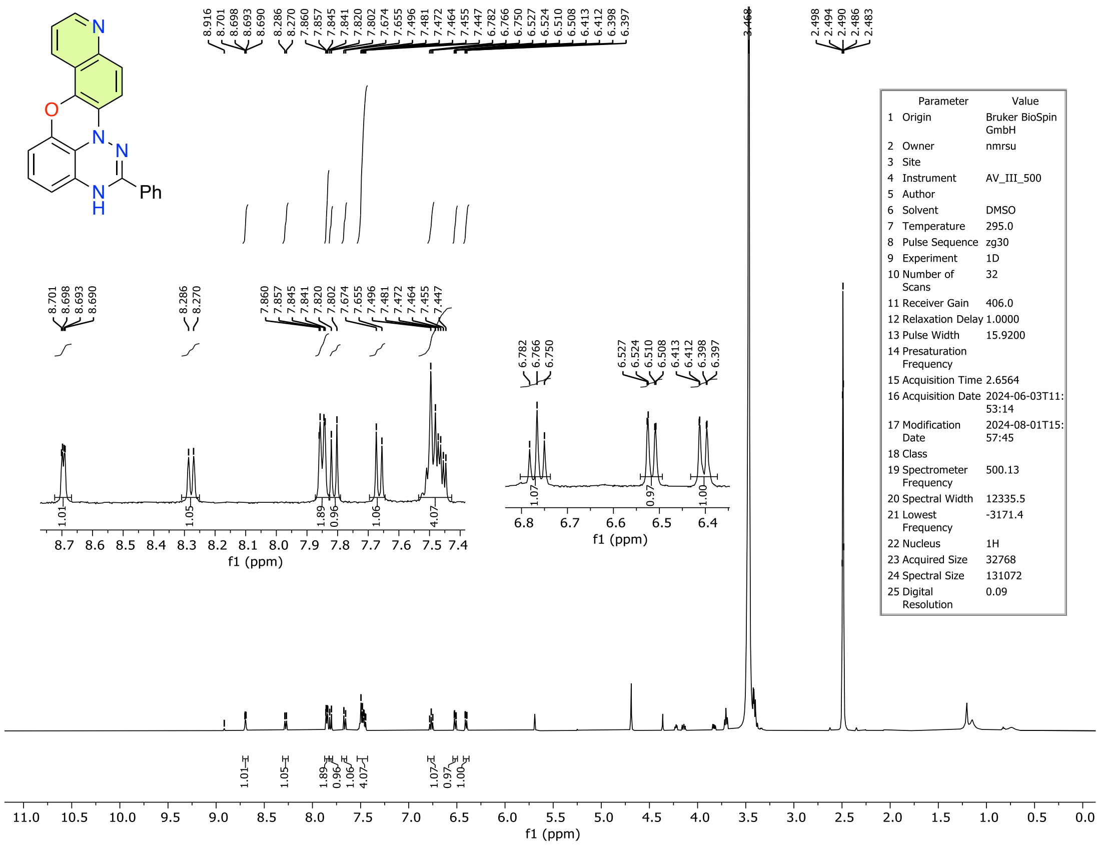

Figure S7. ^{1}H NMR of freshly generated **1g-H** recorded in DMSO-d_6 containing a drop of CD_2Cl_2 and D_2O at 500 MHz.

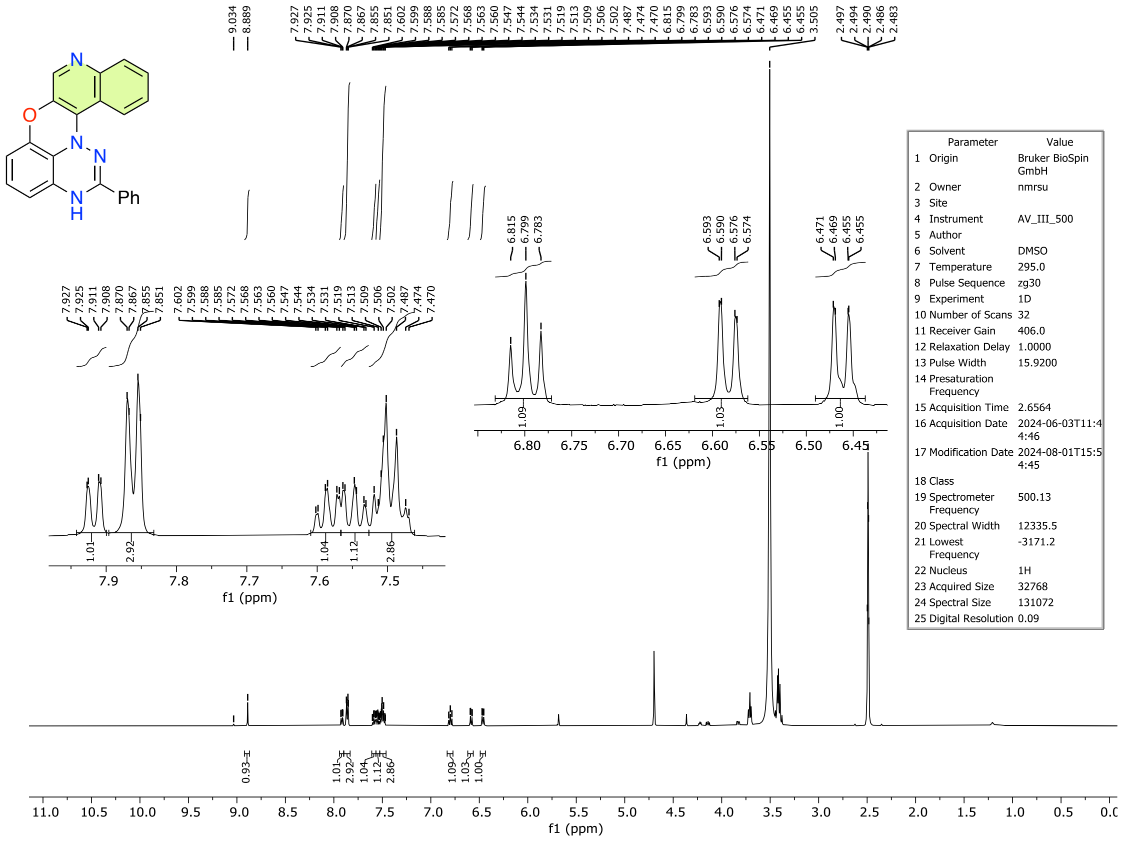

Figure S8. ^{1}H NMR of freshly generated **1h-H** recorded in DMSO-d_6 containing a drop of CD_2Cl_2 and D_2O at 500 MHz.

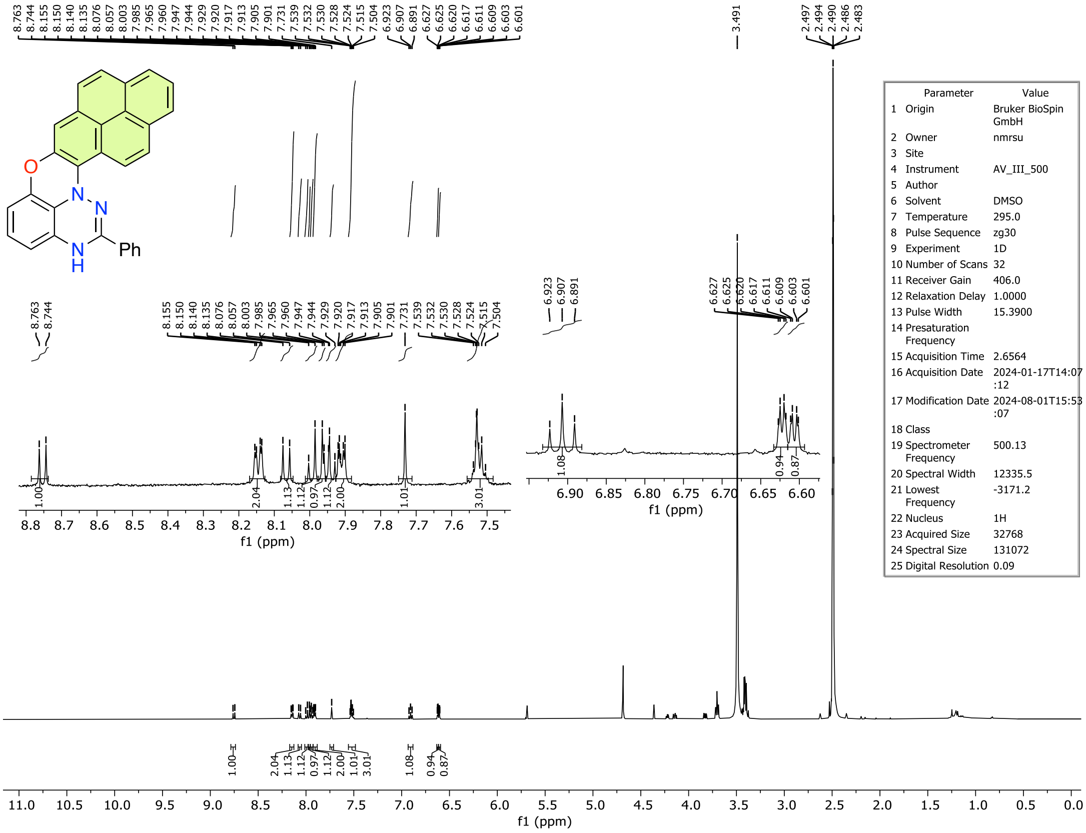

Figure S9. ^{1}H NMR of freshly generated **1i-H** recorded in DMSO-d_6 containing a drop of CD_2Cl_2 and D_2O at 500 MHz.

3. ^{1}H–^{1}H NMR correlation spectra of 1-H

All measurements were performed on Bruker Avance III 500 spectrometer (Bruker BioSpin, Rheinstetten, Germany), operating at frequency of 500.13 MHz for ^{1}H and equipped with GAB/2 gradient unit capable to produce B_0 gradients with maximum strength of 50 G/cm. Automated tuned and matched (ATMA) 5 mm triple channel TBO (BB/H-F/D) probe head with actively shielded Z-gradients coil was utilized. During all measurements, the temperature was controlled and stabilized with BCU 05 cooling unit managed by BVT3200 variable temperature unit. All spectra of **1-H** were recorded in 5 mm NMR tubes using a mixture of deuterated DMSO, CD_2Cl_2 and D_2O solvents. For chemical shift calibration the residual signal of DMSO-d_6 was used (δ_{1H} = 2.49 ppm). For each sample the temperature was stabilized at 295 K for at least 5 minutes and the ^{1}H $\pi/2$ pulse length was checked and corrected before data accumulation. All spectra were acquired, processed and plotted using TopSpin 3.5(pl6) program running on PC computer under Windows 7 Professional.

For 1D ^{1}H spectra 64 scans were accumulated per FID of 64K data points with 1s relaxation delay (D1) and spectral width was set to 12000 Hz (10 ppm) results in 2.64 s of

acquisition time (AQ). Original pulse program zg30 was used. FIDs were zero-filled twice and apodized with LB function of 0.3Hz prior to Fourier transformation.

For 2D COSY, TOCSY and ROESY spectra parameters were as follow: spectra were acquired in 4096 x 512 (F2xF1) data points matrix with 16 (COSY) or 32 (TOCSY, ROESY) scans for each experiment and 32 dummy scans and relaxation delay (D1) of 1.5 s. The spectral width was 5000 Hz (10 ppm) in both dimensions. Prior to Fourier transformation into a final 2048 x 2048 data points matrix, FIDs were apodised with QSINE (2) function in F2 and F1 dimensions. Automatic baseline correction in both dimensions was applied on final 2D spectra. Neither linear prediction nor summarization was applied. Original Bruker pulse programs ***cosygpppqf***, ***mlevph*** and ***roesyphpp.2*** were utilized for COSY, TOCSY[2] and ROESY[3] respectively. TOCSY was run with mixing time (D9) of 120 ms and for ROESY the spin lock time (P15) was set to 350 ms.

The resulting TOCSY and ROESY spectra with indicated structural assignments are shown in Figures S10–S36. A summary of the structural assignment of key ^{1}H NMR signals is shown in Tables 1–3 in the main text.

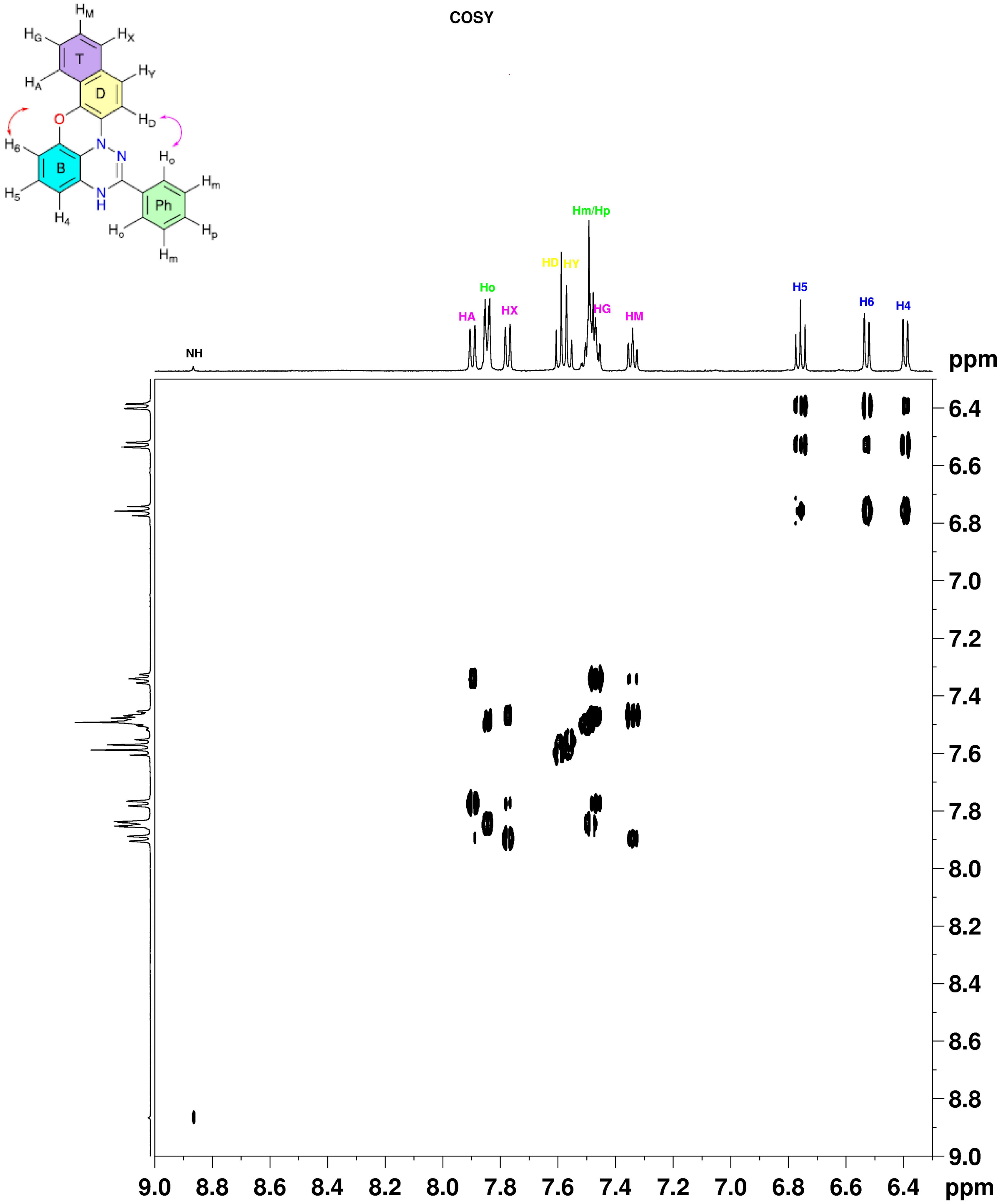

Figure S10. COSY ^{1}H–^{1}H NMR spectra of freshly generated **1a-H** recorded in DMSO-d_6 containing a drop of CD_2Cl_2 and D_2O at 500 MHz.

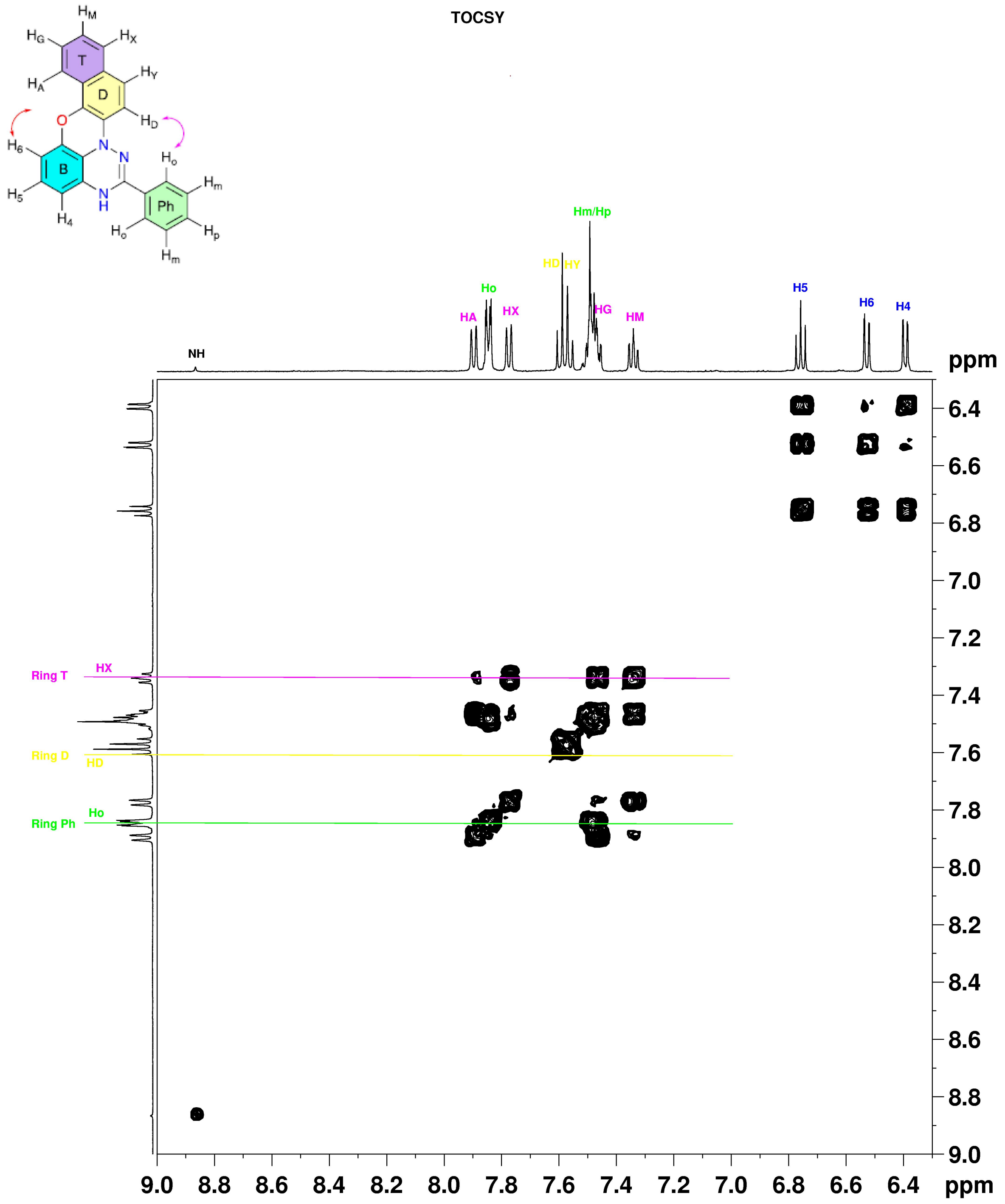

Figure S11. TOCSY ^{1}H–^{1}H NMR spectra of freshly generated **1a-H** recorded in DMSO-d_6 containing a drop of CD_2Cl_2 and D_2O at 500 MHz.

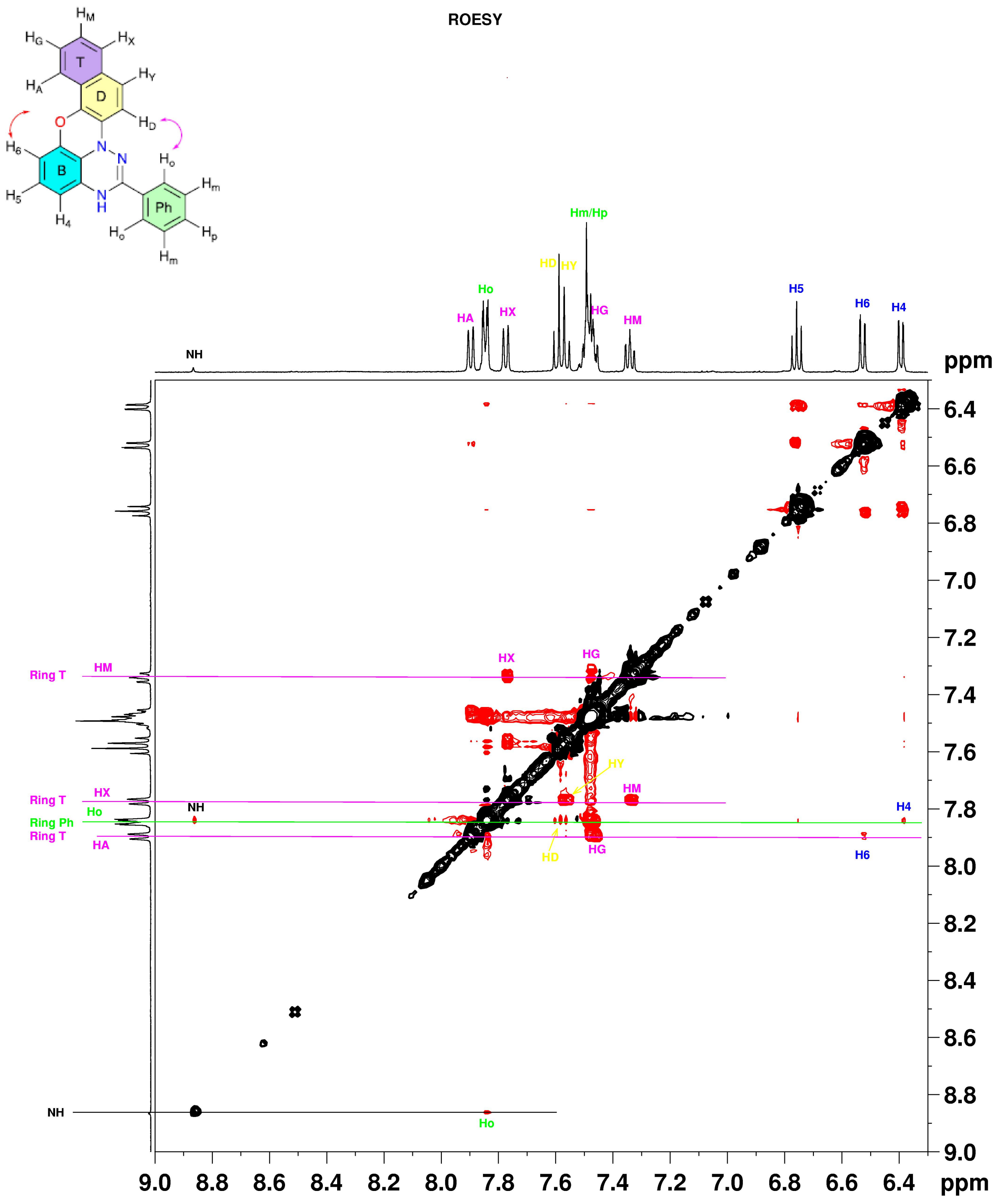

Figure S12. ROESY ^1H–^1H NMR spectra of freshly generated **1a-H** recorded in DMSO-d_6 containing a drop of CD_2Cl_2 and D_2O at 500 MHz.

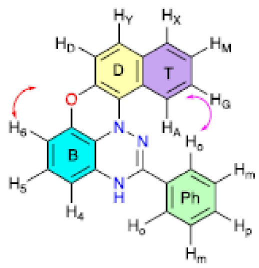

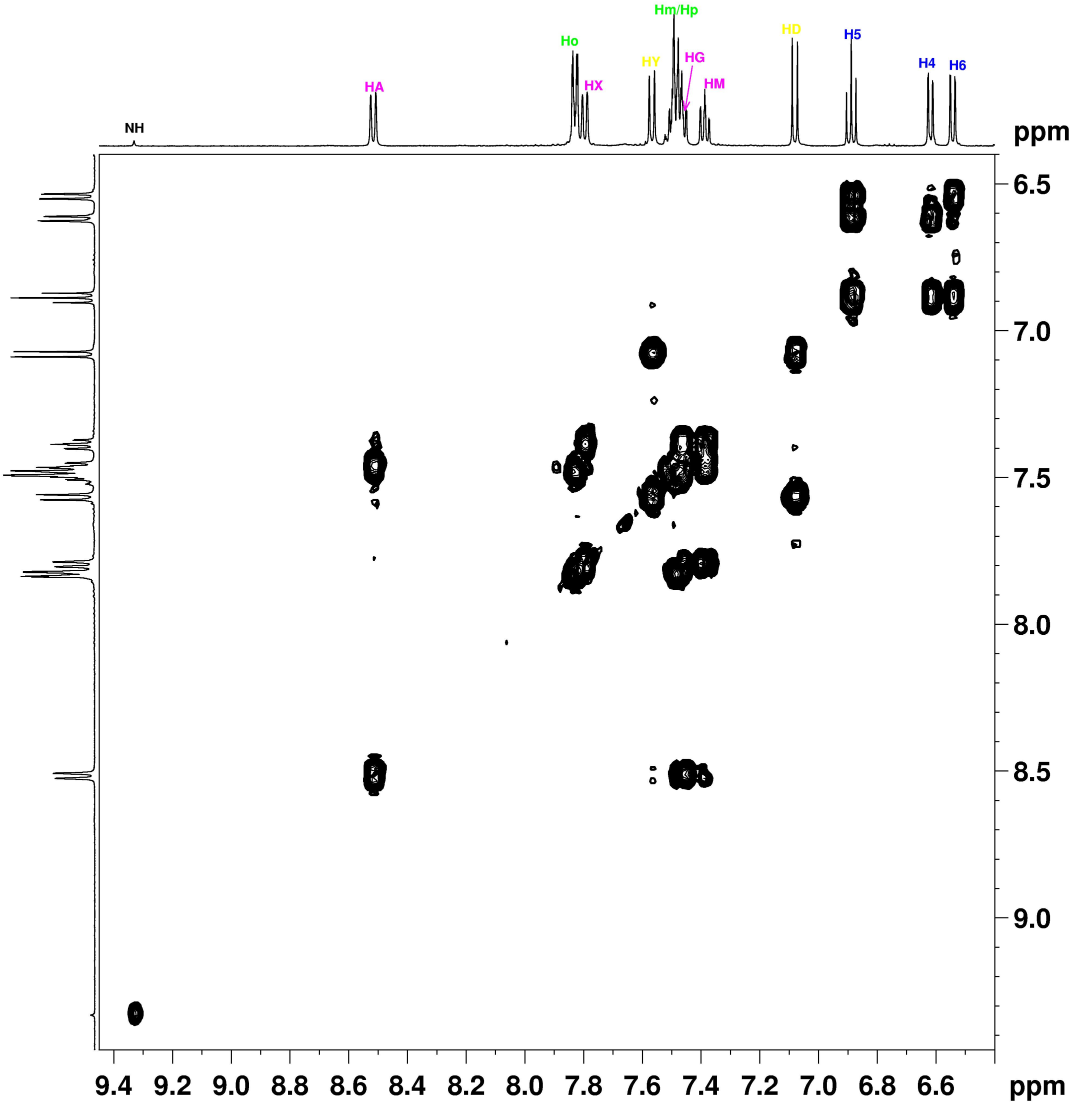

Figure S13. COSY ^{1}H–^{1}H NMR spectra of freshly generated **1b-H** recorded in DMSO-d_6 containing a drop of CD_2Cl_2 and D_2O at 500 MHz.

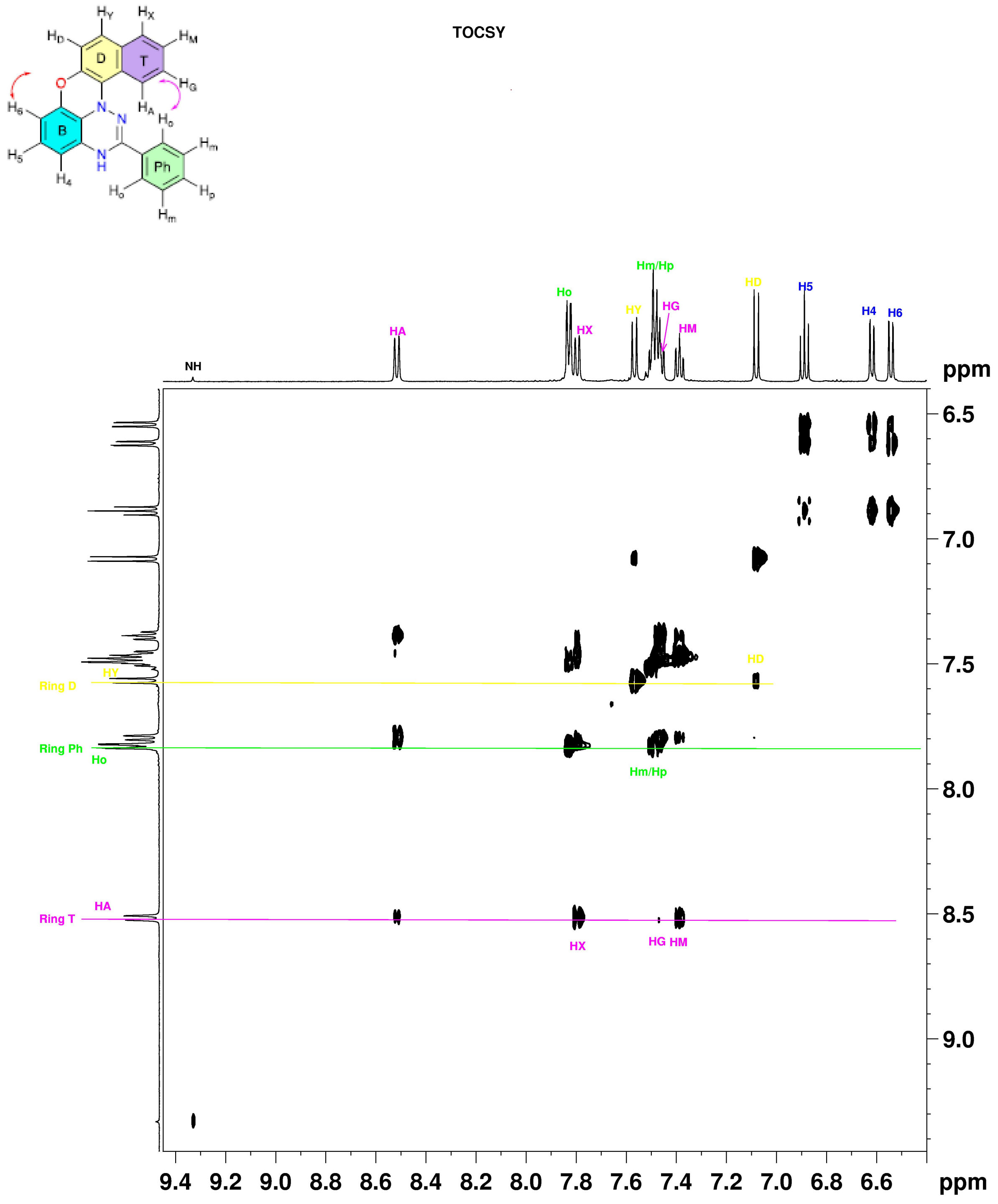

Figure S14. TOCSY ^{1}H–^{1}H NMR spectra of freshly generated **1b-H** recorded in DMSO-d_6 containing a drop of CD_2Cl_2 and D_2O at 500 MHz.

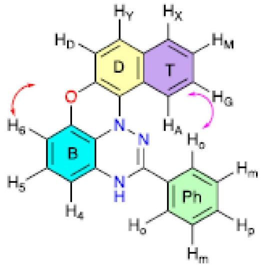

ROESY

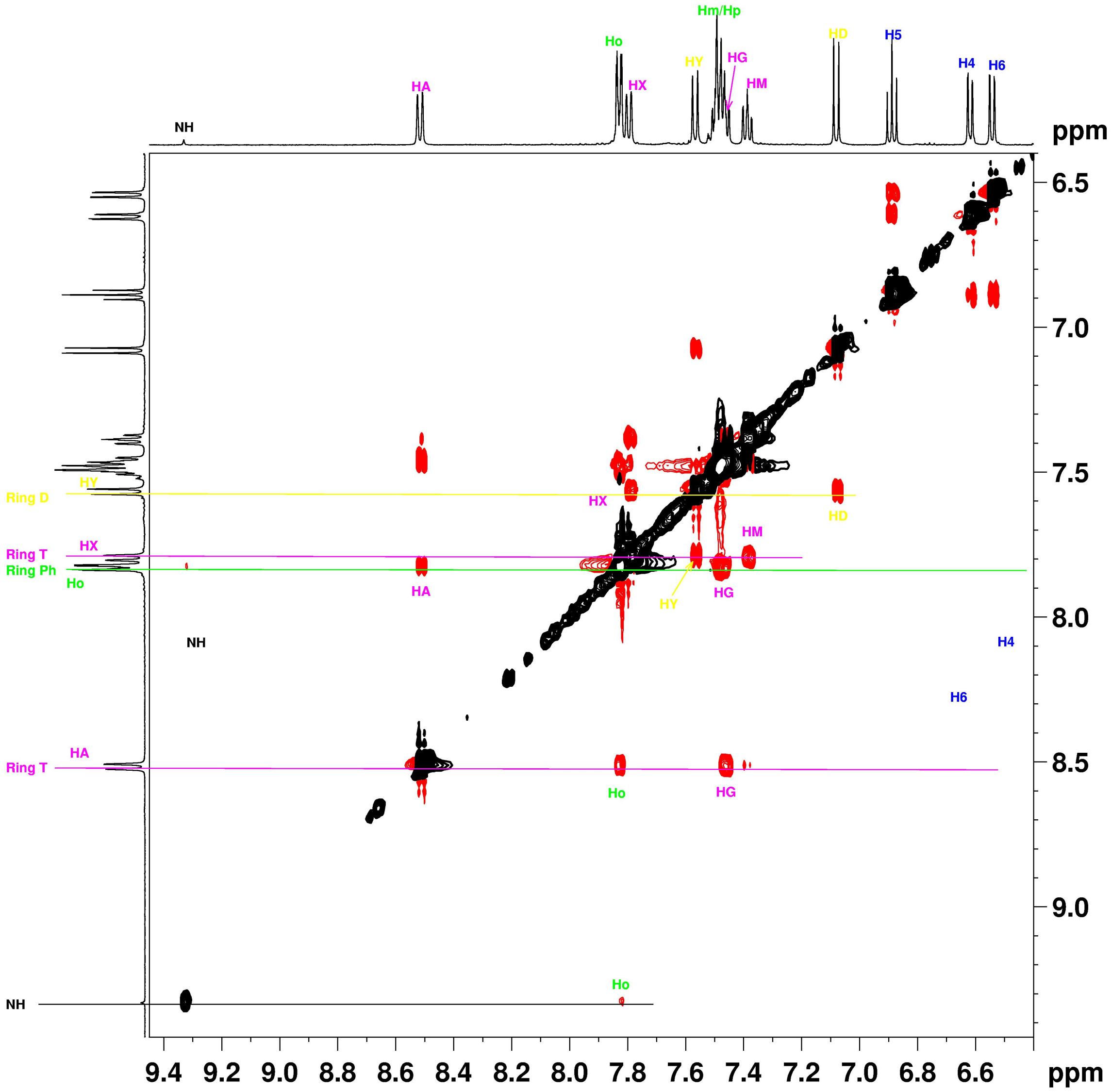

Figure S15. ROESY ^{1}H–^{1}H NMR spectra of freshly generated **1b-H** recorded in DMSO-d_6 containing a drop of CD_2Cl_2 and D_2O at 500 MHz.

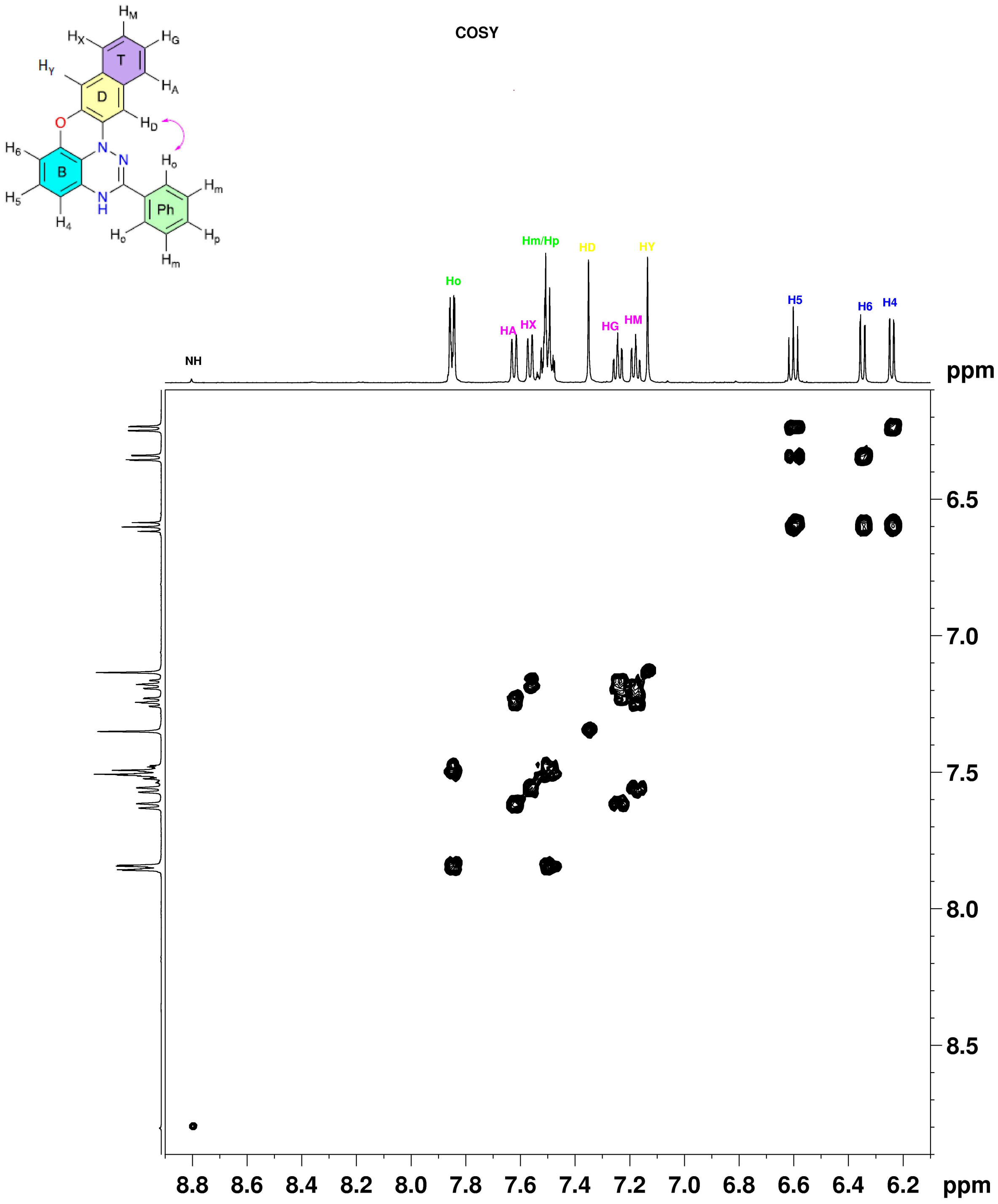

Figure S16. COSY ^{1}H–^{1}H NMR spectra of freshly generated **1c-H** recorded in DMSO-d_6 containing a drop of CD_2Cl_2 and D_2O at 500 MHz.

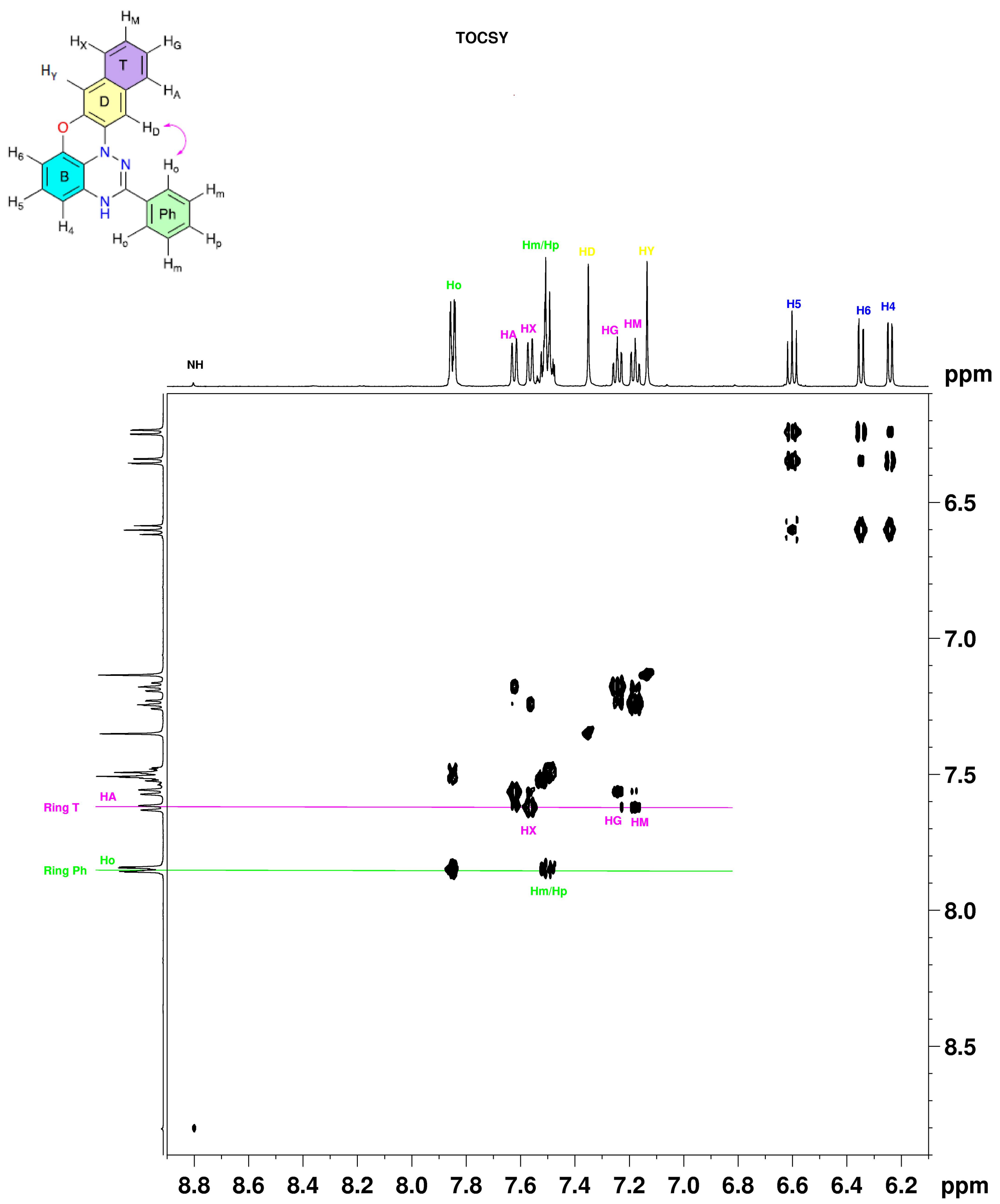

Figure S17. TOCSY ^{1}H–^{1}H NMR spectra of freshly generated **1c-H** recorded in DMSO-d_6 containing a drop of CD_2Cl_2 and D_2O at 500 MHz.

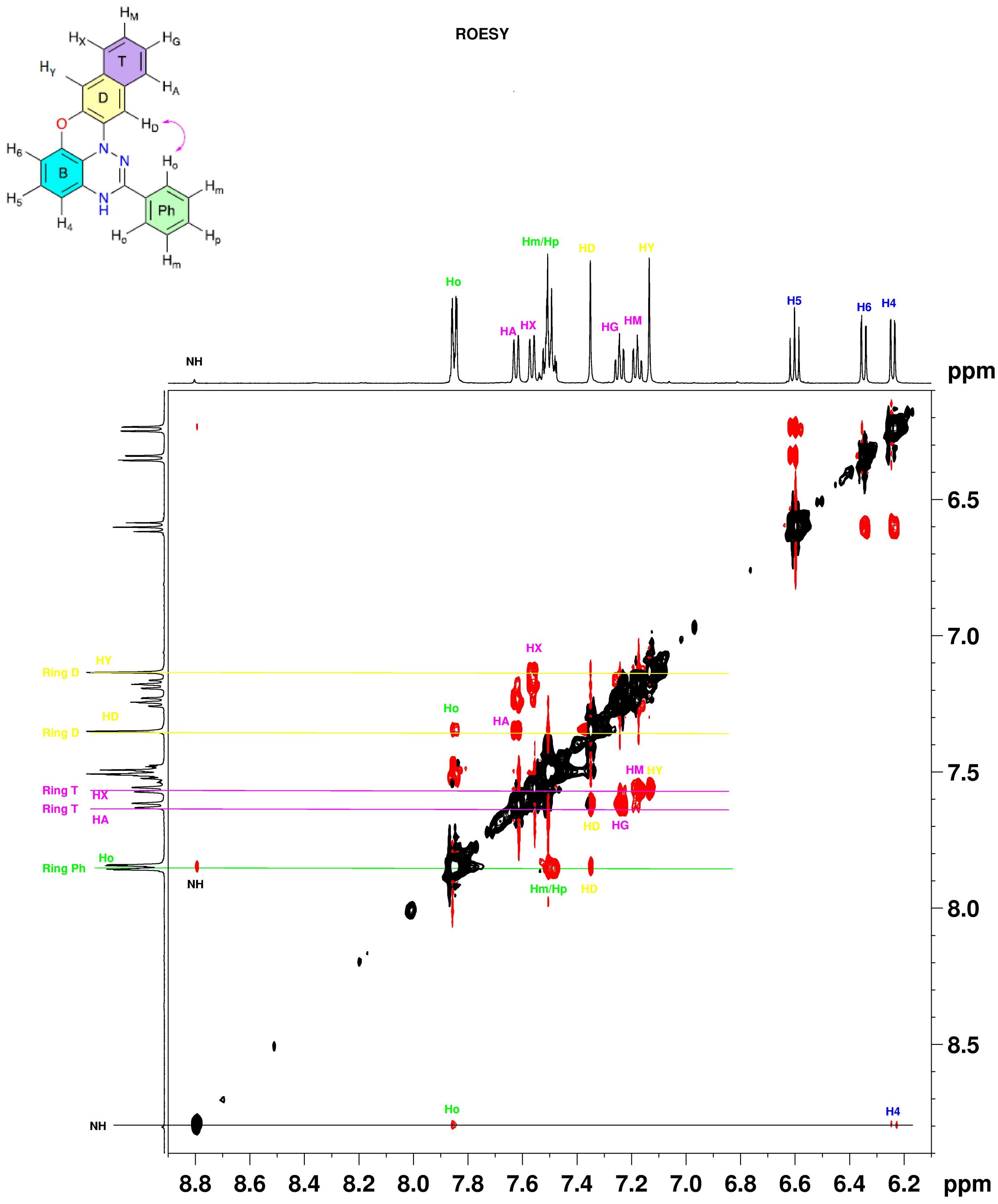

Figure S18. ROESY ^{1}H–^{1}H NMR spectra of freshly generated **1c-H** recorded in DMSO-d_6 containing a drop of CD_2Cl_2 and D_2O at 500 MHz.

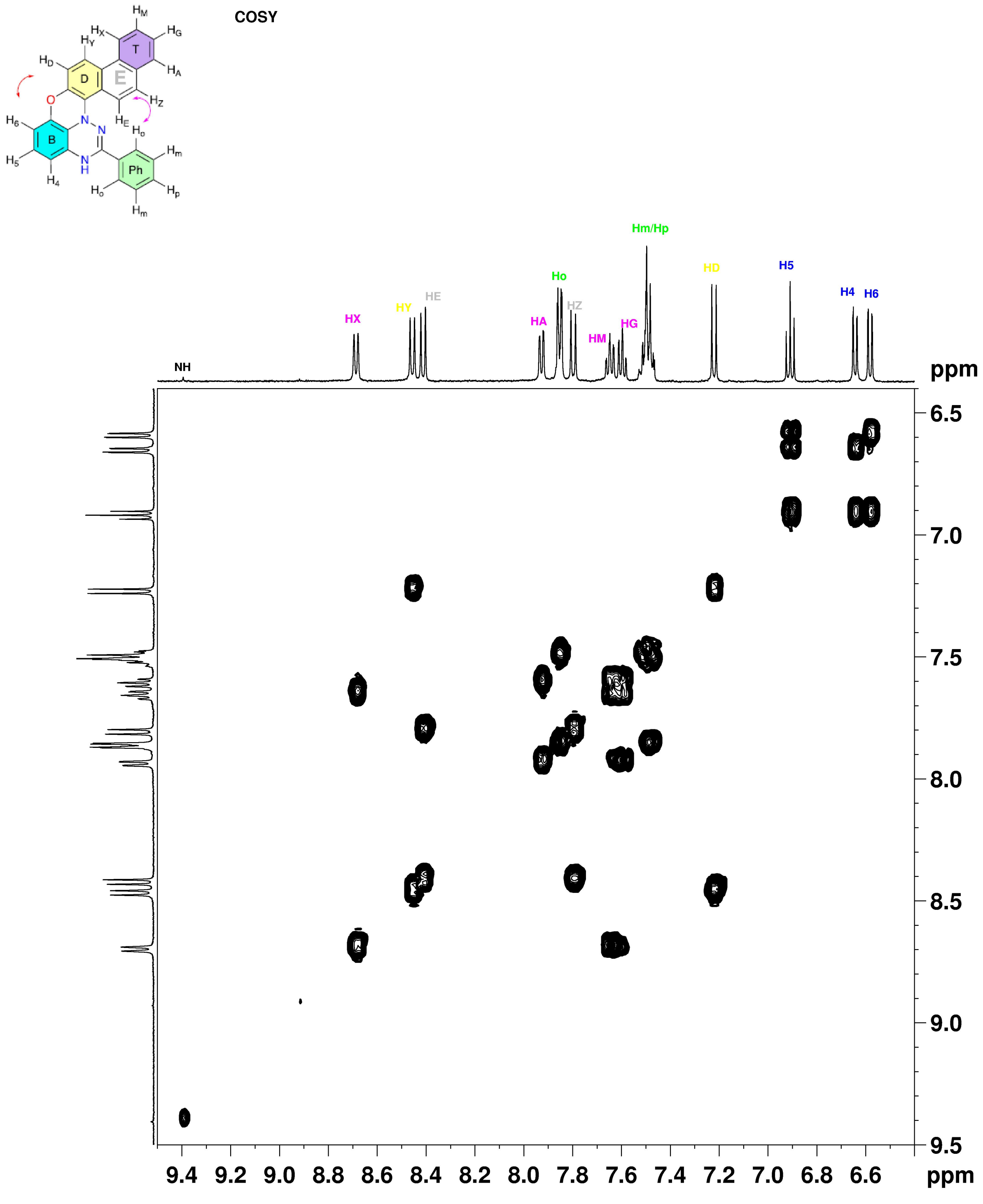

Figure S19. COSY ^{1}H–^{1}H NMR spectra of freshly generated **1d-H** recorded in DMSO-d_6 containing a drop of CD_2Cl_2 and D_2O at 500 MHz.

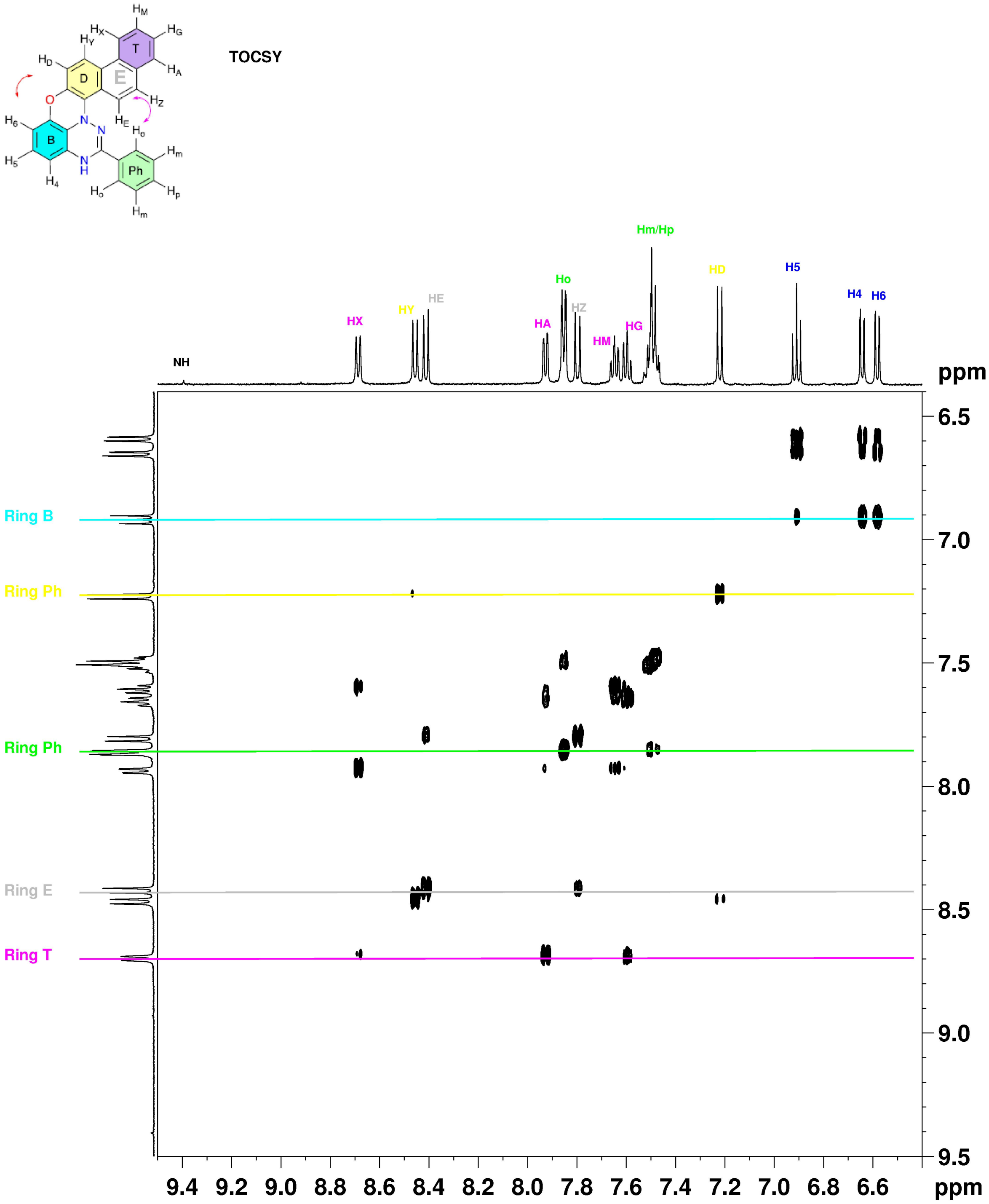

Figure S20. TOCSY ^{1}H–^{1}H NMR spectra of freshly generated **1d-H** recorded in DMSO-d_6 containing a drop of CD_2Cl_2 and D_2O at 500 MHz.

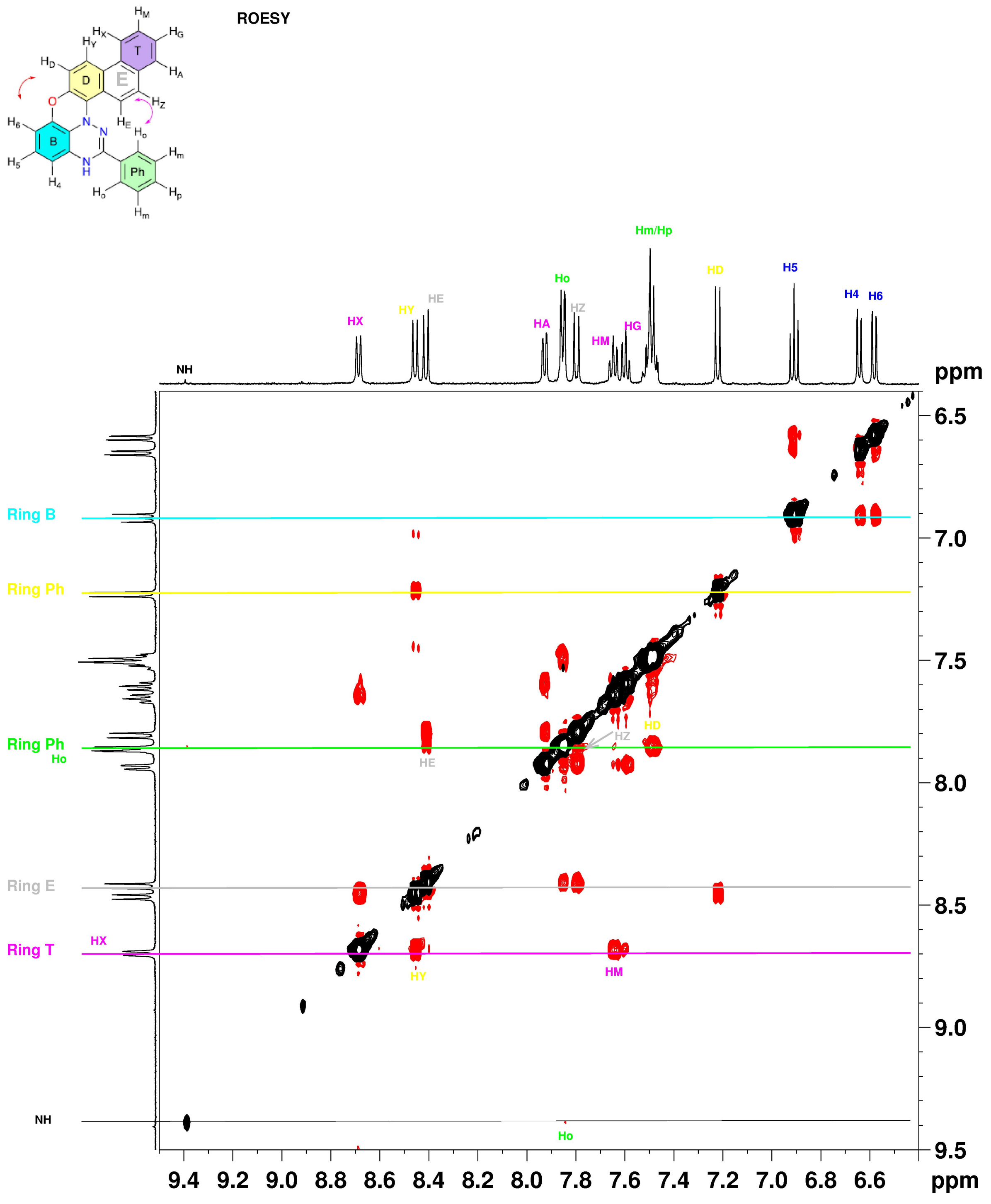

Figure S21. ROESY ^{1}H–^{1}H NMR spectra of freshly generated **1d-H** recorded in DMSO-d_6 containing a drop of CD_2Cl_2 and D_2O at 500 MHz.

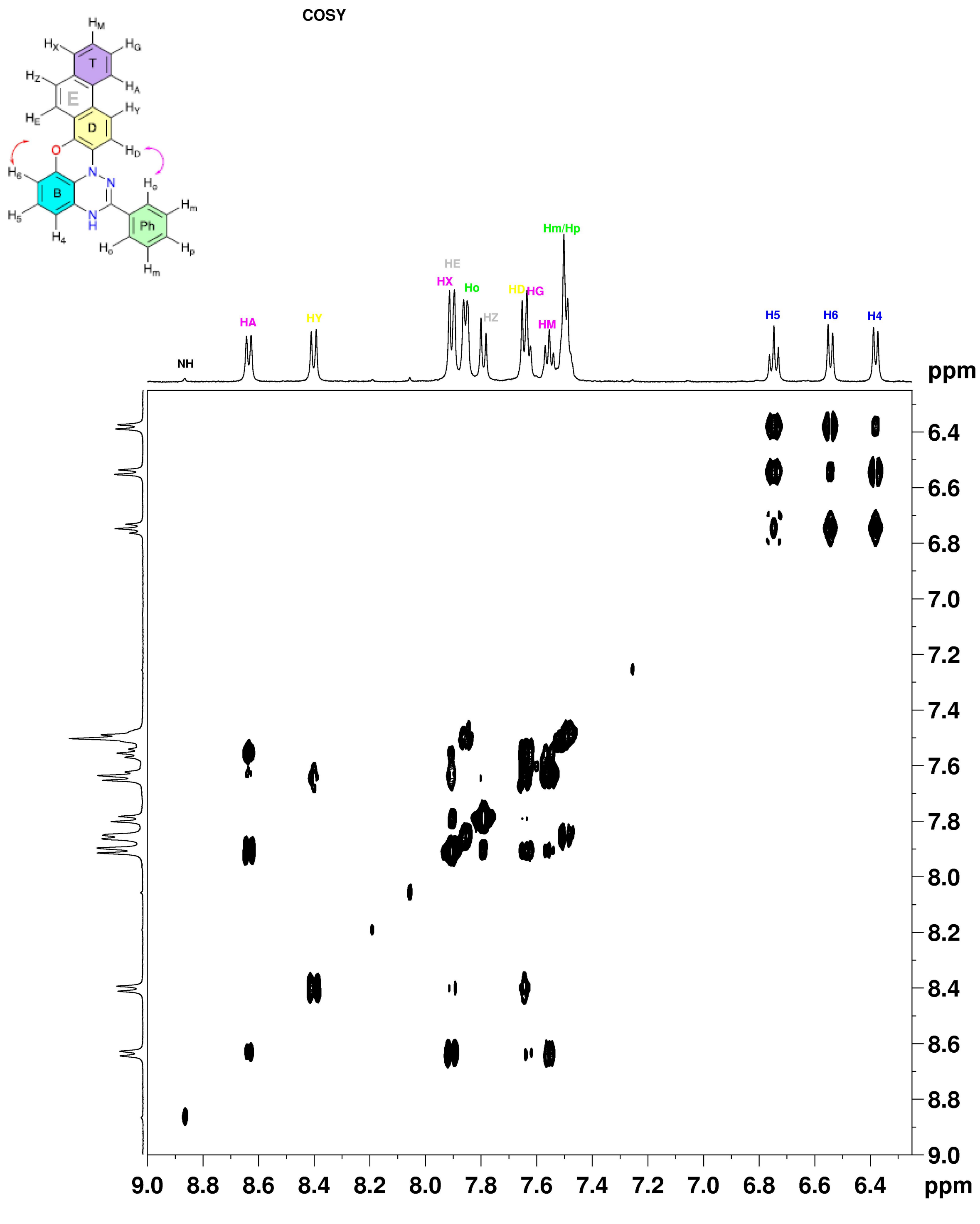

Figure S22. COSY ^1H–^1H NMR spectra of freshly generated **1e-H** recorded in DMSO-d_6 containing a drop of CD_2Cl_2 and D_2O at 500 MHz.

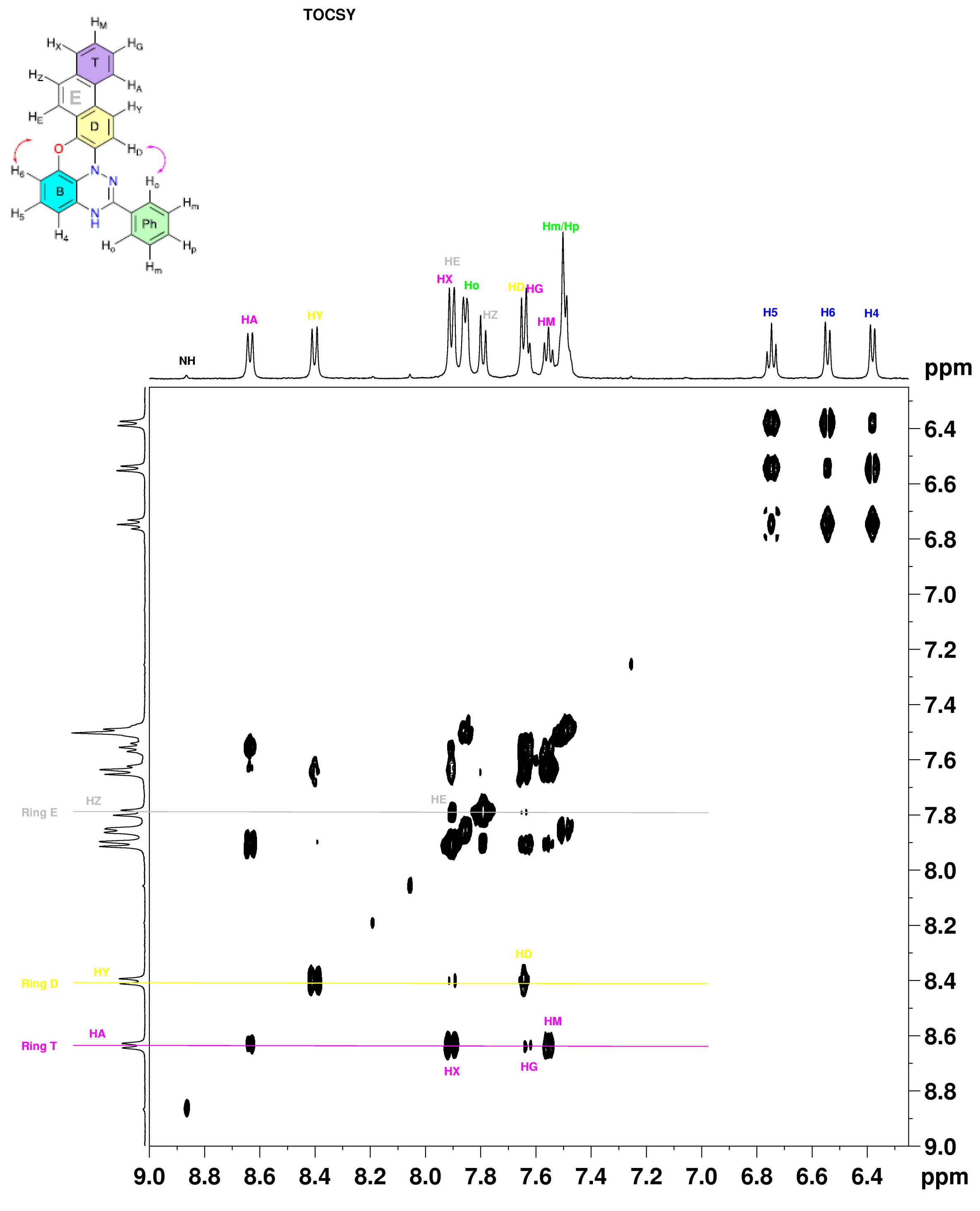

Figure S23. TOCSY ^1H–^1H NMR spectra of freshly generated **1e-H** recorded in DMSO-d_6 containing a drop of CD_2Cl_2 and D_2O at 500 MHz.

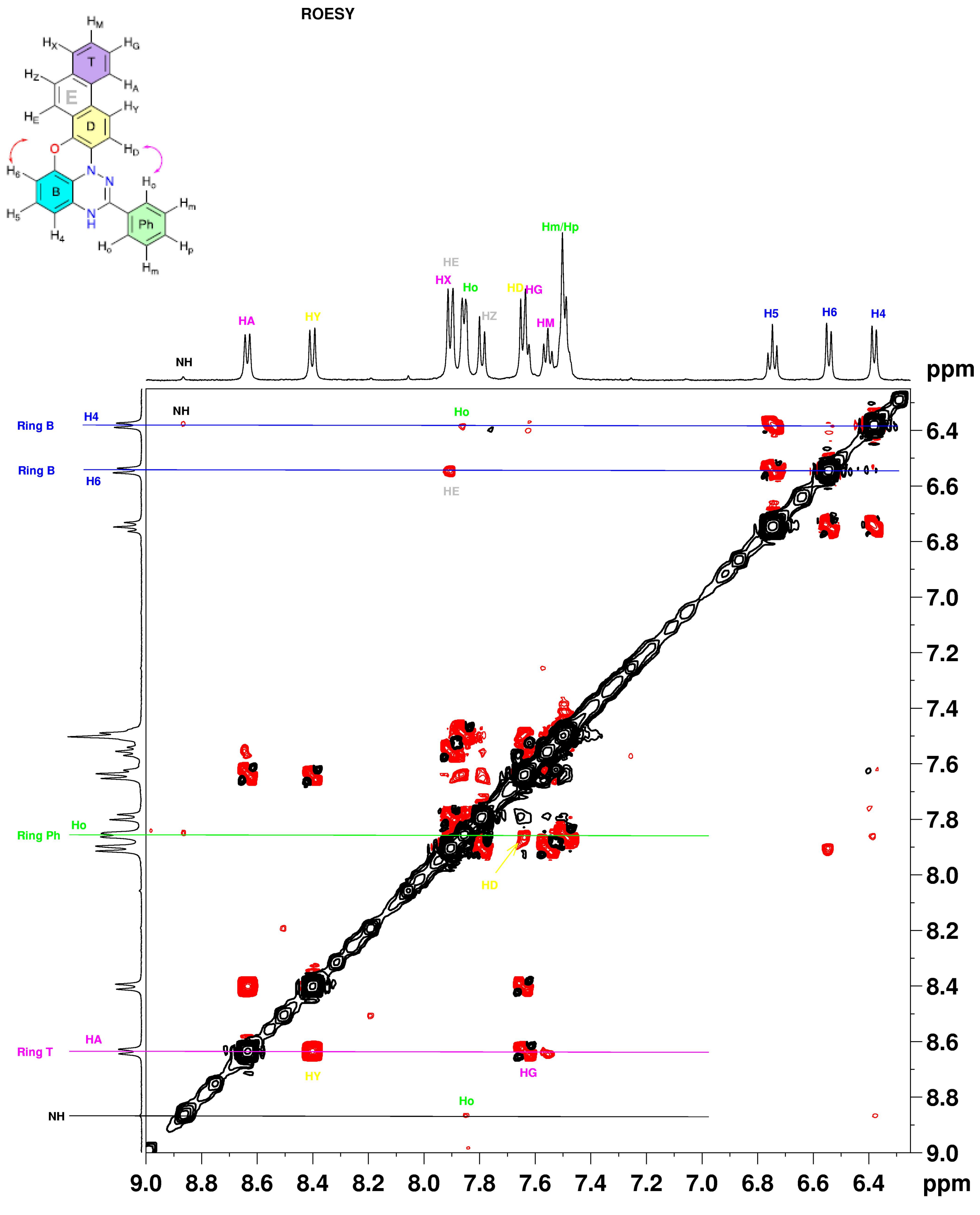

Figure S24. ROESY ^{1}H–^{1}H NMR spectra of freshly generated **1e-H** recorded in DMSO-d_6 containing a drop of CD_2Cl_2 and D_2O at 500 MHz.

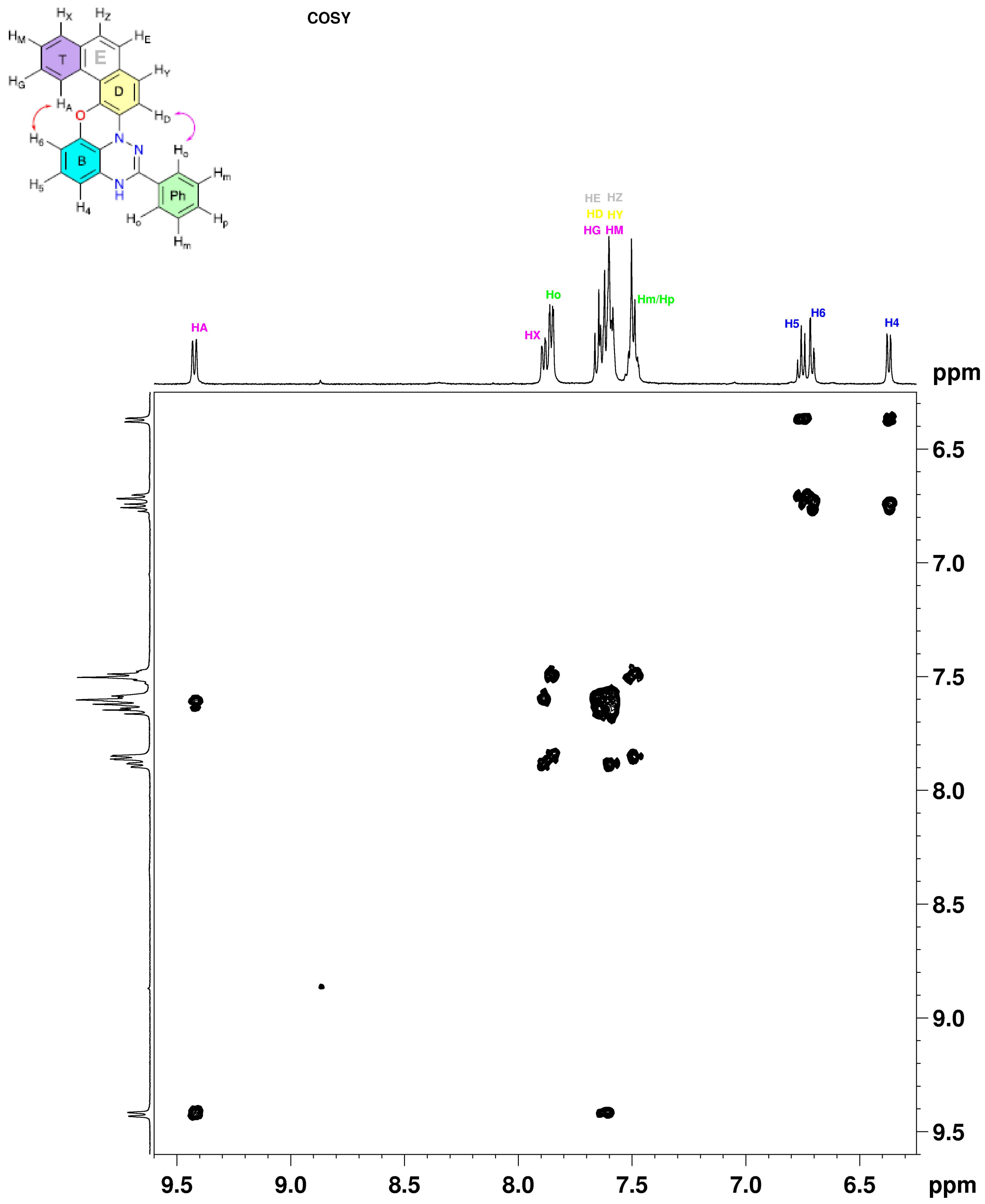

Figure S25. COSY ^{1}H–^{1}H NMR spectra of freshly generated **1f-H** recorded in DMSO-d_6 containing a drop of CD_2Cl_2 and D_2O at 500 MHz.

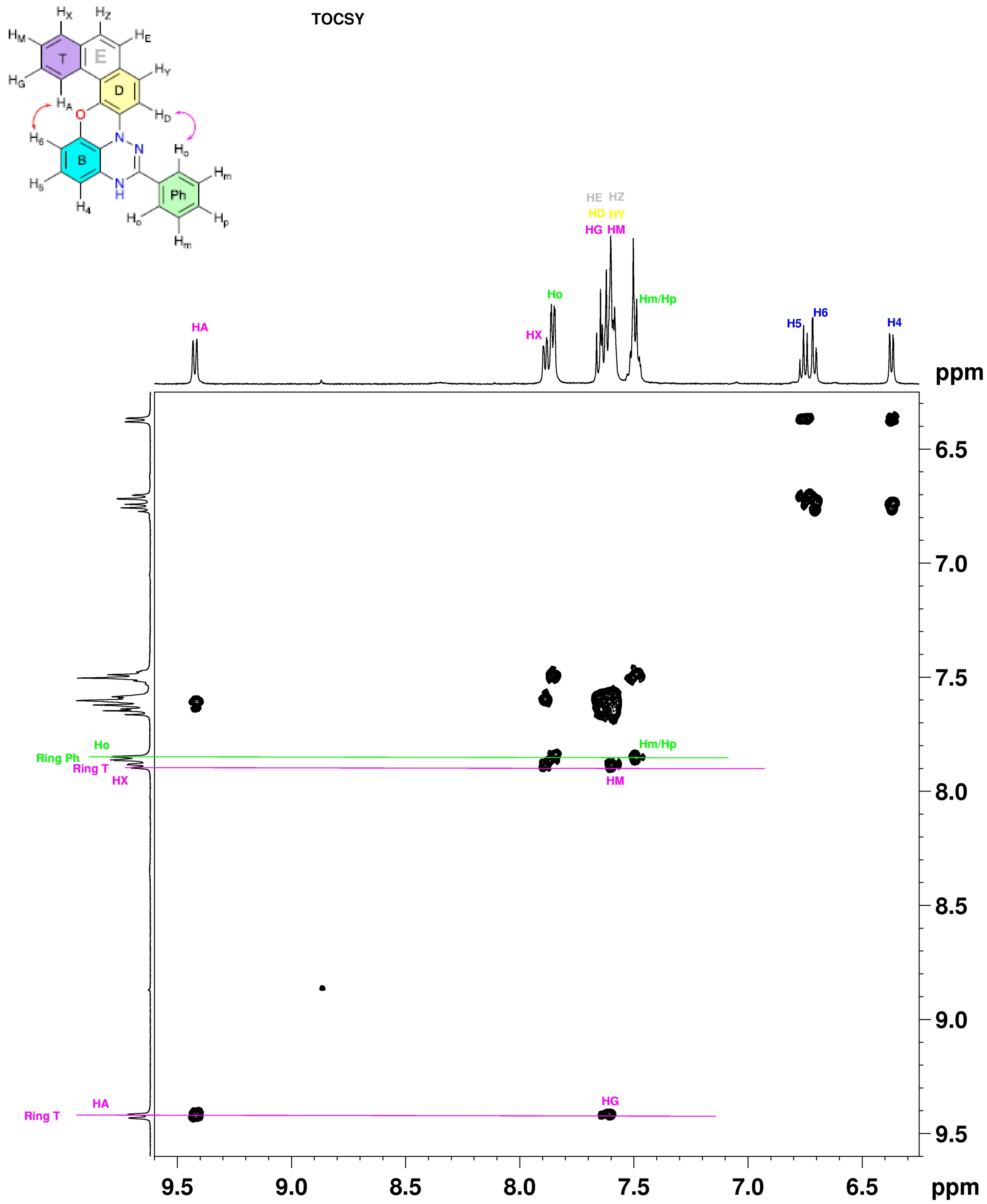

Figure S26. TOCSY ^1H–^1H NMR spectra of freshly generated **1f-H** recorded in DMSO-d_6 containing a drop of CD_2Cl_2 and D_2O at 500 MHz.

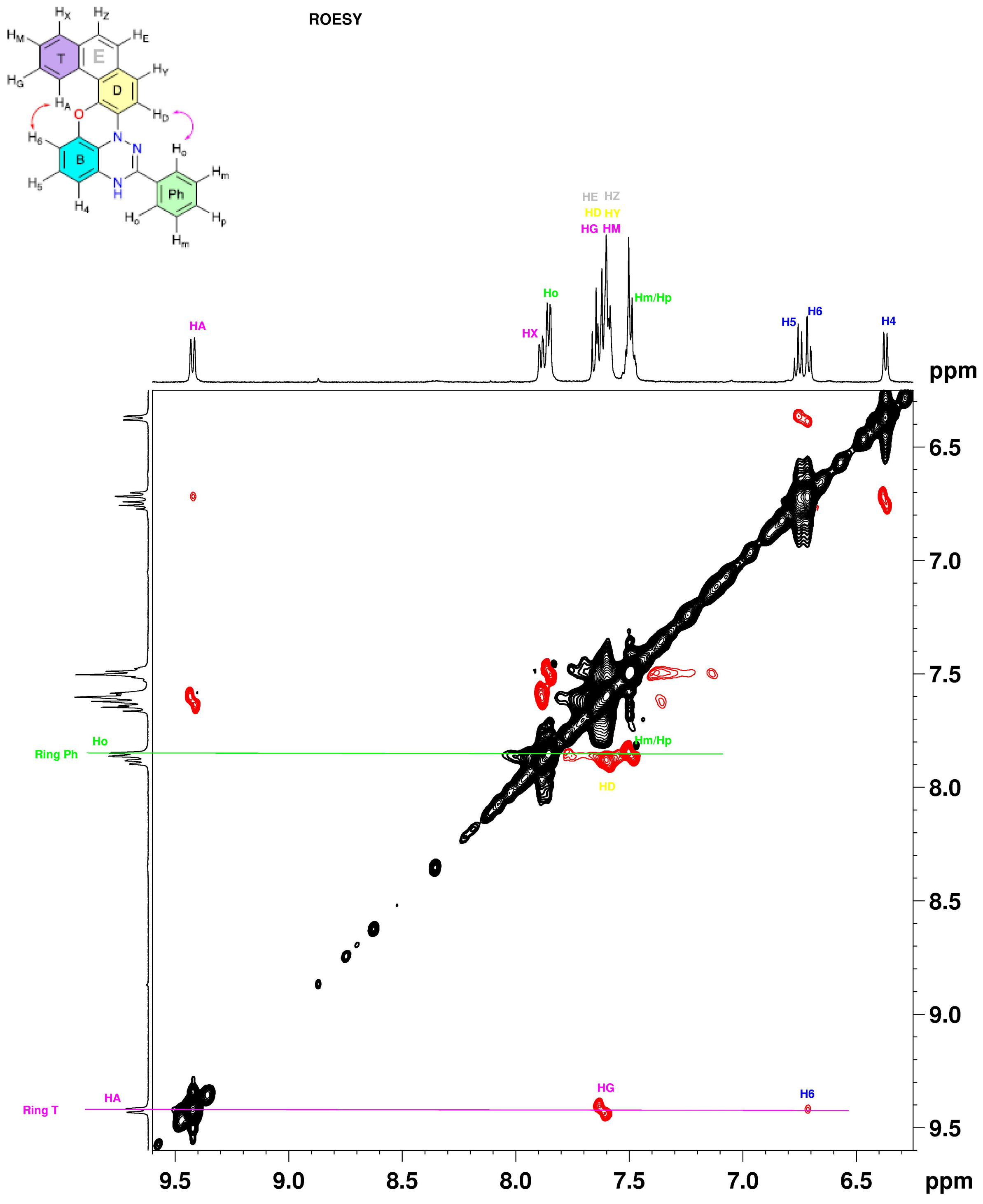

Figure S27. ROESY ^{1}H–^{1}H NMR spectra of freshly generated **1f-H** recorded in DMSO-d_6 containing a drop of CD_2Cl_2 and D_2O at 500 MHz.

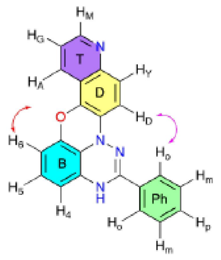

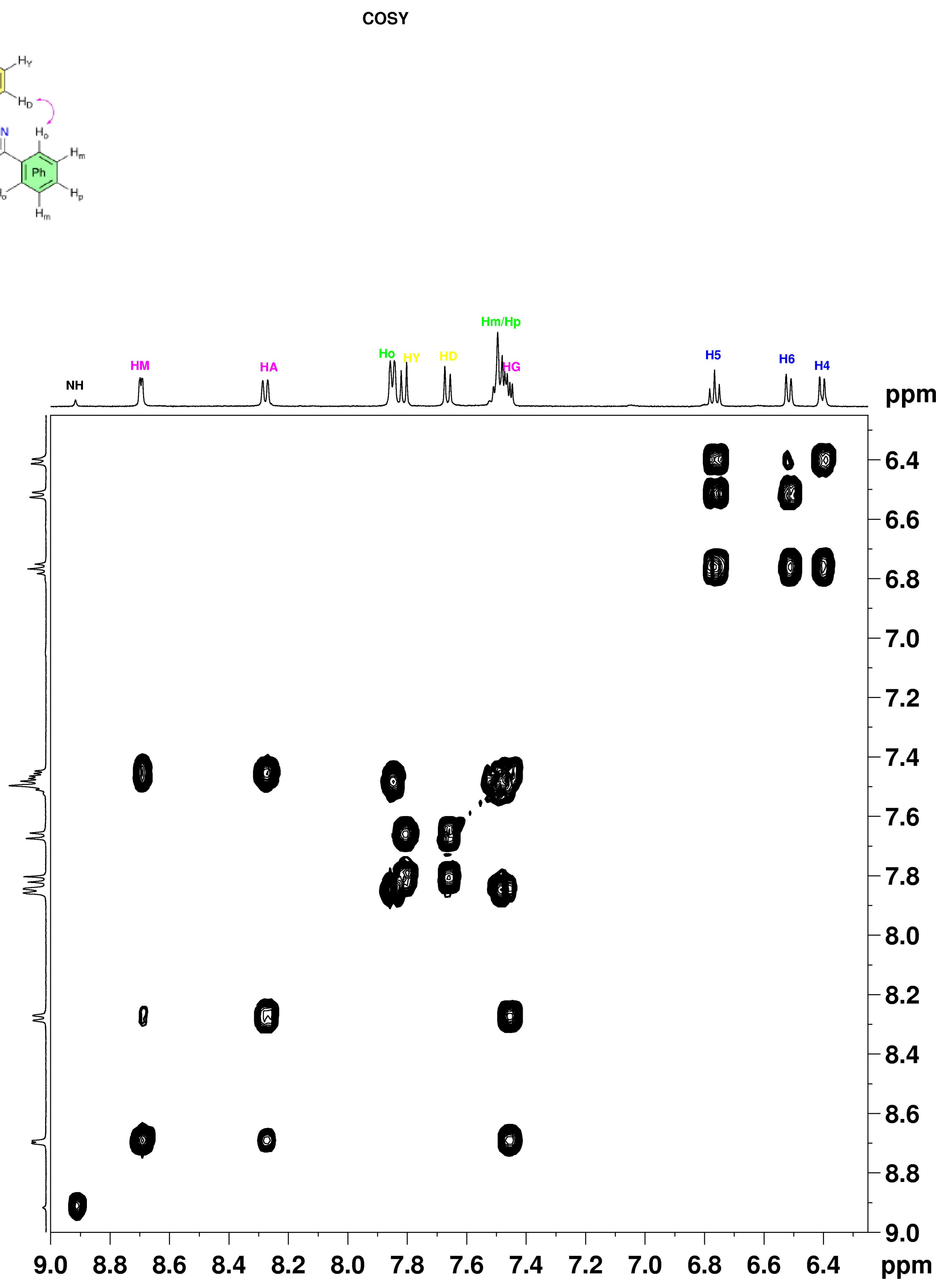

Figure S28. COSY ^{1}H–^{1}H NMR spectra of freshly generated **1g-H** recorded in DMSO-d_6 containing a drop of CD_2Cl_2 and D_2O at 500 MHz.

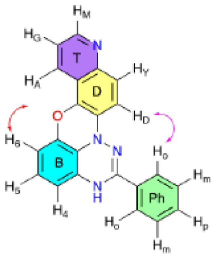

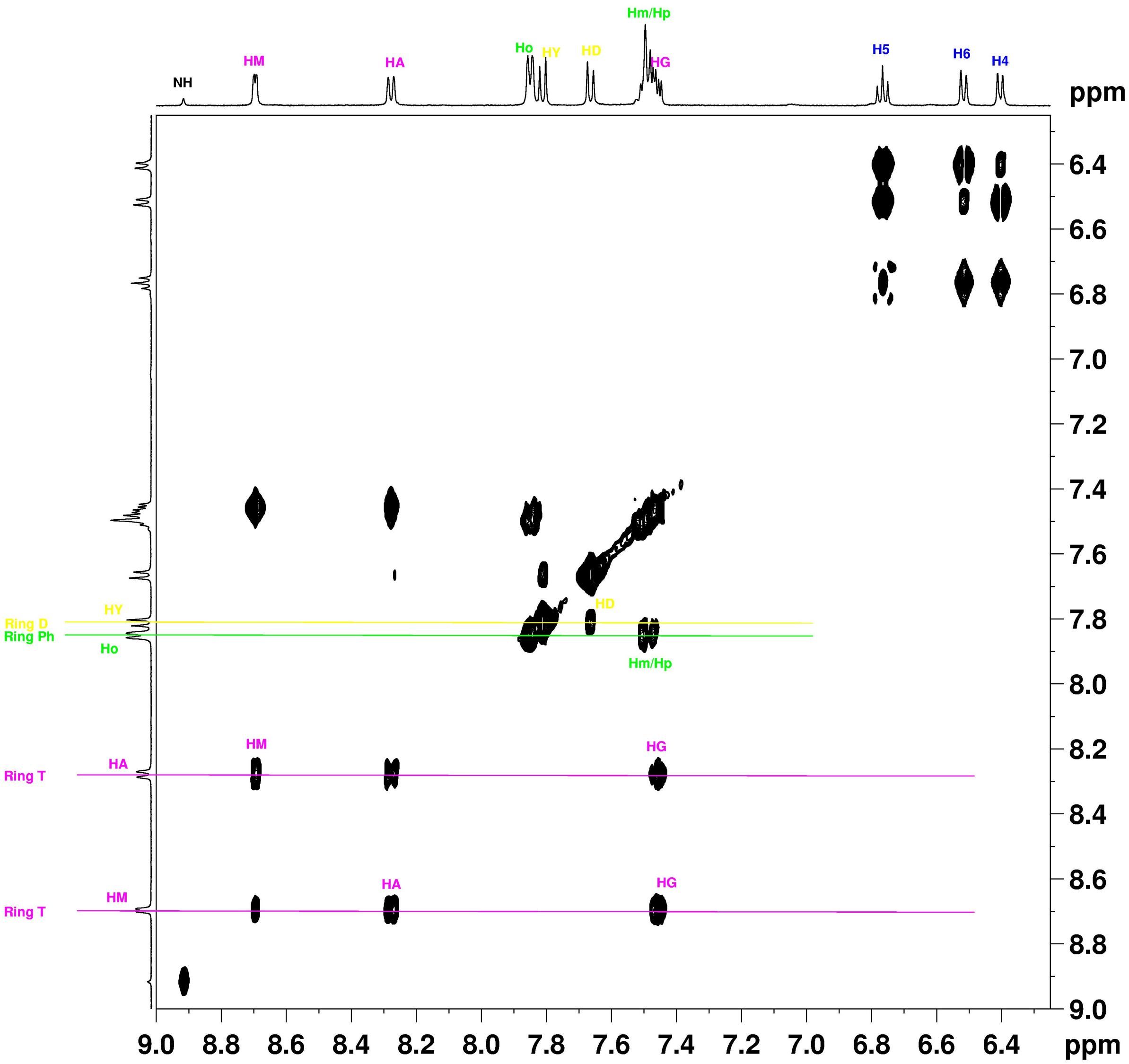

Figure S29. TOCSY ^{1}H–^{1}H NMR spectra of freshly generated **1g-H** recorded in DMSO-d_6 containing a drop of CD_2Cl_2 and D_2O at 500 MHz.

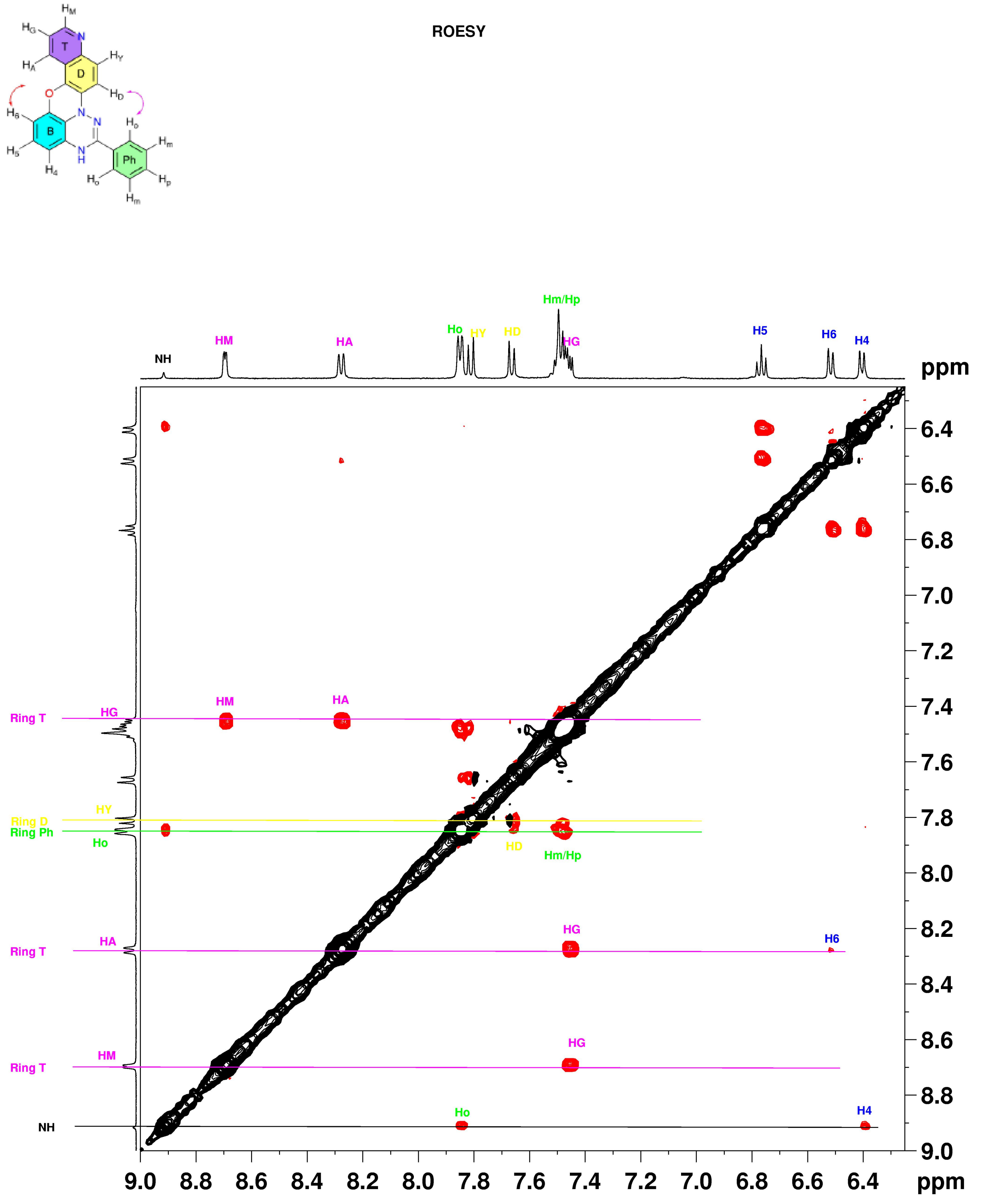

Figure S30. ROESY ^{1}H–^{1}H NMR spectra of freshly generated **1g-H** recorded in DMSO-d_6 containing a drop of CD_2Cl_2 and D_2O at 500 MHz.

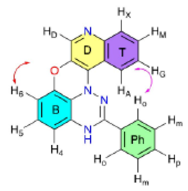

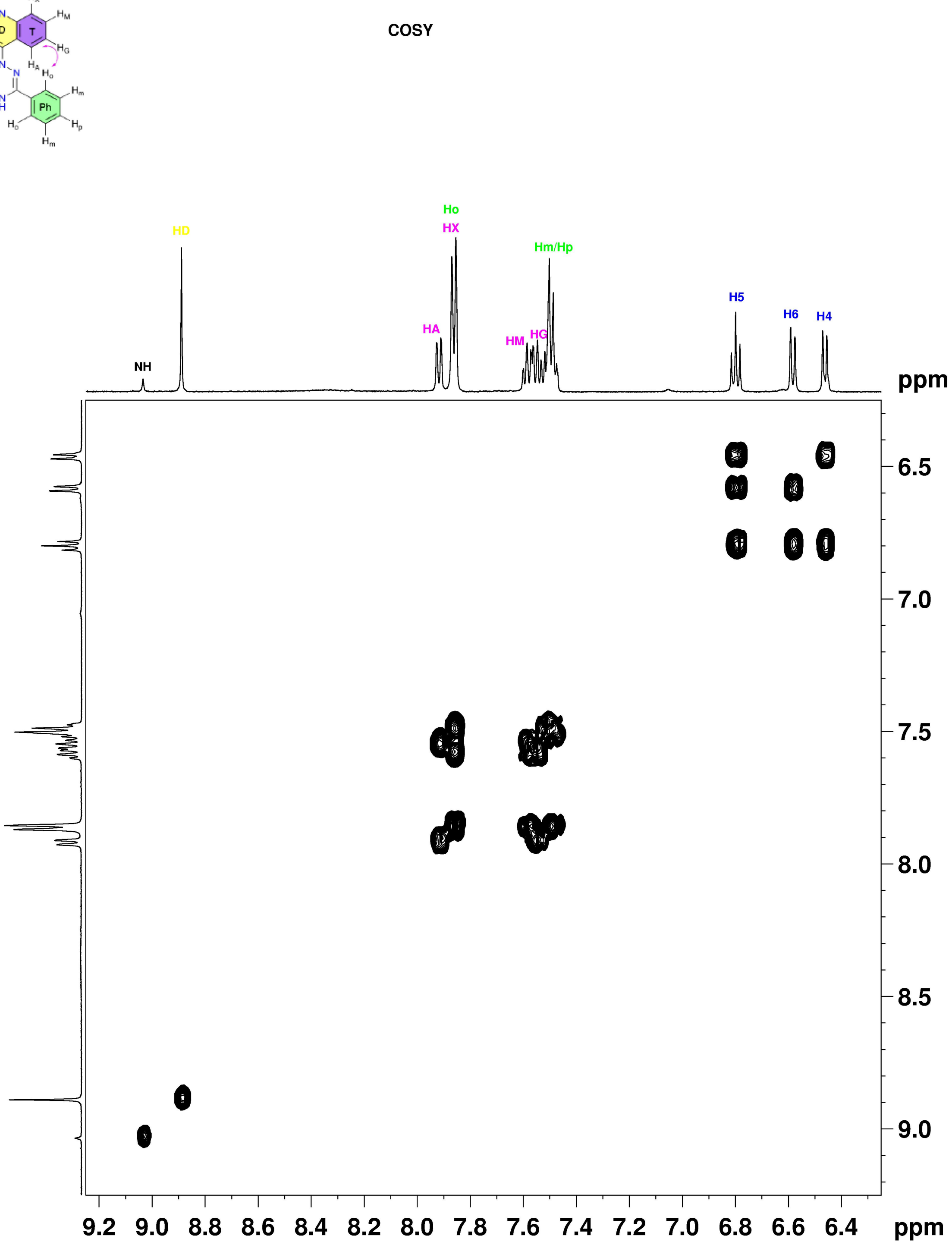

Figure S31. COSY ^{1}H–^{1}H NMR spectra of freshly generated **1h-H** recorded in DMSO-d_6 containing a drop of CD_2Cl_2 and D_2O at 500 MHz.

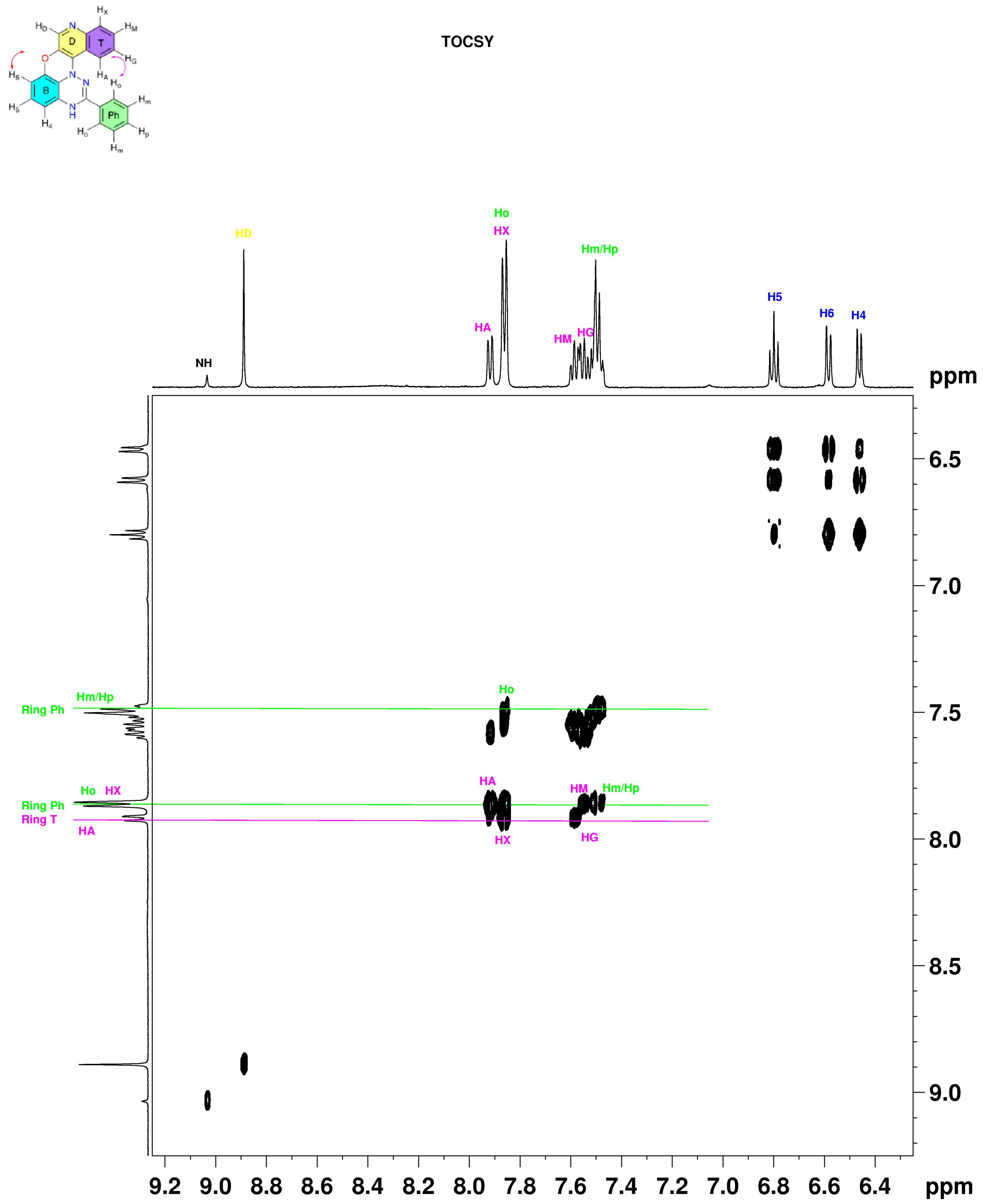

Figure S32. TOCSY ^1H–^1H NMR spectra of freshly generated **1h-H** recorded in DMSO-d_6 containing a drop of CD_2Cl_2 and D_2O at 500 MHz.

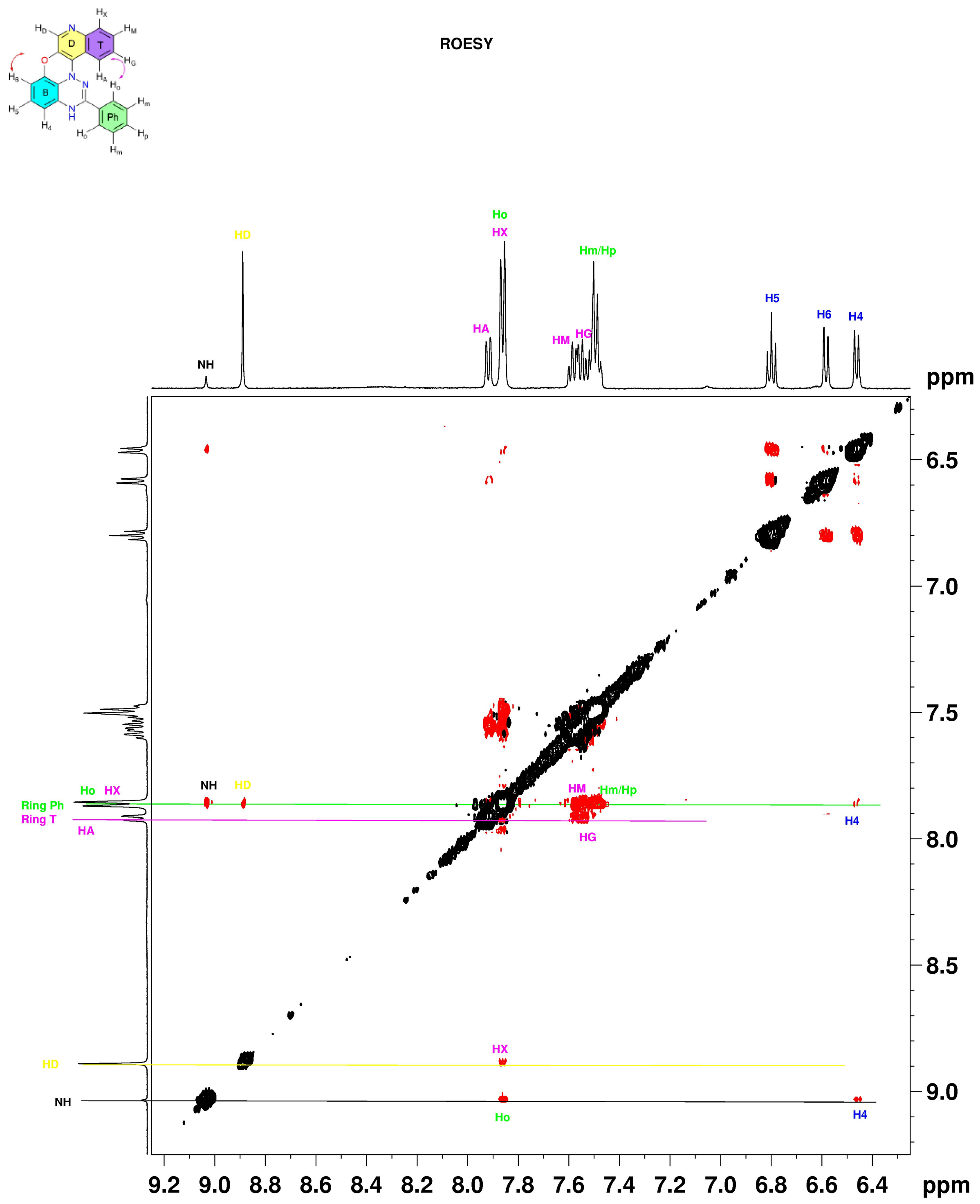

Figure S33. ROESY ^{1}H–^{1}H NMR spectra of freshly generated **1h-H** recorded in DMSO-d_6 containing a drop of CD_2Cl_2 and D_2O at 500 MHz.

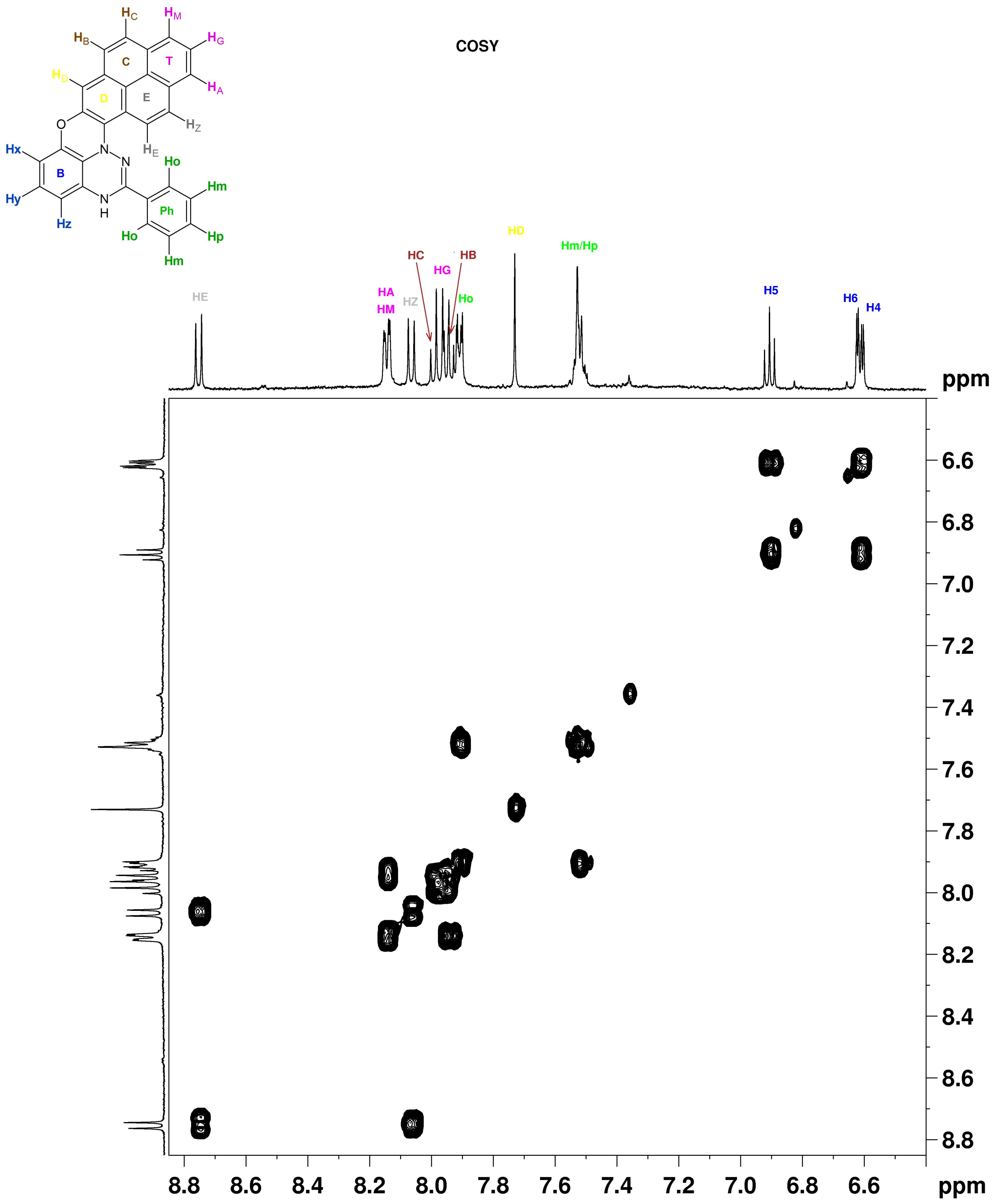

Figure S34. COSY ^1H–^1H NMR spectra of freshly generated **1i-H** recorded in DMSO-d_6 containing a drop of CD_2Cl_2 and D_2O at 500 MHz.

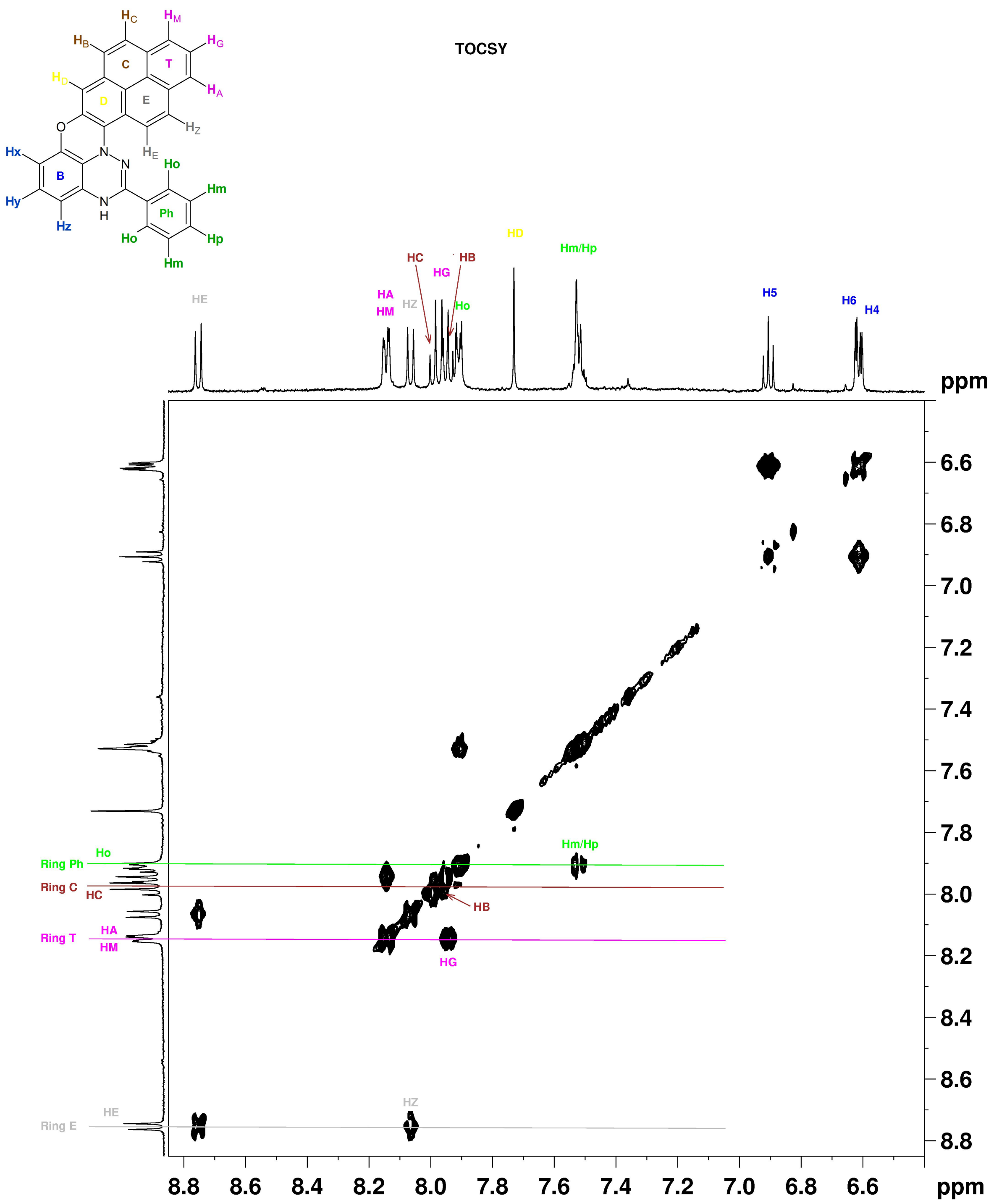

Figure S35. TOCSY ^1H–^1H NMR spectra of freshly generated **1i-H** recorded in DMSO-d_6 containing a drop of CD_2Cl_2 and D_2O at 500 MHz.

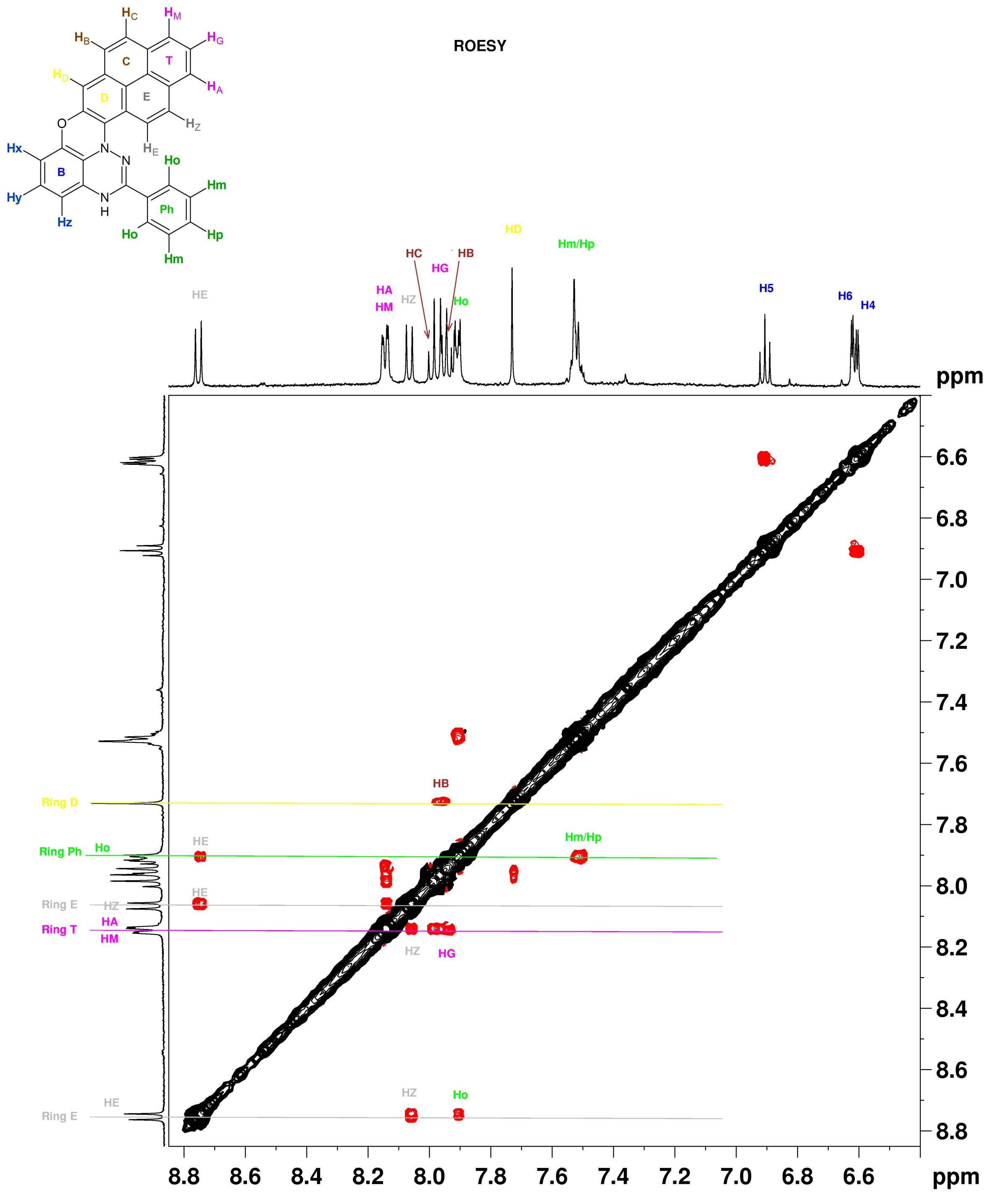

Figure S36. ROESY ^{1}H–^{1}H NMR spectra of freshly generated **1i-H** recorded in DMSO-d_6 containing a drop of CD_2Cl_2 and D_2O at 500 MHz.

4. Computational details

All calculations were carried out using Gaussian 16 suite of programs.[4] Geometry optimizations for radicals **1** were carried out at the UB3LYP/6-31G(2d,p) level of theory in vacuum using tight convergence criteria and appropriate symmetry constrains (either C_s or C_1). Analytical second derivatives were computed using vibrational analysis to confirm each stationary point to be a minimum by yielding zero imaginary frequencies.

a) mechanistic investigation of photocyclization of 2

Mechanistic investigation of photocyclization **2j** in the triplet state (**2jT**) was conducted at the UCAM-B3LYP/6-311G(d,p) level of theory in EtOAc dielectric medium (PCM model[5]) requested with the SCRF(Solvent=EthylEthanoate) keyword and tight convergence limits starting with the GS geometries of **2j**. The transition state structure **2j(T)-TS** to the cyclic product **2j(T)-cycl** was obtained using the QST3 algorithm and the same DFT method in the triplet state. The nature of the resulting structure was verified with the single negative vibrational frequency. Thermodynamic parameters for all three species are collected in Table S1.

Table S1. Thermodynamic parameters for cyclization of triplet state **2j(T)** obtained at the UB3LYP/6-311G(d,p) level of theory in AcOEt dielectric medium.

	E_{SCF} /Ht	ZPEC /Ht	H corr /Ht	G_{298} corr /Ht
**2j(T)**	-970.944560724	0.276427	0.294678	0.227269
**2j(T)-TS**	-970.929753937	0.276395	0.293492	0.230498
**2j(T)-cycl**	-970.959883989	0.278082	0.295249	0.232276

b) isotropic Fermi contact coupling constants (hfcc) for radicals 1

Isotropic Fermi contact coupling constants and spin densities for radicals **1** were calculated using the UCAM-B3LYP/EPR-III // UB3LYP/6-31G(2d,p) method in benzene dielectric medium requested with the SCRF(Solvent=Benzene) keyword (PCM model).[5] The resulting *hfcc* values are shown in Table S2 and spin densities are listed in Table S3.

Table S2. Calculated hyperfine coupling constants (G) of radicals **1** in benzene at the UCAM-B3LYP/EPR-III // UB3LYP/6-31G(2d,p) level of theory.

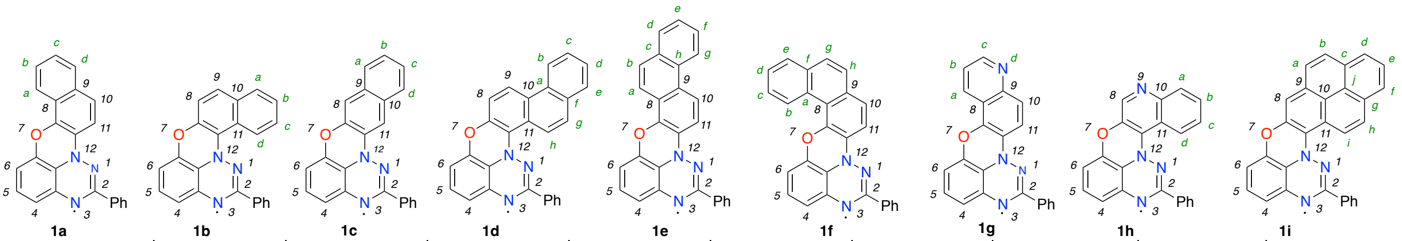

1a 1b 1c 1d 1e 1f 1g 1h 1i

hfcc/G	1a	1b	1c	1d	1e	1f	1g	1h	1i
$a_{N(12)}$	6.36	6.59	5.76	6.47	6.26	6.28	6.22	5.66	6.25
$a_{N(1)}$	3.77	3.64	4.06	3.73	3.83	3.78	3.87	4.03	3.72
$a_{N(3)}$	4.06	4.02	4.19	4.09	4.10	4.06	4.10	4.18	4.01
$a_{H(4)}$	-0.41	-0.52	-0.67	-0.59	-0.43	-0.40	-0.47	-0.66	-0.64
$a_{H(5)}$	-0.89	-0.68	-0.86	-0.67	-0.91	-0.95	-0.82	-0.46	-0.64
$a_{H(6)}$	-0.69	-0.78	-1.02	-0.86	-0.73	-0.69	-0.78	-0.99	-0.92
$a_{H(8)}$	–	1.58	1.18	1.40	–	–	–	1.10	2.10
$a_{H(9)}$	–	-3.27	–	-2.84	–	–	–	1.64 (N)	–
$a_{H(10)}$	1.32	–	–	–	1.22	1.31	1.36	–	–
$a_{H(11)}$	-1.73	–	-3.64	–	-1.96	-2.06	-1.77	–	–
$a_{H(a)}$	-1.37	-1.10	0.96	–	-0.83	–	-1.26	-1.22	1.54
$a_{H(b)}$	0.91	0.90	-1.43	0.43	0.80	0.55	0.87	1.04	-2.16
$a_{H(c)}$	-1.45	-1.22	0.82	-0.43	–	-0.62	-1.37	-1.31	–
$a_{H(d)}$	1.02	1.04	-1.23	0.42	0.48	0.50	-0.62 (N)	1.21	-1.85
$a_{H(e)}$	–	–	–	-0.43	-0.67	-0.58	–	–	0.59
$a_{H(f)}$	–	–	–	–	0.45	–	–	–	-1.85
$a_{H(g)}$	–	–	–	-1.27	-0.61	-1.63			–
$a_{H(h)}$	–	–	–	0.85	–	1.01	–		-2.08
$a_{H(i)}$	–	–	–	–	–	–	–	–	1.58
$a_{H(3\text{-}o)}$ avg	0.47	0.47	0.56	0.50	0.49	0.49	0.49	0.53	0.41
$a_{H(3\text{-}m)}$ avg	-0.28	-0.26	-0.33	-0.28	-0.29	-0.29	-0.29	-0.29	-0.28
$a_{H(3\text{-}p)}$	0.39	0.38	0.47	0.41	0.41	0.41	0.41	0.45	0.51

Table S3. Calculated spin densities of radicals **1** in benzene at the UCAM-B3LYP/EPR-III//UB3LYP/6-31G(2d,p) level of theory.

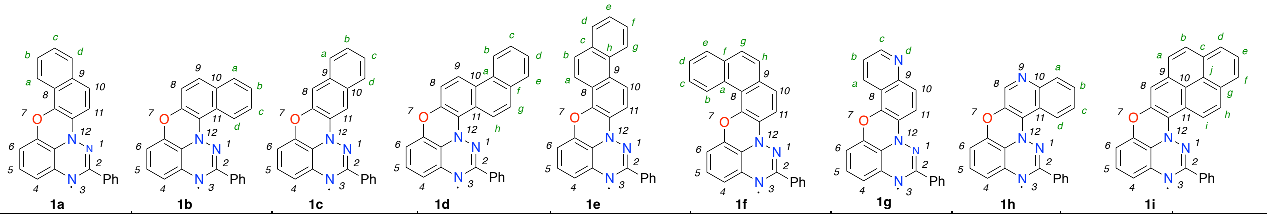

1a 1b 1c 1d 1e 1f 1g 1h 1i

Spin density	**1a**	**1b**	**1c**	**1d**	**1e**	**1f**	**1g**	**1h**	**1i**
$\rho_{N(12)}$	0.244	0.244	0.225	0.242	0.241	0.243	0.240	0.216	0.230
$\rho_{N(1)}$	0.265	0.266	0.291	0.275	0.270	0.265	0.270	0.284	0.272
$\rho_{C(2)}$	-0.050	-0.064	-0.060	-0.067	-0.052	-0.056	-0.055	-0.071	-0.068
$\rho_{N(3)}$	0.273	0.272	0.283	0.278	0.277	0.273	0.276	0.285	0.273
$\rho_{C(3a)}$	0.013	0.006	0.014	0.006	0.013	0.016	0.012	-0.001	0.006
$\rho_{C(4)}$	0.000	0.006	0.011	0.008	0.004	0.001	0.004	0.010	0.012
$\rho_{C(5)}$	0.027	0.022	0.028	0.022	0.029	0.029	0.028	0.014	0.019
$\rho_{C(6)}$	0.019	0.020	0.030	0.025	0.021	0.022	0.021	0.028	0.029
$\rho_{C(6a)}$	0.028	0.019	0.019	0.015	0.027	0.031	0.024	0.007	0.015
$\rho_{C(3a')}$	0.073	0.081	0.068	0.082	0.072	0.070	0.077	0.096	0.080
$\rho_{O(7)}$	0.039	0.036	0.019	0.032	0.035	0.035	0.038	0.036	0.027
$\rho_{C(7a)}$	0.132	0.145	0.056	0.119	0.108	0.107	0.129	0.151	0.103
$\rho_{C(8)}$	-0.040	-0.073	-0.045	-0.063	-0.038	-0.038	-0.039	-0.058	-0.084
$\rho_{C(9)}$	0.059	0.117	0.056	0.100	0.068	0.074	0.058	0.108	0.094
$\rho_{C(10)}$	-0.054	-0.043	-0.032	-0.038	-0.049	-0.055	-0.056	-0.045	-0.040
$\rho_{C(11)}$	0.053	0.041	0.114	0.051	0.056	0.065	0.055	0.044	0.079
$\rho_{C(11a)}$	-0.078	-0.087	-0.067	-0.073	-0.068	-0.075	-0.078	-0.091	-0.095
$\rho_{C(a)}$	0.054	0.044	-0.045	0.023	0.034	0.025	0.049	0.048	-0.067
$\rho_{C(b)}$	-0.037	-0.036	0.056	-0.019	-0.031	-0.021	-0.036	-0.039	0.083
$\rho_{C(c)}$	0.056	0.047	-0.033	0.016	0.027	0.024	0.049	0.050	-0.046
$\rho_{C(d)}$	-0.045	-0.039	0.046	-0.016	-0.021	-0.019	-0.036	-0.043	0.073
$\rho_{C(e)}$	–			0.017	0.026	0.022			-0.044
$\rho_{C(f)}$	–			-0.020	-0.019	-0.023			0.071
$\rho_{C(g)}$	–			0.048	0.024	0.062			-0.044
$\rho_{C(h)}$	–			-0.033	-0.023	-0.045			0.079
$\rho_{C(i)}$	–			–	–				-0.060
$\rho_{C(j)}$	–			–	–				0.029
$\rho_{C(3\text{-Ph})}$	0.004	0.006	0.007	0.006	0.004	0.003	0.004	0.008	0.006
$\rho_{C(3\text{-}o)}$ avg	-0.020	-0.019	-0.024	-0.020	-0.021	-0.020	- 0.021	-0.020	-0.019
$\rho_{C(3\text{-}m)}$ avg	0.012	0.010	0.013	0.011	0.012	0.012	0.012	0.011	0.011
$\rho_{C(3\text{-}p)}$	-0.016	-0.015	-0.019	-0.016	-0.017	-0.016	- 0.016	-0.017	-0.016

c) spin delocalization of radicals 1

Spin delocalization parameter RDV (Radical Delocalization Value)[6] was calculated according to the formula:

$$RDV = \sum_{i=1}^{n} (\rho_i)^2$$

where spin concentration ρ_i on heavy atoms *i* (hydrogen atoms summed up to heavy atoms) is obtained with the UCAM-B3LYP/EPR-III // UB3LYP/6-31G(2d,p) method in benzene dielectric mediums using the PCM model.[5] For the purpose of this work, an inverse is reported: $RDV^{-1}=1/RDV$, since now larger values corresponds to greater delocalization. Results are shown in Table 4 in the main text.

d) electronic excitations

Electronic excitation energies in CH_2Cl_2 dielectric medium were obtained at the UCAM-B3LYP/6-31++G(2d,p) // UB3LYP/6-31G(2d,p) level of theory using the time-dependent TD-DFT method[7] supplied in the Gaussian 16 package. Solvation models in calculations were implemented with the PCM model[5] using the SCRF(solvent=CH_2Cl_2) keyword. Three lowest excitation energies, classified as $\pi\rightarrow\pi^*$ transitions are listed in Table S4. Energies of FMOs involved in the low energy transitions are listed in Table S5.

Table S4. Electronic transition energies and oscillator strength values with the indicated main electronic transition obtained at the TD-CAMB3LYP/6-31++G(2d,p) // UB3LYP/6-31G(2d,p) level of theory in CH_2Cl_2 dielectric medium.

Radical	π→π* β-HOMO→β-LUMO /nm (*f*)	π→π* α-HOMO→α-LUMO /nm (*f*)	π→π* α-HOMO→α-LUMO+1 /nm (*f*)
**1a**	563.7 (0.061)	542.8 (0.013)	496.2 (0.056)
**1b**	569.5 (0.037)	504.0 (0.074)	525.4 (0.017)
**1c**	545.8 (0.068)	510.9 (0.017)	499.5 (0.028) [a]
**1d**	561.5 (0.060)	505.9 (0.052)	520.7 (0.006)<br>423.0 (0.052)
**1e**	553.9 (0.095)	536.4 (0.001)	497.3 (0.046) [b]
**1f**	564.7 (0.085)	537.4 (0.003)	498.7 (0.072) [b]
**1g**	559.9 (0.027)	544.2 (0.037) [c]	494.1 (0.060)
**1h**	584.5 (0.031)	515.3 (0.047) [d]	485.2 (0.035) [e]
**1i**	676.2 (0.003) [f]	548.8 (0.172) [g]	510.6 (0.048)<br>447.1 (0.315) [h]

[a] Equal contribution from α-HOMO→ α-LUMO and β-HOMO→β-LUMO. [b] Complex pattern. Contribution from α-HOMO→ α-LUMO+1 only about 23%. [c] Equal contribution from α-HOMO→ α-LUMO, α-HOMO→ α-LUMO+1, and β-HOMO→β-LUMO. [d] Equal contribution from α-HOMO→α-LUMO and α-HOMO→ α-LUMO+1. [e] 25% contribution from α-HOMO→α-LUMO and 48% of α-HOMO→ α-LUMO+1. [f] Equal contribution from α-HOMO→α-LUMO and β-HOMO→β-LUMO. [g] 36% contribution from β-HOMO-1→β-LUMO and 29% contribution from and β-HOMO→β-LUMO. [h] 38% contribution from α-HOMO→α-LUMO and 25% from β-HOMO-1→β-LUMO.

Table S5. Energies of MOs involved in low energy transitions obtained from the TD-CAMB3LYP/6-31++G(2d,p)//UB3LYP/6-31G(2d,p) method in CH_2Cl_2 dielectric medium.

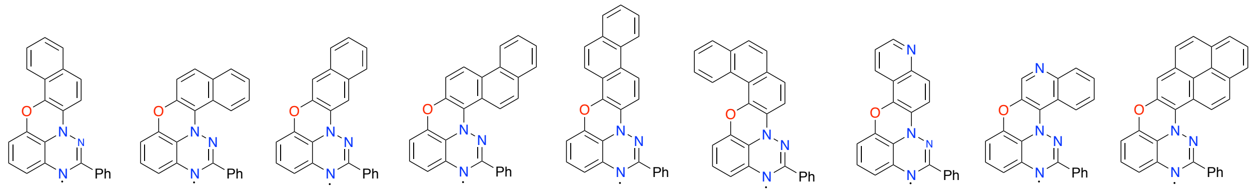

1a 1b 1c 1d 1e 1f 1g 1h 1i

Radical	α-HOMO /eV	α-LUMO /eV	α-LUMO+1 /eV	β-HOMO /eV	β-LUMO /eV	β-LUMO+1 /eV
**1a**	-6.113	-0.653	-0.474	-7.012	-1.817	-0.566
**1b**	-6.108	-0.605	-0.465	-7.044	-1.827	-0.540
**1c**	-6.238	-0.682	-0.249	-7.217	-1.977	-0.614
**1d**	-6.156	-0.630	-0.466	-7.071	-1.879	-0.583
**1e**	-6.128	-0.677	-0.441	-7.051	-1.860	-0610
**1f**	-6.095	-0.639	-0.483	-7.014	-1884	-0.574
**1g**	-6.199	-0.933	-0.595	-7.159	-1885	-0.814
**1h**	-6.403	-0.882	-0.620	-7.252	-2.110	-0.748
**1i**	-6.033	-0.996	-0.528	-6.957	-2.045	-0.799

e) partial output data from TD-DFT calculations for radicals 1

Method: UCAM-B3LYP/6-31++G(2d,p)// UB3LYP/6-31G(2d,p)

Keywords: TD(nstates=20, root=1) SCRF(solvent=CH2CL2) SCF=tight

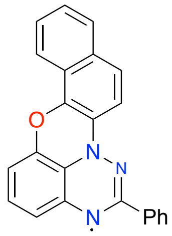

1a

```
Excited State   1:  2.408-A'     2.1997 eV  563.65 nm  f=0.0607
<S**2>=1.200
     89A -> 93A        -0.12719
     90A -> 92A        -0.13076
     91A -> 92A         0.28902
     91A -> 93A        -0.20855
     88B -> 91B        -0.13124
     89B -> 93B         0.17154
     90B -> 91B         0.81328
     90B -> 92B         0.12321
     90B -> 93B        -0.12359
 This state for optimization and/or second-order correction.
 Total Energy, E(TD-HF/TD-KS) =  -1123.74503940
 Copying the excited state density for this state as the 1-particle RhoCI
density.

 Excited State   2:  2.354-A'     2.2842 eV  542.80 nm  f=0.0132
<S**2>=1.136
     90A -> 93A         0.24517
```

```
      91A -> 92A        0.77767
      91A -> 93A        0.13228
      91A -> 94A       -0.18104
      91A ->102A       -0.11958
      89B -> 91B       -0.16117
      90B -> 91B       -0.31250
      90B -> 93B       -0.18754

 Excited State   3:  2.728-A'     2.4987 eV  496.19 nm  f=0.0563
<S**2>=1.611
      89A -> 92A       -0.15763
      89A -> 94A       -0.10017
      90A -> 92A        0.29190
      90A -> 93A       -0.17734
      91A -> 92A        0.17257
      91A -> 93A        0.64126
      91A ->102A       -0.13902
      87B -> 91B       -0.11415
      88B -> 91B       -0.12981
      89B -> 92B        0.15494
      89B -> 93B       -0.13056
      90B -> 91B        0.30398
      90B -> 92B       -0.18895
      90B -> 93B        0.26368

 Excited State   4:  2.779-A'     3.1267 eV  396.53 nm  f=0.0187
<S**2>=1.681
      86A -> 94A        0.10040
      87A -> 92A       -0.13146
      89A -> 93A       -0.24745
      89A -> 94A        0.10133
      90A -> 92A       -0.19753
      90A -> 93A        0.11110
      91A -> 92A       -0.14834
      91A -> 93A        0.54066
      91A -> 94A        0.30009
      91A -> 98A        0.14339
      91A ->101A        0.19174
      88B -> 92B        0.17908
      89B -> 93B        0.24264
      89B -> 94B        0.14648
      90B -> 91B       -0.10155
      90B -> 92B        0.26523
      90B -> 93B       -0.19247

 Excited State   5:  3.240-A'     3.2092 eV  386.34 nm  f=0.0335
<S**2>=2.375
      87A -> 92A       -0.12092
      87A -> 93A       -0.15995
      87A -> 98A        0.14487
      87A ->101A       -0.10417
      88A -> 93A       -0.10482
      88A -> 98A       -0.16786
      88A ->102A       -0.14280
      89A -> 92A       -0.25137
      89A -> 93A       -0.14455
      90A -> 92A       -0.17973
      90A -> 93A       -0.22288
      91A -> 92A        0.19985
      91A -> 93A       -0.19349
      91A ->102A        0.10452
```

```
     86B -> 98B        0.24070
     88B -> 92B        0.17546
     88B -> 93B        0.16066
     88B ->102B       -0.15131
     89B -> 91B        0.20236
     89B -> 92B        0.28695
     89B -> 93B        0.10774
     90B -> 91B       -0.17130
     90B -> 92B        0.24797
     90B -> 93B        0.29135
```

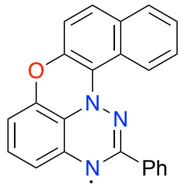

1b

```
 Excited State   1:  2.367-A      2.1772 eV  569.46 nm
f=0.0365  <S**2>=1.151
     89A -> 92A        0.10802
     90A -> 93A       -0.10670
     91A -> 92A       -0.30468
     91A -> 93A       -0.23896
     89B -> 92B       -0.13671
     89B -> 93B       -0.11098
     90B -> 91B        0.83264
 This state for optimization and/or second-order correction.
 Total Energy, E(TD-HF/TD-KS) =  -1123.73599520
 Copying the excited state density for this state as the 1-particle RhoCI
density.

 Excited State   2:  2.484-A      2.3597 eV  525.43 nm  f=0.0170
<S**2>=1.292
     89A -> 92A       -0.10275
     90A -> 92A        0.27420
     91A -> 92A       -0.14831
     91A -> 93A        0.77292
     91A -> 99A        0.11328
     89B -> 91B        0.17620
     90B -> 91B        0.23184
     90B -> 92B       -0.20320
     90B -> 93B       -0.13460

 Excited State   3:  2.410-A      2.4598 eV  504.04 nm  f=0.0735
<S**2>=1.203
     89A -> 93A       -0.13753
     89A -> 94A        0.10824
     90A -> 93A        0.21814
     91A -> 92A        0.75397
     91A -> 93A       -0.10905
     91A ->102A       -0.14238
     89B -> 91B        0.17937
     89B -> 93B        0.11640
     90B -> 91B        0.35499
     90B -> 92B       -0.11178
     90B -> 93B       -0.17431

 Excited State   4:  2.657-A      3.1493 eV  393.69 nm  f=0.0700
<S**2>=1.514
     89A -> 92A       -0.22896
     89A -> 93A       -0.19227
```

```
     89A -> 94A        0.11057
     90A -> 92A        0.12766
     91A -> 92A       -0.38958
     91A -> 93A       -0.35407
     91A -> 94A       -0.25273
     91A -> 98A       -0.13182
     91A -> 99A       -0.23455
     89B -> 91B        0.39911
     89B -> 92B        0.19221
     89B -> 93B        0.21638
     89B -> 95B       -0.12248
     90B -> 92B       -0.21251
     90B -> 93B       -0.17363

 Excited State   5:  3.306-A      3.2215 eV  384.87 nm  f=0.0132
<S**2>=2.482
     87A -> 98A       -0.23872
     87A -> 99A        0.11351
     88A -> 92A       -0.26067
     88A -> 93A        0.20156
     88A ->102A       -0.14163
     89A -> 92A       -0.15334
     90A -> 92A       -0.21624
     90A -> 93A        0.18132
     91A -> 93A        0.13009
     84B ->103B       -0.11072
     85B -> 91B        0.17724
     86B -> 98B        0.12161
     87B -> 98B       -0.22467
     88B -> 91B        0.23441
     88B -> 92B       -0.29861
     88B -> 93B        0.16801
     88B ->103B       -0.15560
     89B -> 92B        0.16559
     90B -> 91B        0.16404
     90B -> 92B        0.33239
     90B -> 93B       -0.15148
```

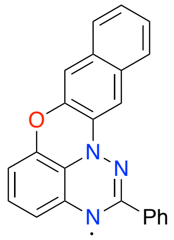

1c

```
 Excited State   1:  2.337-A'     2.2714 eV  545.84 nm  f=0.0684
<S**2>=1.115
     89A -> 93A       -0.10863
     90A -> 92A       -0.12632
     91A -> 92A        0.34366
     89B -> 91B       -0.40999
     90B -> 91B        0.72986
     90B -> 92B        0.13672
 This state for optimization and/or second-order correction.
 Total Energy, E(TD-HF/TD-KS) =  -1123.74336527
 Copying the excited state density for this state as the 1-particle RhoCI
density.

 Excited State   2:  2.469-A'     2.4269 eV  510.88 nm  f=0.0174
<S**2>=1.274
     89A -> 92A       -0.19370
```

```
      89A -> 93A        0.11086
      90A -> 93A       -0.20617
      91A -> 92A        0.64350
      91A -> 93A        0.35210
      91A -> 94A        0.17471
      91A ->101A        0.15966
      88B -> 91B        0.16030
      89B -> 91B        0.31819
      89B -> 92B       -0.13966
      89B -> 93B        0.19194
      90B -> 93B        0.11278
      90B -> 94B       -0.10885

 Excited State   3:  2.751-A'     2.4823 eV  499.48 nm  f=0.0276
<S**2>=1.642
      89A -> 92A       -0.10127
      89A -> 93A        0.26795
      90A -> 92A        0.13687
      90A -> 93A       -0.21209
      90A -> 94A        0.12166
      91A -> 92A       -0.47226
      86B -> 91B        0.12438
      89B -> 91B        0.32258
      89B -> 92B       -0.18361
      89B -> 93B        0.30357
      90B -> 91B        0.50482
      90B -> 94B       -0.11623

 Excited State   4:  2.971-A'     3.1611 eV  392.22 nm  f=0.0491
<S**2>=1.956
      87A -> 98A        0.17010
      88A -> 92A       -0.18781
      88A -> 93A       -0.14357
      88A ->101A        0.13232
      89A -> 92A       -0.18967
      90A -> 92A       -0.26252
      90A -> 93A        0.12133
      91A -> 92A       -0.26729
      91A -> 93A        0.46836
      91A -> 94A        0.25636
      91A ->103A        0.10059
      86B -> 91B        0.10838
      87B -> 98B        0.17272
      88B -> 91B        0.11862
      88B -> 92B        0.21203
      88B -> 93B        0.11754
      88B ->102B       -0.10165
      90B -> 91B       -0.21678
      90B -> 92B        0.31164
```

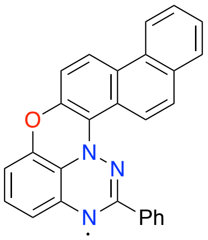

1d

```
 Excited State   1:  2.367-A      2.2081 eV  561.49 nm
f=0.0595  <S**2>=1.150
    102A ->107A        0.11156
```

```
    104A ->105A        0.20610
    104A ->106A        0.15968
    100B ->104B        0.12620
    101B ->105B        0.10839
    102B ->104B        0.17141
    102B ->107B       -0.11576
    103B ->104B        0.84452
 This state for optimization and/or second-order correction.
 Total Energy, E(TD-HF/TD-DFT) =  -1277.30076724
 Copying the excited state density for this state as the 1-particle RhoCI
density.

 Excited State   2:  2.645-A      2.3812 eV  520.69 nm  f=0.0059
<S**2>=1.499
    102A ->105A       -0.25813
    102A ->107A        0.10813
    103A ->107A        0.19375
    104A ->105A       -0.31476
    104A ->106A        0.63029
    104A ->107A       -0.21540
    101B ->104B        0.15795
    102B ->104B        0.15271
    102B ->105B        0.16663
    102B ->107B       -0.17062
    103B ->104B       -0.14329
    103B ->105B        0.12778
    103B ->106B        0.16322

 Excited State   3:  2.554-A      2.4508 eV  505.90 nm  f=0.0521
<S**2>=1.381
    102A ->106A       -0.15311
    102A ->107A        0.23761
    103A ->106A        0.12510
    104A ->105A        0.69456
    104A ->107A        0.12199
    100B ->104B       -0.14792
    102B ->104B        0.19036
    102B ->105B        0.17010
    102B ->107B       -0.18940
    103B ->104B       -0.33703
    103B ->106B        0.14059

 Excited State   4:  2.711-A      2.9309 eV  423.03 nm  f=0.0589
<S**2>=1.587
    102A ->105A        0.15911
    102A ->107A       -0.22190
    103A ->105A        0.12477
    103A ->106A        0.10134
    104A ->105A        0.33055
    104A ->106A        0.50527
    104A ->107A        0.25965
    104A ->111A        0.17833
    102B ->104B       -0.35223
    102B ->105B       -0.22304
    102B ->107B        0.25814

 Excited State   5:  3.155-A      3.2174 eV  385.35 nm  f=0.0181
<S**2>=2.238
     99A ->105A        0.17119
    100A ->112A       -0.20458
    101A ->105A       -0.22454
```

```
     101A ->107A       -0.15267
     102A ->105A       -0.10169
     103A ->105A        0.22465
     104A ->106A       -0.18383
      97B ->104B        0.27558
      98B ->104B        0.17870
      99B ->112B        0.23563
     100B ->104B        0.11323
     100B ->105B       -0.17359
     100B ->117B       -0.12272
     101B ->104B        0.25097
     101B ->105B       -0.24776
     101B ->107B       -0.15642
     103B ->104B        0.15250
     103B ->105B        0.32714
     103B ->107B        0.10160

 Excited State   6:   2.675-A      3.2827 eV  377.69 nm  f=0.0170
<S**2>=1.538
      99A ->107A       -0.10195
     101A ->105A        0.12513
     101A ->106A       -0.12153
     103A ->107A       -0.22674
     104A ->105A        0.22586
     104A ->106A        0.10679
     104A ->107A       -0.42874
     104A ->111A        0.16027
      97B ->104B        0.44336
      98B ->104B        0.24058
      99B ->112B       -0.10221
     100B ->107B        0.10432
     101B ->105B        0.11893
     101B ->106B       -0.13617
     102B ->104B        0.24461
     102B ->106B        0.11746
     102B ->107B        0.14245
     103B ->106B       -0.13512
     103B ->107B       -0.16814
```

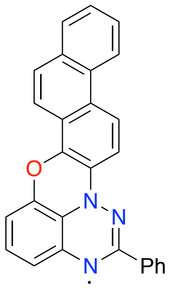

1e

```
 Excited State 1:  2.342-A' 2.2382 eV  553.94 nm f=0.0954
<S**2>=1.121
     103A ->105A        0.13813
     104A ->106A       -0.17004
     100B ->104B       -0.11382
     101B ->106B       -0.11332
     102B ->104B       -0.26513
     103B ->104B        0.84853
     103B ->105B       -0.12456
 This state for optimization and/or second-order correction.
 Total Energy, E(TD-HF/TD-KS) =  -1277.31109373
 Copying the excited state density for this state as the 1-particle RhoCI
density.
```

```
 Excited State   2:  2.376-A'     2.3113 eV  536.43 nm  f=0.0008
<S**2>=1.161
    102A ->105A        0.14514
    103A ->106A       -0.19978
    103A ->107A        0.10869
    104A ->105A        0.76385
    104A ->107A       -0.36894
    104A ->115A        0.10882
    104A ->116A       -0.13463
    101B ->104B       -0.12415
    102B ->107B        0.10266
    103B ->106B        0.17600

 Excited State   3:  2.975-A'     2.4933 eV  497.26 nm  f=0.0464
<S**2>=1.962
     98A ->112A        0.12561
    102A ->105A       -0.10070
    102A ->107A       -0.27662
    103A ->105A       -0.27066
    103A ->106A        0.21347
    103A ->107A       -0.10420
    104A ->105A        0.26804
    104A ->106A        0.46017
    104A ->116A       -0.10456
     98B ->113B        0.12045
    100B ->104B       -0.14528
    101B ->104B        0.10024
    101B ->106B        0.11030
    102B ->105B        0.21268
    102B ->107B       -0.28017
    103B ->104B        0.30242
    103B ->105B        0.15779
    103B ->106B       -0.25129

 Excited State   4:  2.706-A'     2.9936 eV  414.17 nm  f=0.0426
<S**2>=1.581
    102A ->105A        0.14931
    102A ->106A       -0.11843
    102A ->107A        0.17193
    103A ->105A        0.10277
    103A ->107A        0.19286
    104A ->106A        0.64870
    104A ->107A        0.20001
    104A ->111A        0.21141
    104A ->115A       -0.14007
    102B ->104B       -0.24227
    102B ->105B       -0.17370
    102B ->107B        0.24737
    103B ->104B       -0.12765
    103B ->105B       -0.11419
    103B ->107B        0.15208

 Excited State   5:  3.128-A'     3.1988 eV  387.60 nm  f=0.0063
<S**2>=2.196
     99A ->105A        0.10094
     99A ->111A       -0.10564
     99A ->115A       -0.15139
    100A ->105A        0.13049
    100A ->116A        0.14130
    101A ->106A        0.12961
```

```
    101A ->107A       -0.15291
    102A ->105A        0.21534
    103A ->105A       -0.26167
    104A ->105A       -0.29332
    104A ->106A        0.18174
    104A ->107A       -0.30958
    104A ->112A        0.14657
     99B ->111B       -0.18743
    100B ->105B        0.15992
    100B ->118B        0.12618
    101B ->104B       -0.18801
    101B ->105B        0.14414
    101B ->106B        0.10813
    101B ->107B        0.14775
    102B ->104B       -0.14895
    102B ->105B       -0.14605
    102B ->106B       -0.12527
    103B ->104B        0.13391
    103B ->105B        0.32680

 Excited State   6:  2.825-A'     3.2365 eV  383.08 nm  f=0.0435
<S**2>=1.745
     99A ->115A       -0.13297
    100A ->116A        0.10585
    101A ->105A        0.20422
    103A ->106A       -0.15155
    103A ->107A        0.13981
    104A ->105A        0.19897
    104A ->107A        0.53152
    104A ->112A       -0.18645
     99B ->111B       -0.15836
    100B ->104B       -0.10744
    100B ->105B        0.10805
    101B ->105B        0.18909
    102B ->104B        0.36667
    102B ->105B       -0.10593
    102B ->106B        0.15593
    103B ->104B        0.13599
    103B ->106B        0.20155
```

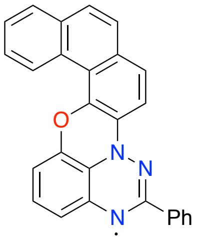

1f

```
 Excited State   1:  2.409-A'     2.1954 eV  564.74 nm
f=0.0845  <S**2>=1.201
    102A ->107A       -0.10273
    104A ->106A       -0.23988
    100B ->104B       -0.12622
    101B ->104B       -0.13872
    101B ->106B        0.14575
    102B ->107B        0.12419
    103B ->104B        0.85468
 This state for optimization and/or second-order correction.
 Total Energy, E(TD-HF/TD-DFT) =  -1277.30655141
 Copying the excited state density for this state as the 1-particle RhoCI
density.
```

```
 Excited State   2:  2.355-A'     2.3072 eV  537.38 nm  f=0.0028
<S**2>=1.137
    103A ->105A        0.17659
    103A ->106A       -0.17832
    103A ->107A       -0.10559
    104A ->105A        0.78286
    104A ->106A        0.20218
    104A ->107A       -0.20127
    104A ->109A        0.14498
    104A ->115A        0.13641
    101B ->104B       -0.12211
    102B ->104B        0.11148
    103B ->104B        0.10810
    103B ->106B       -0.17813

 Excited State   3:  2.791-A'     2.4863 eV  498.66 nm  f=0.0722
<S**2>=1.697
    102A ->107A        0.21042
    103A ->105A       -0.14521
    103A ->106A       -0.25945
    104A ->105A       -0.37060
    104A ->106A        0.49892
    104A ->115A       -0.11935
    100B ->104B       -0.12977
    101B ->106B       -0.11102
    102B ->104B        0.25291
    102B ->107B       -0.17067
    103B ->104B        0.28330
    103B ->106B       -0.30082

 Excited State   4:  2.636-A'     3.0256 eV  409.78 nm  f=0.0316
<S**2>=1.487
    101A ->106A       -0.11130
    102A ->106A        0.14851
    102A ->107A       -0.18852
    103A ->107A       -0.10271
    104A ->106A        0.54701
    104A ->107A        0.36960
    104A ->109A        0.33491
    102B ->104B       -0.31797
    102B ->106B       -0.15167
    102B ->107B        0.21395
    103B ->106B        0.10193

 Excited State   5:  3.289-A'     3.1891 eV  388.77 nm  f=0.0087
<S**2>=2.455
     99A ->105A        0.17062
     99A ->113A        0.19660
    100A ->105A        0.17166
    100A ->113A       -0.14079
    100A ->115A       -0.16399
    101A ->105A        0.19795
    101A ->106A       -0.10910
    101A ->107A       -0.15828
    102A ->105A       -0.11368
    103A ->105A        0.29390
    104A ->105A       -0.17954
    104A ->107A       -0.21851
    104A ->109A        0.10477
     99B ->113B        0.23878
```

```
    100B ->105B        0.25840
    100B ->117B       -0.16282
    101B ->104B       -0.15509
    101B ->105B       -0.20273
    101B ->106B        0.10597
    101B ->107B        0.14562
    102B ->105B        0.11265
    103B ->104B       -0.20441
    103B ->105B        0.37510

 Excited State   6:  2.787-A'     3.2908 eV  376.76 nm  f=0.0731
<S**2>=1.691
     99A ->105A        0.10505
     99A ->113A        0.11100
    101A ->105A        0.15430
    103A ->106A       -0.10634
    103A ->107A       -0.22223
    104A ->105A        0.17817
    104A ->106A       -0.23293
    104A ->107A        0.58654
    104A ->109A       -0.29784
    104A ->121A       -0.13211
     99B ->113B        0.14451
    100B ->105B        0.13687
    101B ->104B       -0.12944
    101B ->105B       -0.16988
    101B ->106B       -0.13644
    102B ->104B       -0.12333
    103B ->106B       -0.19124
    103B ->107B       -0.13384
```

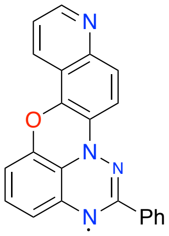

1g

```
 Excited State   1:  2.452-A'     2.2143 eV  559.91 nm  f=0.0274
<S**2>=1.253
     86A -> 92A        0.10198
     89A -> 92A       -0.12769
     90A -> 92A       -0.18160
     91A -> 92A        0.51879
     91A -> 94A       -0.14040
     88B -> 92B       -0.10032
     89B -> 92B        0.10916
     90B -> 91B        0.69819
     90B -> 92B       -0.15855
 This state for optimization and/or second-order correction.
 Total Energy, E(TD-HF/TD-DFT) =  -1139.78577784
 Copying the excited state density for this state as the 1-particle RhoCI
density.

 Excited State   2:  2.337-A'     2.2783 eV  544.19 nm  f=0.0372
<S**2>=1.116
     90A -> 93A       -0.20701
     91A -> 92A       -0.49408
     91A -> 93A       -0.46912
     91A -> 94A        0.18251
     90B -> 91B        0.53943
```

```
     90B -> 92B        0.10866
     90B -> 93B        0.13367

 Excited State   3:  2.550-A'     2.5091 eV  494.14 nm  f=0.0602
<S**2>=1.376
     86A -> 92A       -0.11959
     89A -> 92A        0.17259
     90A -> 92A        0.27845
     91A -> 92A       -0.19095
     91A -> 93A        0.71196
     91A ->102A       -0.15146
     86B -> 91B        0.11725
     89B -> 92B       -0.15046
     90B -> 91B        0.30302
     90B -> 92B        0.24497

 Excited State   4:  2.834-A'     3.1279 eV  396.38 nm  f=0.0221
<S**2>=1.757
     87A -> 92A       -0.22489
     87A -> 94A       -0.10325
     89A -> 92A        0.22004
     90A -> 92A        0.21020
     91A -> 92A        0.45865
     91A -> 93A       -0.31405
     91A -> 94A       -0.20661
     91A -> 98A       -0.22177
     86B -> 91B        0.10194
     87B -> 92B        0.12853
     88B -> 92B        0.22973
     88B -> 94B        0.11150
     89B -> 92B       -0.21902
     89B -> 94B       -0.10150
     90B -> 92B        0.38342

 Excited State   5:  3.325-A'     3.2089 eV  386.37 nm  f=0.0155
<S**2>=2.514
     87A -> 93A        0.15654
     87A ->102A        0.10107
     88A -> 98A       -0.13177
     88A -> 99A       -0.21297
     88A ->102A       -0.11716
     89A -> 93A        0.32317
     89A ->102A        0.14437
     90A -> 93A       -0.32180
     91A -> 92A        0.18467
     91A -> 94A        0.10106
     91A ->102A        0.10737
     83B ->103B       -0.12312
     87B -> 98B        0.22019
     87B ->101B       -0.10484
     88B -> 93B        0.11307
     88B -> 98B       -0.11144
     89B -> 91B        0.18234
     89B -> 92B       -0.11407
     89B -> 93B        0.34675
     89B ->103B       -0.17606
     90B -> 91B       -0.17255
     90B -> 93B        0.38321
```

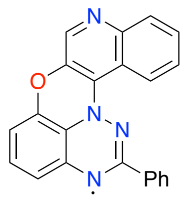

1h

```
 Excited State   1:   2.289-A      2.1210 eV  584.54 nm
f=0.0312  <S**2>=1.060
      89A -> 92A       -0.10690
      91A -> 92A       -0.30323
      89B -> 92B       -0.13783
      90B -> 91B        0.88101
 This state for optimization and/or second-order correction.
 Total Energy, E(TD-HF/TD-DFT) =  -1139.77941214
 Copying the excited state density for this state as the 1-particle RhoCI
density.

 Excited State   2:   2.668-A      2.4063 eV  515.25 nm  f=0.0474
<S**2>=1.530
      89A -> 92A       -0.13507
      90A -> 92A        0.28455
      91A -> 92A       -0.49565
      91A -> 93A        0.52897
      89B -> 91B        0.23757
      89B -> 92B       -0.11473
      90B -> 91B       -0.24975
      90B -> 92B       -0.28586
      90B -> 93B        0.10127

 Excited State   3:   2.204-A      2.5555 eV  485.17 nm  f=0.0345
<S**2>=0.965
      89A -> 93A       -0.11118
      90A -> 93A        0.18366
      91A -> 92A        0.51233
      91A -> 93A        0.69480
      91A ->101A       -0.15181
      88B -> 91B       -0.15569
      90B -> 91B        0.18669

 Excited State   4:   2.748-A      3.0715 eV  403.66 nm  f=0.0903
<S**2>=1.637
      89A -> 92A       -0.32009
      89A -> 94A       -0.10336
      90A -> 92A        0.11949
      91A -> 92A        0.51827
      91A -> 93A       -0.12754
      91A -> 94A       -0.13255
      91A -> 98A       -0.12789
      91A -> 99A       -0.17375
      86B -> 94B        0.10942
      88B -> 91B        0.14860
      88B -> 94B       -0.10120
      89B -> 91B        0.41583
      89B -> 92B       -0.27568
      89B -> 93B        0.14212
      89B -> 94B       -0.12194
      89B -> 99B       -0.10648
      90B -> 92B       -0.23814
      90B -> 93B        0.10497
```

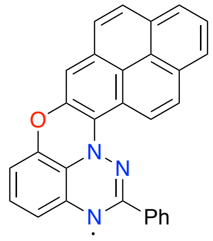

1i

```
            Excited State   1:   2.935-A      1.8337 eV  676.16 nm
f=0.0025  <S**2>=1.903
    109A ->111A       -0.33683
    109A ->113A        0.10114
    110A ->111A        0.53404
    110A ->112A        0.10690
    108B ->110B       -0.12025
    108B ->111B        0.19169
    108B ->113B       -0.12169
    109B ->110B        0.59049
    109B ->111B       -0.26896
    109B ->112B       -0.11628
    109A <-111A       -0.11112
    110A <-111A        0.10064
 This state for optimization and/or second-order correction.
 Total Energy, E(TD-HF/TD-DFT) =  -1353.51101699
 Copying the excited state density for this state as the 1-particle RhoCI
density.

 Excited State   2:   2.390-A      2.2592 eV  548.80 nm  f=0.1721
<S**2>=1.177
    109A ->111A        0.15615
    110A ->111A       -0.18976
    110A ->112A       -0.22232
    106B ->110B       -0.13394
    107B ->111B       -0.10435
    108B ->110B        0.60198
    108B ->111B        0.12222
    109B ->110B        0.53385
    109B ->111B        0.26600

 Excited State   3:   2.132-A      2.4283 eV  510.59 nm  f=0.0476
<S**2>=0.887
    108A ->112A        0.15616
    109A ->112A        0.25905
    110A ->111A       -0.32213
    110A ->112A        0.78570
    110A ->122A       -0.16854
    108B ->110B        0.12641
    109B ->110B        0.17159

 Excited State   4:   2.370-A      2.7731 eV  447.10 nm  f=0.3147
<S**2>=1.154
    108A ->111A       -0.14145
    109A ->111A        0.20606
    110A ->111A        0.61645
    110A ->112A        0.20001
    110A ->113A        0.21945
    110A ->118A        0.15015
    108B ->110B        0.50360
    108B ->111B       -0.14925
    109B ->110B       -0.27372
    109B ->111B        0.10573
```

```
 Excited State   5:  2.684-A      3.1410 eV  394.73 nm  f=0.0191
<S**2>=1.551
    107A ->113A        0.13782
    108A ->111A       -0.18723
    108A ->113A        0.13773
    109A ->111A        0.41145
    109A ->112A        0.13297
    110A ->111A        0.20213
    110A ->113A       -0.37468
    110A ->118A        0.17182
    102B ->110B       -0.15620
    108B ->110B       -0.31914
    108B ->111B       -0.19157
    108B ->113B        0.13448
    109B ->110B        0.34991
    109B ->111B        0.14829
    109B ->112B        0.18124

 Excited State   6:  3.077-A      3.1897 eV  388.70 nm  f=0.0681
<S**2>=2.116
    105A ->112A        0.14918
    106A ->119A        0.17479
    107A ->112A       -0.24480
    108A ->111A       -0.11069
    108A ->112A        0.16013
    108A ->113A       -0.11470
    110A ->111A       -0.17284
    110A ->112A       -0.16446
    110A ->113A        0.26165
    103B ->110B       -0.20907
    105B ->118B       -0.21289
    106B ->111B       -0.12023
    106B ->112B        0.13169
    106B ->123B        0.10442
    107B ->110B        0.31170
    107B ->111B        0.13191
    107B ->112B       -0.25240
    107B ->123B       -0.10691
    108B ->110B        0.10942
    108B ->111B       -0.25057
    108B ->113B       -0.12716
    109B ->110B        0.16478
    109B ->111B       -0.21841
    109B ->112B        0.12859
```

5. Archive for DFT geometry optimization results.

1a


```
1\1\GINC-LOCALHOST\FOpt\UB3LYP\6-31G(2d,p)\C23H14N3O1(2)\PIOTR\07-Nov-
 2019\0\\#P UB3LYP/6-31G(2d,p) FOpt=tight freq(noraman) SCF=Direct #P G
 eom=(NoDistance,NoAngle) fcheck\\benzotrazinyl 1,2-naphthyl, Cs\\0,2\N
 ,0.1295261464,-0.1732301943,0.\N,1.4632907762,-2.5788516909,0.\C,0.134
 085577,-2.4565447692,0.\N,-0.5842294895,-1.3222851346,0.\C,3.611414204
 6,1.0238959089,0.\C,2.2267923101,1.0353358405,0.\C,1.5188888839,-0.167
 6891648,0.\C,2.1799797238,-1.413505952,0.\C,3.5868756977,-1.4091334221
 ,0.\C,4.2797647954,-0.2084114497,0.\C,-0.5464354784,1.0534738762,0.\C,
 0.1948046563,2.2288606702,0.\C,-0.4401554503,3.4957199894,0.\C,-1.8697
 594646,3.5419573608,0.\C,-1.9617108536,1.1129807333,0.\C,-0.6658490923
 ,-3.70963406,0.\C,-2.0670748452,-3.6831402511,0.\C,-2.7942664337,-4.86
```

85923282,0.\C,-2.1349639176,-6.0973689311,0.\C,-0.7412964779,-6.131996
6521,0.\C,-0.010982711,-4.9482164096,0.\O,1.5693939989,2.2411359494,0.
\H,4.0995651368,-2.3634076606,0.\H,-2.5067805933,0.1798004786,0.\H,-2.
5768041987,-2.7281289763,0.\H,-3.8791112524,-4.8332127721,0.\H,-0.2205
81816,-7.0843256377,0.\H,1.0716340804,-4.9600597695,0.\H,5.3645117358,
-0.215126262,0.\H,4.1484075398,1.9645945355,0.\H,-2.7043205946,-7.0214
907809,0.\C,0.2952736984,4.7091611349,0.\C,-0.3604786801,5.9174356511,
0.\C,-2.5070242523,4.806106268,0.\C,-1.772767142,5.969253622,0.\C,-2.6
000115892,2.3241362828,0.\H,0.2101463062,6.8403615799,0.\H,-2.27576543
07,6.9305700267,0.\H,1.3771028492,4.6660915991,0.\H,-3.5924205711,4.83
9254092,0.\H,-3.6844792823,2.3635924493,0.\\Version=ES64L-G09RevD.01\S
tate=2-A"\HF=-1124.3826466\S2=0.767057\S2-1=0.\S2A=0.750237\RMSD=5.021
e-09\RMSF=1.247e-06\Dipole=-0.1018338,1.0062655,0.\Quadrupole=6.867049
2,4.5520323,-11.4190814,0.5985839,0.,0.\PG=CS [SG(C23H14N3O1)]\\@

1b

1\1\GINC-LOCALHOST\FOpt\UB3LYP\6-31G(2d,p)\C23H14N3O1(2)\PIOTR\21-Aug-
2018\0\\#P UB3LYP/6-31G(2d,p) FOpt=tight freq(noraman) SCF=Direct #P G
eom=(NoDistance,NoAngle) fcheck\\napthto_21 C(8)-O-Ph(N1) benzotraziny
l (phenazinoBT), Cs\\0,2\N,0.433237898,-0.511107587,-0.0280240064\N,-1
.9309227726,-1.9151045011,0.33565156\C,-1.8590117932,-0.6108921412,0.0
735071013\N,-0.7557406232,0.129593157,-0.1132922097\C,1.7156307366,-3.
9709209674,0.1444589881\C,1.6778282977,-2.5951208548,-0.0081076793\C,0
.4735921759,-1.9028970562,0.0942260389\C,-0.7408629441,-2.5884412505,0
.3128717052\C,-0.6903506818,-3.9854687033,0.4739964192\C,0.5192973005,
-4.6561107435,0.3925669323\C,1.6455186942,0.2052790046,-0.1051736248\C
,2.8107455257,-0.5502806854,-0.2678872567\C,4.0703914959,0.0418050701,
-0.4402669131\C,4.1926582691,1.4042827687,-0.4350596932\C,3.0622776469
,2.2268369482,-0.2080337522\C,-3.1380231952,0.1404898954,-0.0250963153
\C,-3.1792153713,1.4508570273,-0.5207756516\C,-4.3884989988,2.13306844
54,-0.6065819354\C,-5.5717065891,1.5196269141,-0.1967207923\C,-5.53859
98917,0.2150616443,0.2940875564\C,-4.3320996264,-0.4722735801,0.376034
8672\O,2.8368971971,-1.9239819081,-0.2855790264\H,4.9189291624,-0.6168
525216,-0.5822646088\H,-2.261041669,1.9215745844,-0.8499149134\H,-4.40
75623422,3.1453030464,-0.9978419596\H,-6.455845589,-0.2696158681,0.613
1481454\H,-4.2921078802,-1.4879664378,0.7492734939\H,0.5460505743,-5.7
337905835,0.513355558\H,2.6652236898,-4.4854078061,0.0625483856\H,5.16
1763643,1.8699597852,-0.579816613\H,-6.5140982081,2.0540831992,-0.2630
645633\H,-1.6231716553,-4.508969042,0.6450501951\C,1.7619862289,1.6431
160565,-0.0146916845\C,0.695138849,2.5309598046,0.2952448636\C,3.22053
65977,3.6334643181,-0.1409189595\C,2.1608834767,4.4591063154,0.1403648
089\C,0.8939431787,3.8917392831,0.3748268723\H,2.2984474413,5.53395388
5,0.1938263925\H,0.0541814877,4.5326275179,0.6235444499\H,4.2128425464
,4.0428819803,-0.3049014463\H,-0.2846077723,2.1238421064,0.4767116208\
\Version=ES64L-G09RevD.01\State=2-A\HF=-1124.3732452\S2=0.769022\S2-1=
0.\S2A=0.750297\RMSD=9.945e-09\RMSF=1.418e-06\Dipole=0.8656581,0.21110
23,-0.0034517\Quadrupole=4.3879227,6.3523177,-10.7402405,-1.5509061,-1
.5171127,-1.3764984\PG=C01 [X(C23H14N3O1)]\\@

1c

1\1\GINC-LOCALHOST\FOpt\UB3LYP\6-31G(2d,p)\C23H14N3O1(2)\PIOTR\23-Jun-
2016\0\\#P UB3LYP/6-31G(2d,p) FOpt=tight freq(noraman) SCF=Direct #P G
eom=(NoDistance,NoAngle) fcheck\\C(8)-O-Ph(N1) benzotrazinyl-Naphthale
ne-2,3, Cs\\0,2\N,0.515497464,-0.1066191248,0.\N,1.1670860049,-2.77826
3284,0.\C,-0.0827216634,-2.3115435001,0.\N,-0.4750328092,-1.0271067714
,0.\C,4.1897459337,0.1240632574,0.\C,2.8581243987,0.5062483554,0.\C,1.
8552154429,-0.4659642903,0.\C,2.1654769098,-1.8434492723,0.\C,3.522003
8636,-2.2142406823,0.\C,4.5088624326,-1.2406357103,0.\C,0.1715500381,1
.2516307802,0.\C,1.2282178949,2.2121946661,0.\C,0.9504100687,3.5516994
505,0.\C,-1.1388622685,1.6813575818,0.\C,-1.1848475438,-3.3088951116,0
.\C,-2.5291987682,-2.9129650478,0.\C,-3.5435927494,-3.8643284597,0.\C,

-3.231860337,-5.2235242923,0.\C,-1.8968713166,-5.6251370533,0.\C,-0.87
98709846,-4.6763721598,0.\O,2.5518208442,1.8413369412,0.\H,3.761534385
4,-3.2706353493,0.\H,-1.9275069732,0.9411786052,0.\H,-2.7684426095,-1.
8572275051,0.\H,-4.5805919382,-3.5439062919,0.\H,-1.6463542607,-6.6811
810233,0.\H,0.1610580652,-4.9741049972,0.\H,5.5530837606,-1.5341614809
,0.\H,4.9565672335,0.8891833866,0.\H,-4.0249562769,-5.9645693438,0.\C,
-1.4520681679,3.0611668816,0.\C,-0.388887876,4.0191305361,0.\C,-0.7080
431648,5.3983941385,0.\C,-2.7923046123,3.5253276572,0.\C,-2.0194165167
,5.8166105618,0.\C,-3.0705525136,4.872113358,0.\H,-2.250341835,6.87684
23762,0.\H,-4.1001578911,5.2143934595,0.\H,1.7774097774,4.2532279687,0
.\H,0.1010879528,6.1226231964,0.\H,-3.5963179144,2.795558613,0.\\Versi
on=ES64L-G09RevD.01\State=2-A"\HF=-1124.3839338\S2=0.767132\S2-1=0.\S2
A=0.750233\RMSD=9.849e-09\RMSF=2.105e-06\Dipole=0.0998354,0.7142974,0.
\Quadrupole=7.8004795,3.4174494,-11.2179289,0.0185252,0.,0.\PG=CS [SG(
C23H14N3O1)]\\@

1d

1\1\GINC-GAUSIANDELL\FOpt\UB3LYP\6-31G(2d,p)\C27H16N3O1(2)\PKASZYNSKI\
03-Sep-2021\0\\#p opt=tight freq=noraman ub3lyp/6-31g(2d,p) scf=direct
#p fcheck geom=(nodistance,noangle)\\8-2Phen-3PhR1_B3LYP631G2dp\\0,2\
N,1.5393429005,0.6565869544,0.1334428574\N,3.7138690819,2.3700032724,0
.0095778642\C,2.4675388756,2.7511536769,0.2877404371\N,1.3733623127,1.
9787830864,0.3695173272\C,4.303113159,-1.7745428116,-0.1652049255\C,3.
0301438308,-1.2538372034,-0.0039285623\C,2.8240681644,0.1236584257,0.0
062186779\C,3.9099406827,1.0205470339,-0.0935149206\C,5.1981466426,0.4
810907601,-0.2656266767\C,5.3820947068,-0.8917298038,-0.3035532786\C,-
0.941287003,0.2066556389,-0.0446379002\C,0.4278699252,-0.2141310321,0.
0844609127\C,0.7080906607,-1.5851714475,0.1553630382\C,-1.9780837615,-
0.7767000466,0.0602881171\C,-0.3109598628,-2.5333248846,0.2151023351\C
,-1.6263150375,-2.1346124635,0.1978800063\H,6.0291676587,1.171204991,-
0.3467653252\H,6.3799638167,-1.297391243,-0.4313766723\H,4.4369310933,
-2.8493397047,-0.1728352162\H,-2.39428739,-2.8939722853,0.2534814706\H
,-0.0291893868,-3.5772463441,0.2838169941\C,-1.3254675855,1.559072454,
-0.3394090405\C,-2.6307417996,1.9176945644,-0.4658998871\C,-3.37564814
41,-0.3719109338,-0.0105423792\C,-3.6949876046,0.9866407157,-0.2797922
214\H,-0.5539594456,2.2974003467,-0.4744588766\H,-2.8815156861,2.94575
81242,-0.7108454971\C,-5.0453512597,1.3919228554,-0.3661467924\C,-4.44
95782727,-1.2727284,0.1771531219\C,-5.7627199366,-0.8552028575,0.09447
25003\H,-6.5632924464,-1.5712996366,0.2489731362\H,-4.2551686241,-2.31
28246781,0.407477942\C,-6.0688428618,0.4882865471,-0.184083585\H,-5.26
06932017,2.4353402252,-0.5768067911\H,-7.1032715242,0.8088309215,-0.25
02445317\O,1.977511191,-2.1087759215,0.1755838533\C,2.2299892144,4.197
2611957,0.5375797867\C,0.9911615963,4.6651831104,0.9973633307\C,3.2644
423226,5.1158501828,0.3174311603\C,0.7928936724,6.022907024,1.22586659
07\H,0.1933564324,3.9567771531,1.1833023792\C,3.0607118272,6.473235168
3,0.5426919909\H,4.2202921459,4.7448348432,-0.0305968587\C,1.825116268
2,6.9321115568,0.9972060646\H,-0.169624333,6.3717032693,1.5863568606\H
,3.8694431094,7.1747436866,0.3641105071\H,1.6680291858,7.9912577034,1.
1751547159\\Version=ES64L-G16RevB.01\State=2-A\HF=-1278.0224028\S2=0.7
67454\S2-1=0.\S2A=0.750252\RMSD=5.665e-09\RMSF=2.324e-07\Dipole=-0.523
9101,-0.7035683,-0.0823392\Quadrupole=6.0397888,6.307755,-12.3475438,-
2.8646099,-1.5124317,-0.2166381\PG=C01 [X(C27H16N3O1)]\\@

1e

1\1\GINC-LOCALHOST\FOpt\UB3LYP\6-31G(2d,p)\C27H16N3O1(2)\PIOTR\16-Oct-
2021\0\\#P UB3LYP/6-31G(2d,p) FOpt=tight freq(noraman) SCF=Direct #P G
eom=(NoDistance,NoAngle) fcheck\\flat_BT-1Oxo-Phenanthrene-2\\0,2\N,1.
1166286654,0.3497311198,0.\C,3.3430605133,-0.1550321472,0.\N,2.0770503
202,-0.6026595543,0.\C,0.72432214,4.0093433131,0.\C,0.404567805,2.6619
204053,0.\C,1.4202172914,1.7045177808,0.\C,2.7818232816,2.0722485061,0
.\C,3.0909475863,3.4446473088,0.\C,2.0743020615,4.3872380675,0.\C,-2.5

86298809,0.6116979005,0.\C,-1.215574521,0.9470812039,0.\C,-0.229453462
9,-0.0372677648,0.\C,-2.9663623342,-0.7618502067,0.\C,-0.6110943933,-1
.3913563803,0.\C,-1.9427104072,-1.7364128955,0.\O,-0.9167702984,2.2898
554358,0.\H,4.1353784489,3.7321140265,0.\H,2.3217206403,5.4433801749,0
.\H,-0.0733179021,4.7422368492,0.\H,-2.1958377731,-2.7887091778,0.\H,0
.1673443529,-2.1410009515,0.\C,-3.5888396802,1.6363378178,0.\C,-4.9070
389802,1.3126742019,0.\C,-4.3793072186,-1.0995158977,0.\C,-5.344424814
5,-0.0511208476,0.\H,-3.271924866,2.6711124847,0.\H,-5.6614300104,2.09
39235945,0.\C,-6.7202978357,-0.366928814,0.\C,-4.8519345169,-2.4315669
387,0.\C,-6.2028033068,-2.7157726807,0.\H,-6.5340241162,-3.7491306554,
0.\H,-4.1486027064,-3.2552918795,0.\C,-7.1493358641,-1.6769390804,0.\H
,-7.4389747185,0.4473468709,0.\H,-8.2096997159,-1.9066088386,0.\N,3.75
80421507,1.1136417967,0.\C,4.3867492138,-1.2136019127,0.\C,5.739957997
1,-0.8505458762,0.\C,4.0488699789,-2.5737414265,0.\C,6.7313578695,-1.8
261119022,0.\H,5.9922947634,0.2022854859,0.\C,5.0426404228,-3.54661163
1,0.\H,3.0042768574,-2.857837494,0.\C,6.3872456414,-3.1771278485,0.\H,
7.7757641157,-1.5306688326,0.\H,4.7668456286,-4.5963822206,0.\H,7.1616
182557,-3.9377477596,0.\\Version=ES64L-G09RevD.01\State=2-A"\HF=-1278.
0333091\S2=0.766013\S2-1=0.\S2A=0.750207\RMSD=4.982e-09\RMSF=1.812e-06
\Dipole=-1.0222218,0.0810862,0.\Quadrupole=3.8081399,8.9836009,-12.791
7408,0.3833365,0.,0.\PG=CS [SG(C27H16N3O1)]\\@

1f

1\1\GINC-LOCALHOST\FOpt\UB3LYP\6-31G(2d,p)\C27H16N3O1(2)\PIOTR\20-Mar-
2025\0\\#P UB3LYP/6-31G(2d,p) FOpt=tight SCF=Direct #P Geom=(NoDistanc
e,NoAngle) fcheck\\[5]helicene, Cs isomerised via Smiles rearr\\0,2\O,
1.4947381681,1.3024890242,0.\N,-2.1966892263,-0.5772881199,0.\C,-4.583
8929661,-0.4960513298,0.\N,-0.9993701329,0.0545345911,0.\C,-5.77477467
08,0.2420929909,0.\N,-3.3114815944,1.5514293022,0.\C,-7.0056643824,-0.
4056373962,0.\C,-7.0663347842,-1.7984023227,0.\C,-5.8856038878,-2.5403
177599,0.\C,-4.6532685978,-1.8958142577,0.\C,-3.27920041,0.2168038099,
0.\C,-0.9041337423,1.4361575446,0.\C,-2.0994179401,2.1845249743,0.\C,0
.1740019026,-0.7144210588,0.\C,1.421546987,-0.0722233178,0.\C,0.345045
2985,2.0505741271,0.\C,0.0958183931,-2.1163928381,0.\C,1.2531336425,-2
.8494929439,0.\C,2.5204236697,-2.2304588383,0.\C,2.6408814033,-0.80290
38301,0.\C,-1.9921799926,3.5870873908,0.\C,0.436967535,3.4327632088,0.
\C,-0.7434004306,4.1895208402,0.\C,3.6858415473,-3.0560647948,0.\C,3.9
870211505,-0.2172330877,0.\C,5.1139633017,-1.1003811927,0.\C,4.9288726
31,-2.5180918926,0.\C,4.2722513497,1.1708331328,0.\C,5.5686864589,1.65
12223261,0.\C,6.6621025304,0.7749831459,0.\C,6.4267929373,-0.581887955
7,0.\H,-5.7127587144,1.3229544481,0.\H,-7.9202107046,0.1788628432,0.\H
,-8.0272694562,-2.3031369122,0.\H,-5.9245210186,-3.6250062721,0.\H,-3.
7355229588,-2.4698670394,0.\H,-0.8807554941,-2.577534316,0.\H,1.206665
6692,-3.9334276235,0.\H,-2.90593324,4.1689018816,0.\H,1.41243285,3.903
6165849,0.\H,-0.6701271376,5.2717512161,0.\H,3.5451913482,-4.132443165
2,0.\H,5.8096333385,-3.1529160469,0.\H,7.6767291831,1.1593501321,0.\H,
7.2548089149,-1.2845692909,0.\H,5.7340043641,2.7239091639,0.\H,3.46553
6908,1.8811409241,0.\\Version=ES64L-G16RevC.01\State=2-A"\HF=-1278.027
7577\S2=0.766871\S2-1=0.\S2A=0.750236\RMSD=8.196e-09\RMSF=2.869e-06\Di
pole=0.904071,-0.1092652,0.\Quadrupole=3.5715649,8.9054639,-12.4770288
,1.4680842,0.,0.\PG=CS [SG(C27H16N3O1)]\\@

1g

1\1\GINC-GAUSIANDELL\FOpt\UB3LYP\6-31G(2d,p)\C22H13N4O1(2)\PKASZYNSKI\
19-Mar-2025\0\\#P UB3LYP/6-31G(2d,p) FOpt=tight SCF=Direct #P Geom=(No
Distance,NoAngle) fcheck\\5-Quinolinoxy-benzotrazinyl with Smiles rear
rangement, C1\\0,2\N,-0.1399538224,0.1689590391,0.\N,-1.4648104803,2.5
783906709,0.\C,-0.1358640499,2.4524300703,0.\N,0.577909624,1.314852456
6,0.\C,-3.6275291759,-1.0147684504,0.\C,-2.2434784359,-1.0320484908,0.
\C,-1.5296520815,0.1676628497,0.\C,-2.1857112402,1.416140198,0.\C,-3.5
929177531,1.4179226739,0.\C,-4.2909543064,0.2206392381,0.\C,0.53113973

87,-1.0598897298,0.\C,-0.2166304365,-2.2310464441,0.\C,0.4190436316,-3
.4947439338,0.\C,1.8474441694,-3.5510138926,0.\C,1.9467468109,-1.12767
38189,0.\C,0.6683850368,3.7022316792,0.\C,2.069561843,3.6703507009,0.\
C,2.8010781045,4.8530645395,0.\C,2.1460120657,6.0841103524,0.\C,0.7524
647286,6.1240625995,0.\C,0.0177222935,4.9431177159,0.\O,-1.5907838067,
-2.2421037592,0.\H,-4.1011643864,2.3745402259,0.\H,2.4944911477,-0.195
890737,0.\H,2.5761775762,2.7137091403,0.\H,3.8857115127,4.8137449298,0
.\H,0.2354525839,7.0783642552,0.\H,-1.0647977848,4.959336232,0.\H,-5.3
75596487,0.2318006562,0.\H,-4.1688093225,-1.9530088213,0.\H,2.71863941
98,7.0061786451,0.\C,-0.2930669476,-4.7190190232,0.\C,0.4146558164,-5.
8945159598,0.\C,1.8285151582,-5.8431475236,0.\C,2.5853799724,-2.339033
3851,0.\H,-0.0905451857,-6.8539847235,0.\H,2.3990957474,-6.7700241687,
0.\H,-1.3762442622,-4.7084651936,0.\H,3.6666817748,-2.4061706328,0.\N,
2.5285819789,-4.7277512904,0.\\Version=ES64L-G16RevB.01\State=2-A"\HF=
-1140.4190594\S2=0.767234\S2-1=0.\S2A=0.750241\RMSD=1.826e-09\RMSF=2.9
07e-06\Dipole=-0.7035049,-0.7349432,0.\Quadrupole=2.4793522,6.4574277,
-8.9367799,8.2050964,0.,0.\PG=CS [SG(C22H13N4O1)]\\@

1h

1\1\GINC-GAUSIANDELL\FOpt\UB3LYP\6-31G(2d,p)\C22H13N4O1(2)\PKASZYNSKI\
19-Mar-2025\0\\#P UB3LYP/6-31G(2d,p) FOpt=tight SCF=Direct #P Geom=(No
Distance,NoAngle) fcheck\\3-Quinolinoxy-benzotrazinyl with Smiles rear
rangement, C1\\0,2\N,0.3684069862,0.5566490663,0.0186852502\N,-2.13648
43051,1.7285737964,-0.1465403438\C,-1.9234297888,0.4212557447,-0.00860
41699\N,-0.741996863,-0.2125036583,0.0712463173\C,1.2868274629,4.13800
12026,-0.0003713793\C,1.4004954504,2.7600821244,0.059632312\C,0.265845
5797,1.9523170577,-0.0162255185\C,-1.0216840997,2.5195170542,-0.127914
442\C,-1.1242404396,3.9217247548,-0.1939179363\C,0.0135001713,4.708421
2481,-0.1321531028\C,1.6441286498,-0.026924986,0.0217443816\C,2.734159
8891,0.8341384901,0.1432914143\C,4.0433569513,0.331481941,0.2206179572
\C,3.3121393137,-1.8215571949,0.0032000107\C,-3.1140911881,-0.46456540
17,0.0627122284\C,-3.0082513978,-1.7984926708,0.4793735358\C,-4.137627
0729,-2.6082050711,0.5407303007\C,-5.3861074287,-2.099612336,0.1847343
811\C,-5.499474814,-0.7721098002,-0.226282209\C,-4.3733882967,0.042181
4956,-0.2832475637\O,2.6400842763,2.2011757373,0.2107493429\H,4.852240
8524,1.0494344892,0.3372097099\H,-2.0407153396,-2.1881765242,0.7706746
727\H,-4.0431931301,-3.6376191488,0.8711893571\H,-6.4684882275,-0.3690
107758,-0.5026406872\H,-4.4481830269,1.0765364525,-0.5947655658\H,-0.0
756043409,5.7883569026,-0.1799016661\H,2.1826528982,4.7437889908,0.059
5942864\H,-6.2660279149,-2.7332640376,0.2315137151\H,-2.1140044093,4.3
530227283,-0.2810250412\C,1.9306392597,-1.4338916908,-0.0969182614\C,0
.977923657,-2.4605781226,-0.3357207581\C,3.666063574,-3.1892624708,-0.
0906267358\C,2.7151134164,-4.1556031319,-0.3053932053\C,1.3648491,-3.7
786068869,-0.4405111751\H,2.9999291662,-5.1998319821,-0.3808515199\H,0
.6107996399,-4.5353273088,-0.6314288423\H,4.7195345519,-3.4266462123,0
.0025234458\H,-0.0588861521,-2.1933058424,-0.4465484733\N,4.341473389,
-0.9430390226,0.1726259778\\Version=ES64L-G16RevB.01\State=2-A\HF=-114
0.4107378\S2=0.770171\S2-1=0.\S2A=0.750329\RMSD=5.973e-09\RMSF=8.111e-
07\Dipole=-0.2240673,0.2478519,-0.0885695\Quadrupole=-4.5731972,12.054
6264,-7.4814292,4.7721275,0.332473,-0.4226246\PG=C01 [X(C22H13N4O1)]\\
@

1i

1\1\GINC-GAUSIANDELL\FOpt\UB3LYP\6-31G(2d,p)\C29H16N3O1(2)\PKASZYNSKI\
20-Mar-2025\0\\#P UB3LYP/6-31G(2d,p) FOpt=tight SCF=Direct #P Geom=(No
Distance,NoAngle) fcheck\\Photocyclization of 8-PyrenoxyBYT with Smile
s, C1\\0,2\N,-0.8727983255,-0.8568805269,-0.0542587563\N,-3.5541147669
,-0.2039516472,0.2378559074\C,-2.5825626997,0.677851079,0.0102762054\N
,-1.2717186431,0.4331201491,-0.1419374463\C,-2.390321075,-4.2223420422
,0.0324238952\C,-1.4474958163,-3.2142083082,-0.0824920616\C,-1.8234408
539,-1.8776648801,0.0267500243\C,-3.1764649598,-1.5176182986,0.2112150

132\C,-4.1246779431,-2.5499054469,0.3341616857\C,-3.7301110987,-3.8751
989603,0.2483129805\C,1.5711632047,-0.257066655,0.0535253792\C,0.49497
46188,-1.1871572326,-0.0893831318\C,0.806096782,-2.5558598006,-0.28020
62594\C,2.915423737,-0.7305366572,-0.1271511536\C,2.1011491613,-2.9981
550933,-0.4404295602\C,3.1760086481,-2.1058324957,-0.3979780019\H,-5.1
610105139,-2.2700960535,0.4793092534\H,-4.4693182372,-4.6637906266,0.3
39634321\H,-2.0720575562,-5.254045513,-0.0539894698\H,2.2607089519,-4.
0574854228,-0.6064020787\C,1.4072598548,1.1260401852,0.4101468771\C,2.
4698830748,1.9695305335,0.52468921\C,4.0217428287,0.1675684464,-0.0268
609097\C,3.8098929475,1.5392342963,0.2900883199\H,0.4135423524,1.49477
4607,0.5961318361\H,2.3009417608,3.0046787913,0.8070731226\C,4.9076524
268,2.4088504559,0.379874309\C,5.3558822509,-0.2999721983,-0.234157647
2\C,6.4227545947,0.6056533864,-0.1391729629\H,7.4335051973,0.244061364
,-0.302785387\C,6.2001258635,1.9454807507,0.1630081293\H,4.7331421016,
3.4524071941,0.6237912982\H,7.0394255174,2.6296695149,0.2338527123\O,-
0.1468862914,-3.5460489842,-0.3324022122\C,-2.9641190716,2.1113185833,
-0.0874315146\C,-2.0537307989,3.0812611558,-0.5291539612\C,-4.26252559
27,2.505836609,0.2601543451\C,-2.4344785291,4.4165087994,-0.6143498745
\H,-1.054429419,2.7781606633,-0.8162529712\C,-4.637830811,3.8426693172
,0.1785832462\H,-4.9619535063,1.7488214157,0.592132591\C,-3.7260477401
,4.8028018182,-0.2581981662\H,-1.7216397124,5.1569133681,-0.9631685445
\H,-5.645516523,4.135636126,0.4556339036\H,-4.020899303,5.8453061843,-
0.3241756492\C,4.5207294264,-2.5509024273,-0.5978054223\C,5.5666634753
,-1.6852959864,-0.5272727886\H,4.6863086904,-3.6028936035,-0.809335047
6\H,6.5832198806,-2.0332481037,-0.6843054273\\Version=ES64L-G16RevB.01
\State=2-A\HF=-1354.2603225\S2=0.773217\S2-1=0.\S2A=0.750458\RMSD=1.92
2e-09\RMSF=2.817e-07\Dipole=0.735838,-0.3725064,0.015086\Quadrupole=4.
0681568,8.7140566,-12.7822134,2.3432115,-2.0092346,1.1465723\PG=C01 [X
(C29H16N3O1)]\\@

2j(T)

1\1\GINC-GAUSIANDELL\FOpt\UCAM-B3LYP\6-311G(d,p)\C19H13N3O1(3)\PKASZYN
SKI\19-Mar-2025\0\\#P UCAM-B3LYP/6-311G(d,p) FOpt=tight SCF=Direct fre
q=noraman #P Geom=(NoDistance,NoAngle) fcheck SCRF(Solvent=EthylEthano
ate)\\3Ph-benzotrazinyl Phenoxy opt in ground state in AcOEt\\0,3\N,-0
.6516250126,0.3693909935,-1.0043858846\N,-2.7063914873,1.1615292039,0.
5586671042\C,-2.7494284421,0.0416129093,-0.1184755973\N,-1.6905691906,
-0.314106589,-0.8822682368\C,0.7247036022,3.5375833977,0.2635855101\C,
0.657833155,2.3470429636,-0.4357376531\C,-0.4845665834,1.5523133952,-0
.3423652928\C,-1.5834801252,1.9517989727,0.4749324015\C,-1.4840747794,
3.1565685257,1.1674499434\C,-0.3449325257,3.9389641752,1.057914315\C,2
.5510075302,0.3655320273,0.3113280251\C,2.4915632476,0.9225201215,-0.9
577327154\C,3.2993545025,0.4453493007,-1.9820299375\C,3.4297409698,-0.
6852339913,0.5466742629\C,4.1729893938,-0.5994307547,-1.7311962375\C,4
.2419004305,-1.172360941,-0.4661263532\O,1.6768367567,1.9860936515,-1.
2827464532\H,-2.319062138,3.4624833207,1.7835039888\H,-0.2841467544,4.
8744022468,1.5988606017\H,1.616224669,4.1437845812,0.1699574451\H,3.22
78012169,0.90300497,-2.9604362631\H,4.8019422491,-0.9706548606,-2.5313
481132\H,1.9256873748,0.7450811715,1.1085161204\C,-3.9128277575,-0.857
4552016,-0.0772892989\C,-3.9205185948,-2.0420935835,-0.8112051855\C,-5
.0173437501,-0.5206350634,0.7026870967\C,-5.0220517326,-2.8807588686,-
0.7641349794\H,-3.0608551778,-2.3011592991,-1.4168276006\C,-6.11704783
74,-1.3620556391,0.7468692388\H,-4.9985619533,0.4020696878,1.267057644
5\C,-6.1218880976,-2.5426465503,0.0145999574\H,-5.0230904993,-3.800322
8702,-1.3361837994\H,-6.9734927513,-1.0964801056,1.3543169624\H,-6.982
6768725,-3.1993906468,0.0503011613\H,3.4760615015,-1.1215907516,1.5372
032651\H,4.9236888937,-1.9910894494,-0.2735030928\\Version=ES64L-G16Re
vB.01\State=3-A\HF=-970.9445607\S2=2.028475\S2-1=0.\S2A=2.000396\RMSD=
3.620e-09\RMSF=3.186e-07\Dipole=-0.3049741,-0.2247322,0.5274318\Quadru
pole=3.3828577,2.3609995,-5.7438572,3.3525802,-1.3614404,4.9457436\PG=
C01 [X(C19H13N3O1)]\\@

2j(T)-TS

1\1\GINC-GAUSIANDELL\FTS\UCAM-B3LYP\6-311G(d,p)\C19H13N3O1(3)\PKASZYNS
KI\19-Mar-2025\0\\#P UCAM-B3LYP/6-311G(d,p) Opt(QST3) SCF=Direct Geom=
(NoDistance,NoAngle) #P SCRF(Solvent=EthylEthanoate) fcheck freq(noRam
an)\\Phenoxy BT-Ph, cyclizing without Smiles in AcOEt in T state, star
t\\0,3\N,0.2892776863,-0.34497953,-0.4429753282\N,-1.7107178292,1.3786
615852,0.1338293637\C,-1.8961589214,0.0907243845,-0.1045246829\N,-0.92
70913124,-0.7997687872,-0.3770036499\C,2.2383038892,2.7381604042,-0.05
51485717\C,1.9480127431,1.4135143809,-0.3219207123\C,0.629539582,0.958
2618078,-0.24331043\C,-0.4235966035,1.8491820558,0.0749875991\C,-0.108
3869698,3.1842435307,0.3358218302\C,1.2072294886,3.6136778231,0.275404
7485\C,1.9847206895,-1.4544186617,0.0200551555\C,2.7703428803,-0.73046
62334,-0.9118249128\C,3.3102848087,-1.3456161398,-2.0276522902\C,1.780
3899184,-2.8348945315,-0.2098734505\C,3.0751324064,-2.6939870759,-2.23
44244699\C,2.2963452526,-3.4347366664,-1.3300556956\O,2.9791318203,0.6
033862848,-0.7494116834\H,-0.9134241093,3.8608812351,0.5908256142\H,1.
4430134256,4.6500574157,0.4811649052\H,3.2644402058,3.0733269777,-0.12
84642975\H,3.8890859894,-0.7546354425,-2.7250176036\H,3.4862614842,-3.
1785310735,-3.1109347487\H,1.8602513582,-1.0588288518,1.0192295133\C,-
3.2798566091,-0.4470702444,-0.0625283063\C,-3.5325530756,-1.7957829769
,-0.3110403577\C,-4.3468921011,0.4019271839,0.2266894214\C,-4.82874252
74,-2.2849110736,-0.2690825898\H,-2.7036937639,-2.4525976684,-0.537386
7875\C,-5.6426106032,-0.0895480316,0.2679359186\H,-4.1421989332,1.4465
193091,0.4168576568\C,-5.8879710223,-1.4339267429,0.020590577\H,-5.013
4949085,-3.3345428642,-0.464120229\H,-6.4642536542,0.5796450953,0.4937
995539\H,-6.9008325392,-1.8174801813,0.0527043734\H,1.2122822368,-3.40
51806358,0.5130345554\H,2.1266988677,-4.488766091,-1.5090154687\\Versi
on=ES64L-G16RevB.01\State=3-A\HF=-970.9297539\S2=2.052573\S2-1=0.\S2A=
2.001647\RMSD=8.112e-09\RMSF=1.205e-05\Dipole=1.5625455,-1.0033454,-0.
2113056\Quadrupole=2.3689149,5.0922751,-7.4611901,-0.8719093,-3.896423
4,3.5443973\PG=C01 [X(C19H13N3O1)]\\@

2j(T)-cycl

1\1\GINC-GAUSIANDELL\FOpt\UCAM-B3LYP\6-311G(d,p)\C19H13N3O1(3)\PKASZYN
SKI\18-Mar-2025\0\\#P UCAM-B3LYP/6-311G(d,p) FOpt=tight SCF=Direct fre
q(noraman) #P Geom=(NoDistance,NoAngle) fcheck SCRF(Solvent=EthylEthan
oate)\\3Ph-benzotrazinyl phenyl-1-oxy opt cyclized in T state\\0,3\N,0
.5502868387,1.3064643178,-0.5145271921\N,3.1768096693,1.3149724867,0.2
245989473\C,2.471989413,2.4117232201,-0.0251355428\N,1.1812949803,2.47
76301043,-0.3562593631\C,1.0979498338,-2.2928591086,-0.0791740436\C,0.
467777146,-1.1031443103,-0.3783640415\C,1.16494199,0.0998989393,-0.265
2962965\C,2.5140521655,0.1214091158,0.1264898645\C,3.1423972629,-1.095
579327,0.4168087402\C,2.4353752563,-2.2781240558,0.3210683701\C,-0.927
6088822,1.3088057374,-0.5938131208\C,-1.368341549,0.0444741797,-1.2688
260983\C,-2.3210594489,0.0097792441,-2.2338216397\C,-1.4876386679,2.52
94718411,-1.2529162626\C,-2.8902146611,1.2024280143,-2.7159745043\C,-2
.4333226281,2.4468497509,-2.2196616314\O,-0.8314548402,-1.1295580285,-
0.8144169794\H,4.1810263858,-1.0768825491,0.7189373628\H,2.9242993487,
-3.2163110901,0.5507837145\H,0.5445038872,-3.2176750223,-0.1733335499\
H,-1.1397616345,3.4813674658,-0.8802426046\H,-2.6152128292,-0.95218901
11,-2.6348275098\H,-3.6439321002,1.1658603492,-3.4902711958\H,-2.84668
43623,3.3601417499,-2.6309832574\C,3.1696372496,3.7216899239,0.0870087
058\C,2.5271354965,4.9117960149,-0.2539702846\C,4.4879760191,3.7678174
28,0.5373049182\C,3.1915315135,6.1233291097,-0.1439398714\H,1.50629077
79,4.877791385,-0.6086409017\C,5.1501367003,4.9808940716,0.6475331485\
H,4.9808875347,2.8413641612,0.7972794034\C,4.504553432,6.1624190138,0.
3077297152\H,2.6828451676,7.0411461192,-0.4135910304\H,6.1743433563,5.
0036990733,1.0001366267\H,5.0225772041,7.1102209068,0.3931715952\H,-1.
2712090652,1.2775208689,0.4563430094\\Version=ES64L-G16RevB.01\State=3
-A\HF=-970.959884\S2=2.084243\S2-1=0.\S2A=2.003887\RMSD=3.075e-09\RMSF

=5.183e-07\Dipole=-1.1223267,-0.3363596,-0.2291086\Quadrupole=1.214190
4,6.1772744,-7.3914647,-0.4070472,5.749904,-1.0964435\PG=C01 [X(C19H13
N3O1)]\\@

6. References

(1) Singh, H. K.; Kaźmierski, S.; Kaszyński, P. A photo-Smiles rearrangement: Mechanistic Investigation of the formation of Blatter radical helicenes, *J. Org. Chem.* **2025**, *90*, 2386−2392.

(2) Bax, A.; Davis, D. G. MLEV-17-based two-dimensional homonuclear magnetization transfer spectroscopy, *J. Magn. Res.* **1985**, *65*, 355–360.

(3) Hwang, T.-L.; Shaka, A. J. Cross relaxation without TOCSY: transverse rotating-frame Overhauser effect spectroscopy, *J. Am. Chem Soc.* **1992**, *114*, 3157–3159.

(4) **Gaussian 16;** Frisch, M. J. T., G. W.; Schlegel, H. B.; Scuseria, G. E.; Robb, M. A.; Cheeseman, J. R.; Scalmani, G.; Barone, V.; Petersson, G. A.; Nakatsuji, H.; Li, X.; Caricato, M.; Marenich, A. V.; Bloino, J.; Janesko, B. G.; Gomperts, R.; Mennucci, B.; Hratchian, H. P.; Ortiz, J. V.; Izmaylov, A. F.; Sonnenberg, J. L.; Williams-Young, D.; Ding, F.; Lipparini, F.; Egidi, F.; Goings, J.; Peng, B.; Petrone, A.; Henderson, T.; Ranasinghe, D.; Zakrzewski, V. G.; Gao, J.; Rega, N.; Zheng, G.; Liang, W.; Hada, M.; Ehara, M.; Toyota, K.; Fukuda, R.; Hasegawa, J.; Ishida, M.; Nakajima, T.; Honda, Y.; Kitao, O.; Nakai, H.; Vreven, T.; Throssell, K.; Montgomery, Jr., J. A.; Peralta, J. E.; Ogliaro, F.; Bearpark, M. J.; Heyd, J. J.; Brothers, E. N.; Kudin, K. N.; Staroverov, V. N.; Keith, T. A.; Kobayashi, R.; Normand, J.; Raghavachari, K.; Rendell, A. P.; Burant, J. C.; Iyengar, S. S.; Tomasi, J.; Cossi, M.; Millam, J. M.; Klene, M.; Adamo, C.; Cammi, R.; Ochterski, J. W.; Martin, R. L.; Morokuma, K.; Farkas, O.; Foresman, J. B.; Fox, D. J. ; Gaussian, Inc., Wallingford CT, : **2016**.

(5) Cossi, M.; Scalmani, G.; Rega, N.; Barone, V. New developments in the polarizable continuum model for quantum mechanical and classical calculations on molecules in solution, *J. Chem. Phys.* **2002**, *117*, 43–54.

(6) De Vleeschouwer, F.; Chankisjijev, A.; Yang, W.; Geerlings, P.; De Proft, F. Pushing the boundaries of intrinsically stable radicals: Inverse design using the thiadiazinyl radical as a template, *J. Org. Chem.* **2013**, *78*, 3151–3158.

(7) Stratmann, R. E.; Scuseria, G. E.; Frisch, M. J. An efficient implementation of time-dependent density-functional theory for the calculation of excitation energies of large molecules, *J. Chem. Phys.* **1998**, *109*, 8218-8224.
