# Supplementary figures and images for: Photocyclization of 8‑Aryloxybenzo[e][1,2,4]triazines Revisited: Unambiguous Structural Assignment of Planar Blatter Radicals by Correlation NMR Spectroscopy
Source: J Org Chem. 2025 Jun 27;90(27):9425–30. doi: 10.1021/acs.joc.5c00741 (PMC12261333; doi:10.1021/acs.joc.5c00741)

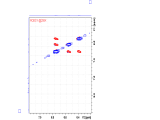

Supplement: Supplementary file 6 [file jo5c00741_si_006.zip › 1e/16/pdata/1/thumb.png]

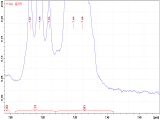

Supplement: Supplementary file 6 [file jo5c00741_si_006.zip › 1e/10/pdata/1/thumb.png]

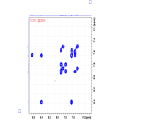

Supplement: Supplementary file 6 [file jo5c00741_si_006.zip › 1e/15/pdata/1/thumb.png]

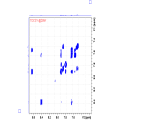

Supplement: Supplementary file 6 [file jo5c00741_si_006.zip › 1e/13/pdata/1/thumb.png]

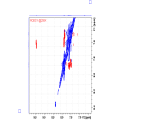

Supplement: Supplementary file 7 [file jo5c00741_si_007.zip › 1f/16/pdata/1/thumb.png]

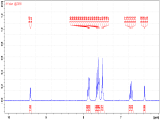

Supplement: Supplementary file 7 [file jo5c00741_si_007.zip › 1f/10/pdata/1/thumb.png]

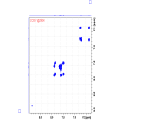

Supplement: Supplementary file 7 [file jo5c00741_si_007.zip › 1f/15/pdata/1/thumb.png]

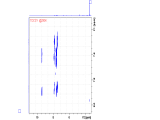

Supplement: Supplementary file 7 [file jo5c00741_si_007.zip › 1f/13/pdata/1/thumb.png]
